# A regioselective C7 bromination and C7 palladium-catalyzed Suzuki–Miyaura cross-coupling arylation of 4-substituted NH-free indazoles†

**DOI:** 10.1039/d0ra08598g

**Published:** 2021-02-10

**Authors:** Khalid Boujdi, Nabil El Brahmi, Jérôme Graton, Didier Dubreuil, Sylvain Collet, Monique Mathé-Allainmat, Mohamed Akssira, Jacques Lebreton, Saïd El Kazzouli

**Affiliations:** Euromed Research Center, School of Engineering in Biomedical and Biotechnology, Euromed University of Fes (UEMF) Route de Meknes 30000 Fez Morocco s.elkazzouli@ueuromed.org; Laboratoire CEISAM–UMR 6230, Université de Nantes, CNRS, Faculté des Sciences et des Techniques 2 rue de la Houssinière, BP 92208 44322 Nantes Cedex 3 France; Faculty of Sciences and Technologies Mohammedia, University Hassan 2, URAC 22 FSTM University Hassan II – Casablanca BP 146 28800 Mohammedia Morocco

## Abstract

A direct and efficient regioselective C7-bromination of 4-substituted 1*H*-indazole has been achieved. Subsequently, a successful palladium-mediated Suzuki–Miyaura reaction of C7-bromo-4-substituted-1*H*-indazoles with boronic acids has been performed under optimized reaction conditions. A series of new C7 arylated 4-substituted 1*H*-indazoles was obtained in moderate to good yields.

## Abstract 2

A regioselective C7-bromination of 4-substituted 1*H*-indazoles followed by a palladium-catalyzed Suzuki–Miyaura reaction with boronic acids is described.

## Graphical abstract

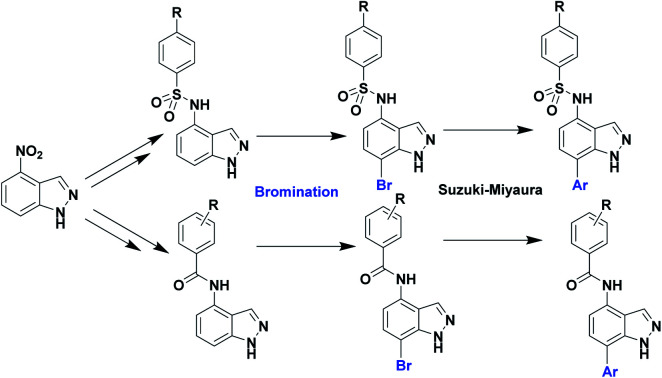

## Introduction

The aryl-heteroaryl compounds are an important class of organic entities used as promising building blocks of many biologically active molecules and drugs^1–6^ and the ability to combine aryl and heteroaryl fragments by the formation of new C–C bonds^7–11^ is an important and challenging field in organic chemistry. The palladium-catalyzed Suzuki–Miyaura coupling process^12–16^ is probably one of the most efficient methods nowadays to create C(sp^2^)–C(sp^2^) bonds because of its mild reaction conditions, broad substrate scope, broad functional group tolerance, and the high air and water stability of the boronic acids.^12,17–20^

Indazoles are often the key fragments in several important compounds, with a broad range of biological activities for anticancer,^21–23^ HIV-protease inhibition,^24^ antimicrobial^25^ and anti-inflammatory^26^ purposes. For this reason, various procedures have been developed for their synthesis^27–30^ and functionalization.^31–45^

In particular, indazoles and bioisosteres containing sulfonamide moieties on either the C7 or C4 position have shown interesting anticancer activities.^46–48^ For these reasons we decided to introduce sulfonamides at position C4 of the indazole ring prior to the functionalization at the C7 position in order to give access to a diversity of new potentially bioactive compounds.

Recently, our group and others were actively involved in the functionalization of NH-free or protected 1*H* and 2*H*-indazoles. Despite the recent advances made in this field, namely the direct C3 and C7-arylations^32,49–51^ and the Suzuki–Miyaura coupling at C3, C4, C5 and C6 positions,^31,52–54^ to date, no example of selective arylation of NH-free or protected indazoles using Suzuki–Miyaura process, has been described at the C7 position. It is important to note that we previously reported only two examples of C7 direct arylation of indazoles containing a C4 nitro group in which C3 position was already substituted with a phenyl group. Moreover, during these investigations with nitro substrates, we were unable to introduce heteroaryl substituents.^55^ At this point, it was clear to us that the higher reactivity of the C3 position over C7 unsubstituted indazole derivatives could be a limitation to efficiently prepare new C7 substituted and C3 free scaffolds.

To bypass this limitation, we examine herein the influence of the electronic properties of some C4 sulfonamido- or amido-substituents at the 1*H*-indazole nucleus, on the course of a regioselective C7-halogenation, followed by Suzuki–Miyaura coupling reactions, aiming at developing novel series of 7-aryl-4-sulfonamido or 7-aryl-4-amido-1*H*-indazole compounds.

## Results and discussion

**Scheme 1:**
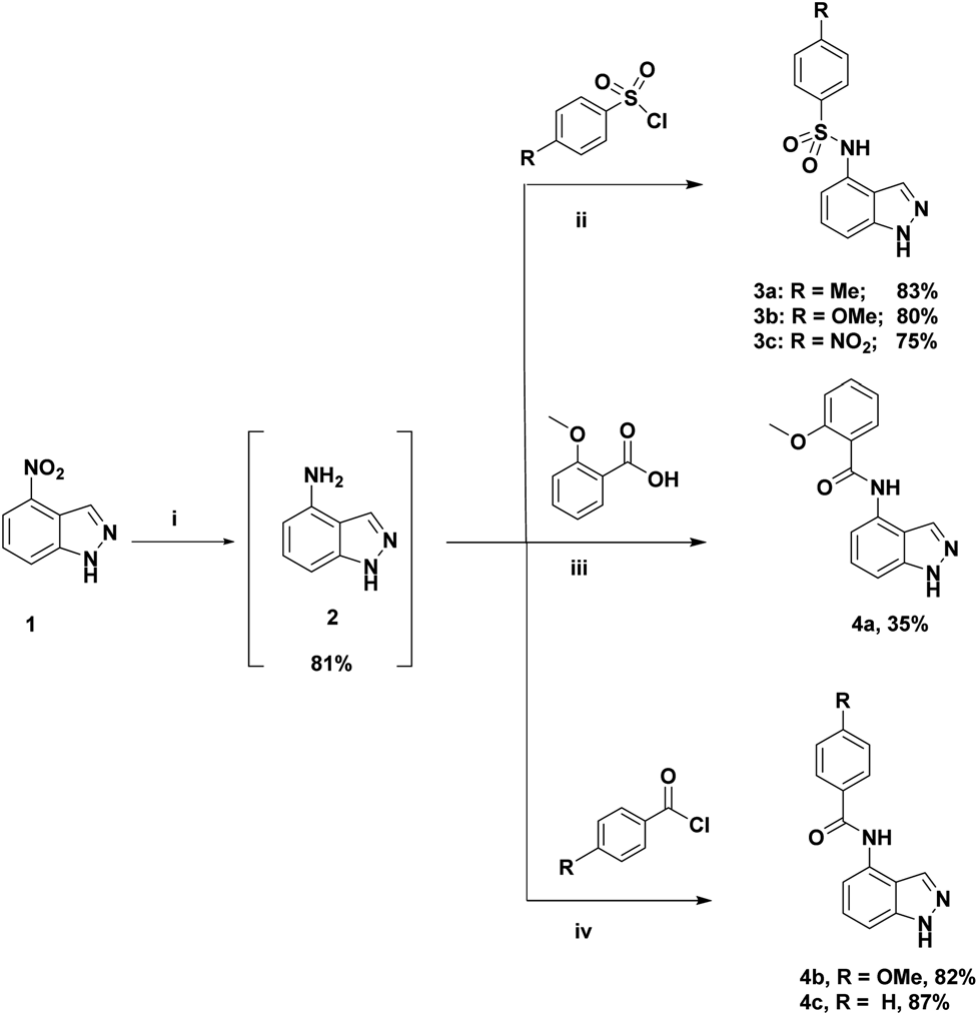
Reagents and conditions for the synthesis of 3a–c and 4a–c. Reaction conditions. (i) Fe/NH_4_Cl (6/10 equiv.), EtOH, rt for 3 h, 81%; (ii) ArSO_2_Cl (1 equiv.), pyridine, 24 h, 75–83%; (iii) *o*-methoxy benzoic acid (1 equiv.), TBTU (1 equiv.), DIPEA (3 equiv.), DMF, rt for 16 h, 35%. (iv) *p*-methoxy benzoyl chloride (1.1 equiv.), DIPEA (2 equiv.), DCM, 0 °C-rt for 18 h, 82%.

The first step in our investigation pathway was to prepare the 4-substituted 1*H*-indazoles 3a–c, 4a–c, used as starting materials in this study. They were synthesized following known procedures starting from 4-nitroindazole according to the Scheme 1.^47,56^ The sulfonylation of free 4-amino-indazole 2 with one equivalent of the selected sulfonyl chlorides gave the expected sulfonamides 3a–c in 75–83% yields. The 4-amino acylation of indazole 2 with a carboxylic acid in the presence of coupling reagents afforded amide 4a with a low yield.^56^ Satisfyingly, in dichloromethane as the solvent and with acyl chloride as the acylating agent, the yield could be optimized, due to the precipitation of the expected amide 4b and 4c during the reaction, probably avoiding the formation of N-1 acyl side-product. These compounds were isolated by simple filtration with high yields of 82% and 87%, respectively.

**Fig. 1:**
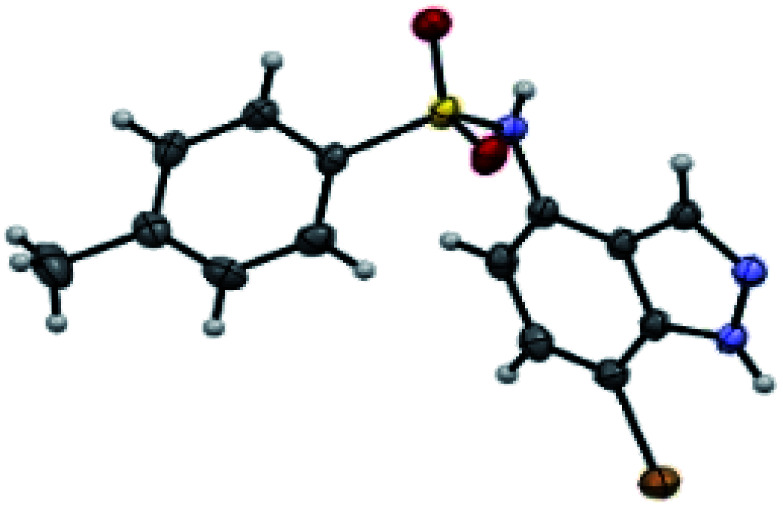
Crystal structure of compound 5a.

The regioselective C7 bromination of *N*-(1*H*-indazol-4-yl)-4-methylbenzenesulfonamide 3a, used as model substrate, was attempted with *N*-bromosuccinimide (NBS)^57,58^ as depicted in Table 1. Compared to room temperature conditions (Table 1, entry 1), we were pleased to find that the treatment of *N*-(1*H*-indazol-4-yl)-4-methylbenzenesulfonamide 3a with 1.1 equivalents of NBS in DMF at 80 °C provided the desired C7 halogenated product 5a in 84% yield along with 10% of the 5,7-dibrominated compound 6a, (Table 1, entry 2). Testing microwave activation conditions afforded degradation (Table 1, entry 3). Moreover, when 2 equivalents of NBS were used, the 5,7-dibrominated compound 6a was obtained with 88% yield, without identification of any 3-brominated derivative (Table 1, entry 4). Based on previous results obtained for indazole halogenation in basic conditions,^46^ we observed in this case a rapid formation of the dibrominated compound 6a, in the presence of 2 equivalents of NaOH or KOH (Table 1, entries 5 and 6). The structure of the compound 5a as its 7-bromo-1*H*-indazole form was proved by X-ray diffraction analysis (Fig. 1) (see the ESI†).

**Table 1:** Bromination study of compound 3a with NBS

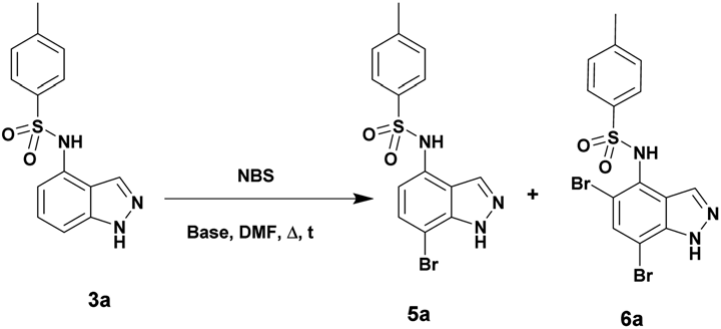					
Entry	*T* (°C)	NBS (equiv.)	*t* (h)	Base	Yields^a^5a/6a
1	rt	1.1	18	None	26/4
2	80	1.1	18	None	**84**/10^b^
3	120^c^	1.1	0.5	None	Degradation
4	Reflux	2.0	18	None	tr^e^/**88**
5	80	1.1	18	NaOH^d^	45/28
6	80	1.1	18	KOH^c^	18/**45**

a Yields after column chromatography purification.

b Reaction conditions optimized for 5a : 3a (1 mmol), NBS (1.1 mmol), DMF (5 mL), 80 °C for 18 h.

c MW = microwaves.

d 2 equivalents of base.

e tr = traces.

**Scheme 2:**
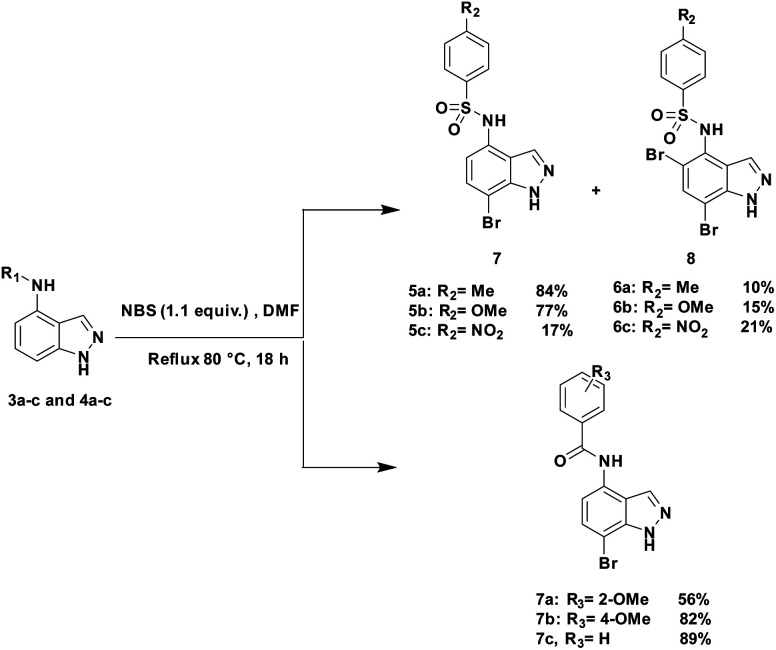
Bromination of compounds 3a–c and 4a–c with NBS.

With this sequence in hand, we set out to extend the halogenation to the series of indazole derivatives containing sulfonamide or amide groups at C4 position (Scheme 2). These experiments highlighted the regioselectivity of the halogenation reaction with NBS but also the influence of the nature and the electronic effect of the C4 substituent group. Sulfonamides with electron-donating groups such as methyl or methoxy at *para*-position of the phenylsulfonyl group gave the desired C7 monohalogenated products 5 in high yields along with small amounts of 5,7-dihalogenated compounds 6 (5a : 6a, 84%/10% and 5b : 6b, 77%/15%). Strong electron-withdrawing substituents such as NO_2_, also gave a mixture of both mono- and dihalogenated compounds but with a drastic decrease of the C7-mono brominated expected product 5c (5c, 17%; 6c, 21%) (Scheme 2). Satisfyingly, the bromination reaction with indazoles 4a–c containing benzamide groups at C4 position and bearing electron-donating group at either the *ortho*- or *para*-position of the aryl ring, gave only the C7 halogenated products 7a–c, and no traces of dihalogenated products were observed. In the case of the bromination reaction with 4a, the reaction was not total and starting material 4a was recovered.

**Fig. 2:**
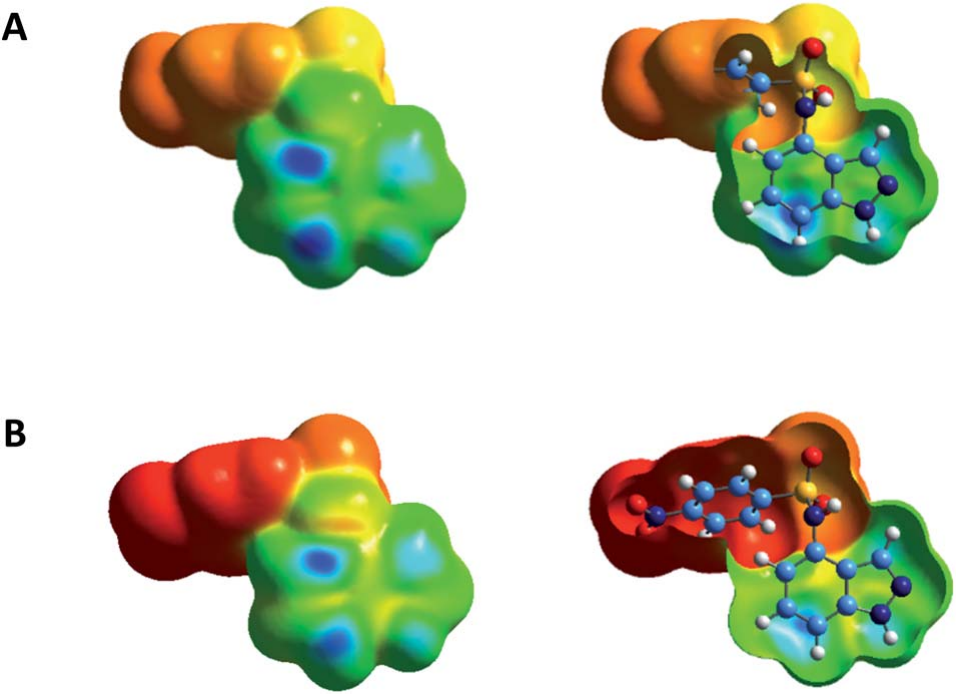
Electrophilicity index fk^+^ Fukui function of compounds 3a (A) and 3c (B), shown on the electron density isosurfaces (0.001 e bohr^−3^) and calculated at the MN15/6-31++G(d, p) level of theory. The sites in blue are the most prone to a nucleophilic attack.

**Fig. 3:**
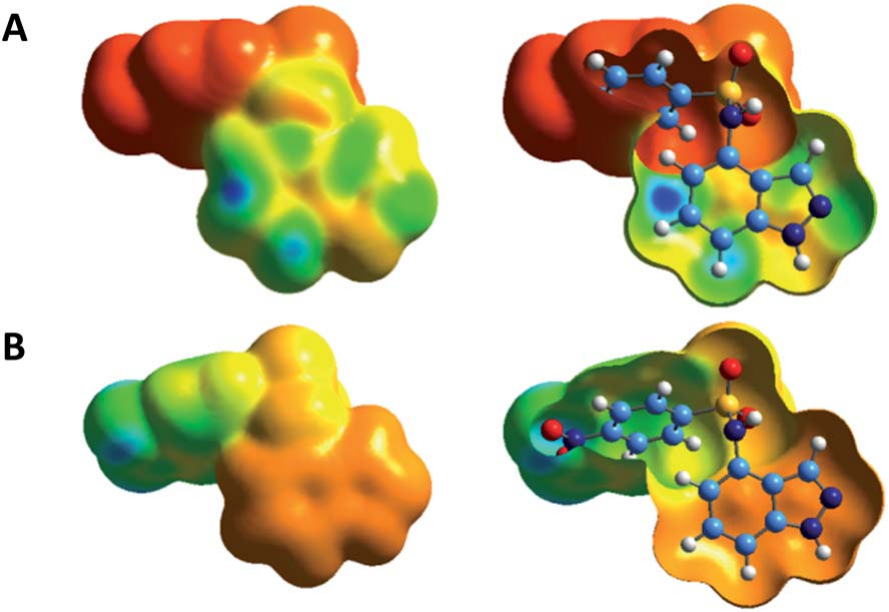
Nucleophilicity index fk^−^ Fukui function of compounds 3a (A) and 3c (B), shown on the electron density isosurfaces (0.001 e bohr^−3^) and calculated at the MN15/6-31++G(d, p) level of theory. The sites in blue are the most prone to an electrophilic attack.

DFT calculations have been performed to identify the main electrophilic, nucleophilic or radical reaction sites of compound 3a and 3c, with the aim to rationalize the preferred site of bromination experimentally observed. For compound 3a, the projection of the fk^+^ Fukui function on the isodensity surface suggests that this compound will preferentially undergo a nucleophilic attack from a Br^−^ anion on its C5 and C7 carbon atoms, as illustrated by the regions in blue in Fig. 2A. Likewise, compound 3c is predicted to be the subject of a nucleophilic attack on the same sites (Fig. 2B). If experimentally, the C7 atom is the preferred site of bromination, it can be due to the greater steric hindrance at C5 on the second face of the aromatic moiety. Indeed, only one electrophilic site on C7 can be detected on the back side of the indazole moieties of 3a and 3c, as shown by the open surfaces in Fig. 2. Considering an electrophilic attack from a Br^+^ cation, the representation of the fk^−^ Fukui function is less convincing, with a main site of interaction that would be between C5 and C6 for compound 3a, and a secondary site on C7 (Fig. 3A). Furthermore, none of the indazole carbon atoms of 3c seem to be prone to such an electrophilic attack (Fig. 3B). Similar trends are found with the assumption of a radical attack (Fig. S1, see ESI†), without any favorable carbon sites in 3c and, for 3a, two active sites on the C7 atom for the former and between the C5 and C6 atoms for the latter. The comparison of these different theoretical models mainly suggests that C3 bromination is never predicted, as experimentally observed. Additionally, following our bromination conditions at high temperature with NBS involving an electrophilic Br^+^, we could observe a higher reactivity of compound 3a over compound 3c.

A direct and efficient regioselective C7-bromination of 4-substituted 1*H*-indazole has been achieved. Subsequently, a successful palladium-mediated Suzuki–Miyaura reaction of C7-bromo-4-substituted-1*H*-indazoles with boronic acids has been performed under optimized reaction conditions. A series of new C7 arylated 4-substituted 1*H*-indazoles was obtained in moderate to good yields.

Once the halogenation has been carried out on 3a, the obtained *N*-(7-bromo-1*H*-indazol-4-yl)-4-methylbenzenesulfonamide 5a was employed to optimize the Suzuki–Miyaura reaction with (4-methoxyphenyl)boronic acid as coupling partner under various reaction conditions exploring the effects of different bases, catalysts, solvents, and reaction times (Table 2). We began this study using 10 mol% of PdCl_2_(PPh_3_)_2_ as catalyst and K_2_CO_3_ or Cs_2_CO_3_ as base in DMF at reflux for 48 h. These conditions failed to give the desired product 8ab (entries 1 and 2). Using Pd(PPh_3_)_4_ as the catalyst instead of PdCl_2_(PPh_3_)_2_, only traces of the desired product 8ab were detected along with dehalogenated product 3a (entries 3 and 4). Unfortunately, only a modest yield of 11% of coupled product 8ab was obtained carrying out the reaction under microwaves irradiation for 2 h (entry 5). Then various parameters such as solvents (pure or as a mixture), temperature, pressure (sealed tube) or microwave activation conditions were changed (entries 6–10). We found that protocols realized in a mixture of dioxane/EtOH/H_2_O (3/1.5/0.5) as solvents in a sealed tube either under conventional heating or microwave irradiation provided the coupled product 8ab in a good yield (70%) together with traces of both starting material 5a and dehalogenated product 3a (entries 9 and 10).

**Table 2:** Optimization of Suzuki–Miyaura reaction conditions^a^

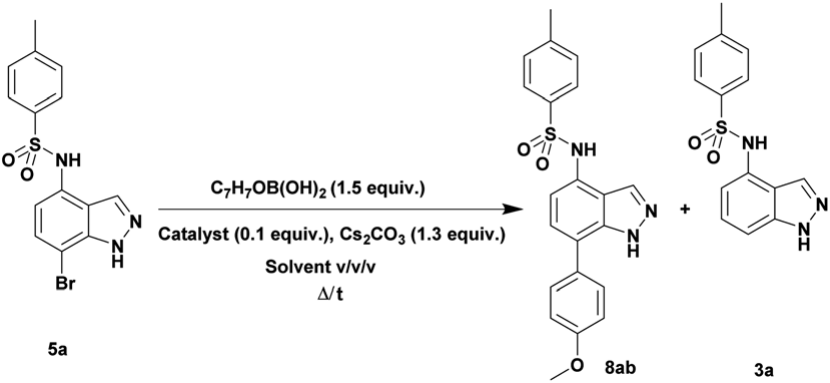				
Entry	Catalyst 10 mol%	*T* (°C)/*t* (h)	Solvent (v/v)	Yields% 3a/5a/8ab
1^b^	PdCl_2_(PPh_3_)_2_	Reflux/48	DMF	0/100/0
2	PdCl_2_(PPh_3_)_2_	Reflux/48	DMF	0/100/0
3^b^	Pd(PPh_3_)_4_	Reflux/48	DMF	18/75/tr^c^
4	Pd(PPh_3_)_4_	Reflux/48	DMF	tr/80/tr
5	Pd(PPh_3_)_4_	140 MW/2	DMF	68/tr/11
6	Pd(PPh_3_)_4_	Reflux/2	Dioxane	0/100/0
7	Pd(PPh_3_)_4_	Reflux/48	Dioxane/EtOH 3/1	14/69/tr
8	Pd(PPh_3_)_4_	Reflux/48	Dioxane/EtOH/H_2_O 3/1.5/0.5	14/32/46
9	Pd(PPh_3_)_4_	140 MW/2	Dioxane/EtOH/H_2_O 3/1.5/0.5	tr/tr/**70**
10	Pd(PPh_3_)_4_	140 sealed tube/2	Dioxane/EtOH/H_2_O 3/1.5/0.5	tr/tr/**70**

a Optimized conditions: (5a) (1 mmol), Pd(PPh_3_)_4_ (10 mol%), Cs_2_CO_3_ (1.3 mmol), dioxane/EtOH/H_2_O (3/1.5/0.5 mL), 140 °C for 4 h. Yields of products after column chromatography purification.

b K_2_CO_3_ was used as base.

c tr = traces.

The optimized reaction conditions (1 equiv. of 5a–c, 10 mol% of Pd(PPh_3_)_4_, 1.3 equiv. of Cs_2_CO_3_, dioxane/EtOH/H_2_O (3/1.5/0.5 mL), 140 °C (2 h under MW or 4 h in a sealed tube)) were used to explore the substrate scope and limitations. A variety of aryl and heteroaryl boronic acids were successfully coupled with the 4-substituted-7-bromo-1*H*-indazoles 5a–c (Table 3). The reactions of 5a and 5b bearing electron donating groups with phenylboronic acids bearing also electron donating groups resulted in the formation of the desired products 8aa–8ac and 8ba–8bc in good yields (Table 3, entries 1–3 and 7–9). The aryl boronic acid bearing electron-withdrawing group NO_2_ at the C4 position, was also efficiently coupled with 7a and 5b, giving 8ad and 8bd in 78% and 75% yield, respectively (Table 3, entries 4 and 10). Additionally, the reaction of indazole 5c containing NO_2_ on the sulfonamide moiety reacted with 4-methoxyphenyl boronic acid, to give the coupled product 8cb in 71% yield (entry 12). These optimized conditions were also successfully applied to couple heteroaryl boronic acids such as thienyl and furyl-boronic acid. Thus, these derivatives were coupled with 5a and 5b to lead to 8ae and 8be in 75% and 72% yield, respectively (Table 3, entries 5 and 11). Comparatively, 2-thienyl boronic acid was coupled to 5a to give 8af with an excellent yield (80%, entry 6). It is noticed that except for the boronic acids bearing alkyl groups which gave the desired products 8aa and 8ba in moderate yields (entries 1 and 7, Table 3), the Suzuki–Miyaura cross-coupling reaction was not influenced by electronic or steric hindrance of the substituents on the boronic acid partners.

**Table 3:** Suzuki–Miyaura coupling of 7-bromo-4-sulfonamido-1*H*-indazoles 5a–c to aryl boronic acidsab

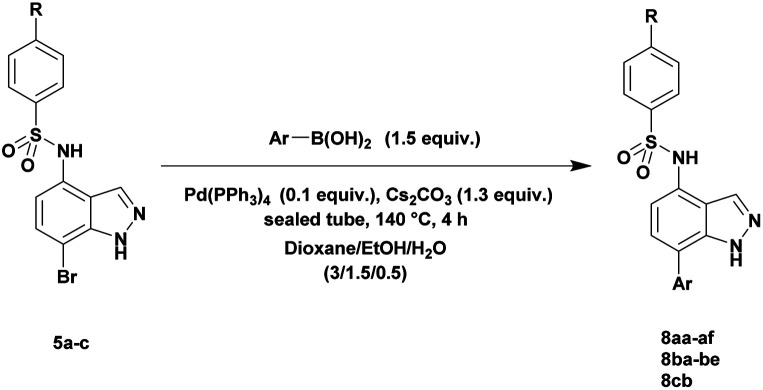					
Entry			ArB(OH)_2_	Product	Yield (%)
1	5a	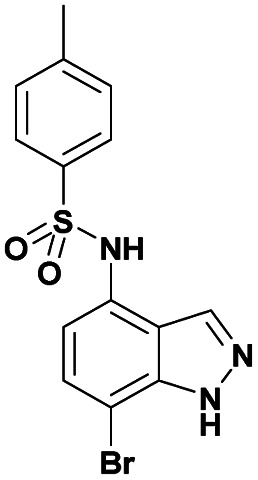	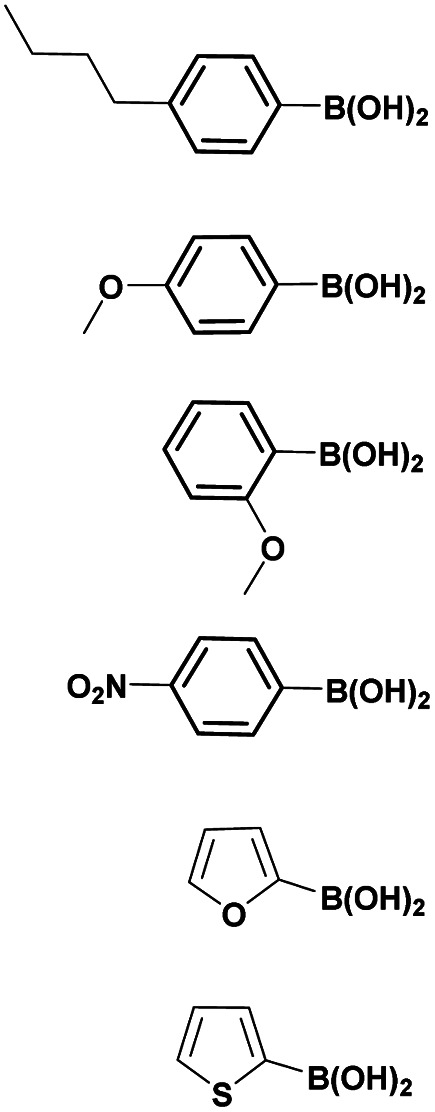	8aa	62
2				8ab	70
3				8ac	76 (70)^a^
4				8ad	78
5				8ae	75 (72)^a^
6				8af	80
7	5b	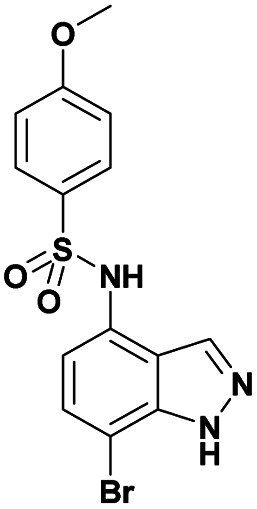	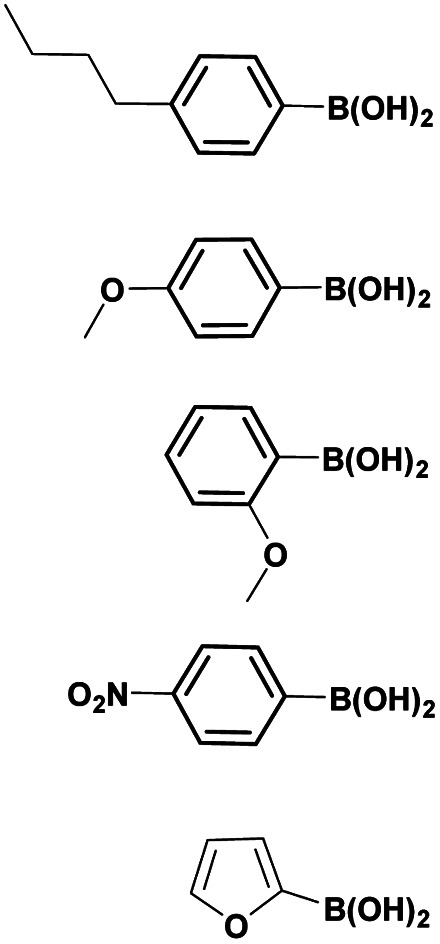	8ba	57
8				8bb	81
9				8bc	75
10				8bd	75 (74)^a^
11				8be	72
12	5c	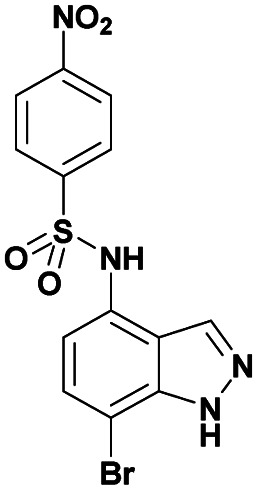	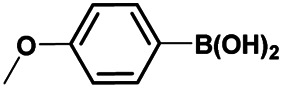	8cb	71

a Sealed tube, 2 h under MW.

Finally, using the optimized conditions, the scope of this protocol was also extended to 4-amido-7-bromo-1*H*-indazole 7a–c, in order to examine the effect of the nature of the functional group at C4 on the Suzuki–Miyaura reaction. With an amido substituent at C4 position, the starting material 7 efficiently reacted with various boronic acids to provide the corresponding C7 arylated products 9 in good yields either with aryl (Table 4, entries 1–3, 6–8) or heteroaryl reagents (Table 4, entries 4 and 5). In this case, the reaction yields were not influenced by the electronic or steric hindrance of the substituents on the boronic acids. It is noticed so that in the case of unprotected 7-bromo-1*H*-indazoles bearing benzamide groups at C4 position, the heteroaryl boronic acids as coupling partners gave excellent yields.

**Table 4:** Suzuki–Miyaura coupling of 7-bromo-4-carboxamido-indazoles 7a–c to aryl boronic acids

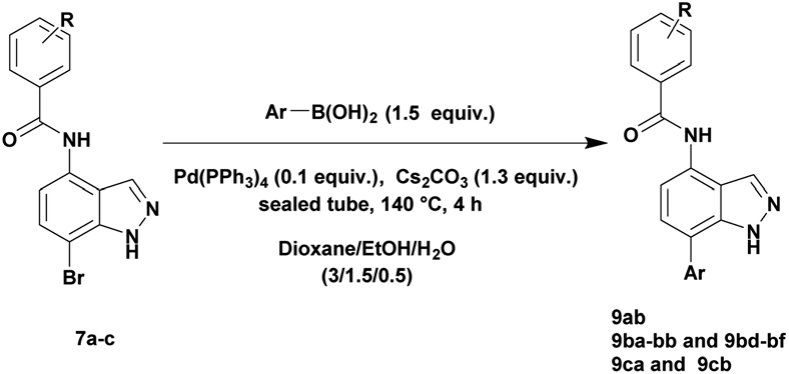					
Entry			ArB(OH)_2_	Product	Yield (%)
1	7a	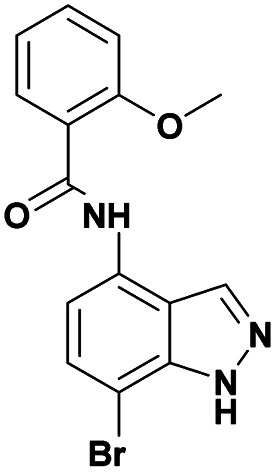	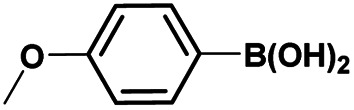	9ab	78
2	7b	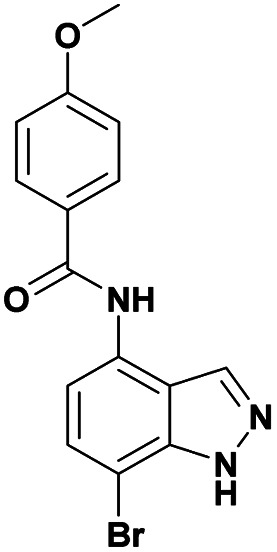	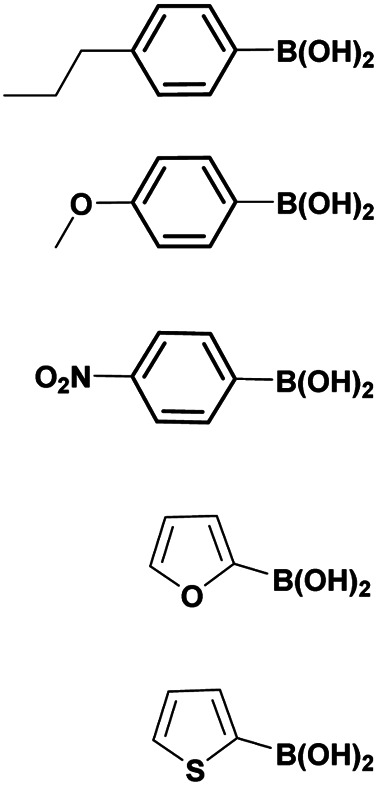	9ba	70
3				9bb	82
4				9bd	76
5				9be	91
6				9bf	85
7	7c	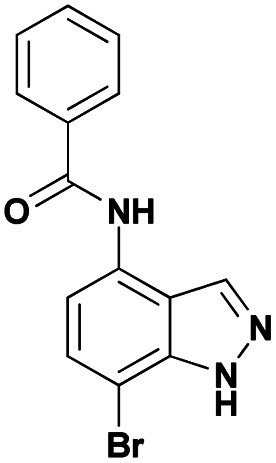	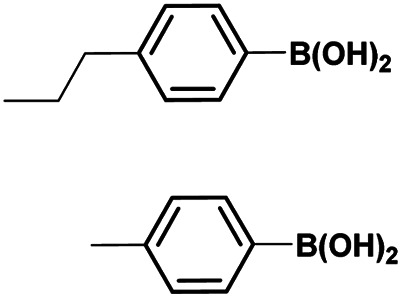	9ca	75
8				9cb	82

## Conclusions

We have prepared a novel series of C7 substituted unprotected NH indazoles in a two steps manner from 4-sulfonamido-1*H*-indazoles. A simple, fast and regioselective bromination reaction at the C7 position of the 4-sulfonamido-1*H*-indazoles was observed with *N*-bromosuccinimide and a computational study was performed to estimate the reactivity of the 4-sulfonamido NH-indazole ring. From the bromo NH-indazole precursors, a Suzuki–Miyaura cross-coupling reaction with a set of aryl boronic acids afforded the expected C7 (hetero)arylated NH-indazole derivatives, in moderate to excellent yields, regardless of any electronic influence or steric hindrance by the substituent on the boronic partner. Then, we have shown also that the bromination of unprotected NH indazoles bearing benzamide groups at C4 position took place in regioselective manner at C7 position. Again, the Suzuki–Miyaura cross-coupling reaction with various (hetero)aryl boronic acids led to the desired C7 (hetero)arylated NH-indazole derivatives in good yields.

## Conflicts of interest

There are no conflicts to declare.

## Supplementary Material

RA-011-D0RA08598G-s001

RA-011-D0RA08598G-s002
