# Supplementary material for: A regioselective C7 bromination and C7 palladium-catalyzed Suzuki–Miyaura cross-coupling arylation of 4-substituted NH-free indazoles
Source: RSC Adv. 2021 Feb 10;11(12):7107–14. doi: 10.1039/d0ra08598g (PMC8694914; doi:10.1039/d0ra08598g)

Electronic Supplementary Material (ESI) for RSC Advances.
This journal is © The Royal Society of Chemistry 2021

A Regioselective C7 Bromination and C7 Palladium-Catalyzed Suzuki–Miyaura Cross-coupling Arylation of 4-Substituted NH-Free Indazoles

Khalid Boujdi,[a,b,c] Nabil El Brahmi,[a] Jérôme Graton,[b] Didier Dubreuil,[b] Sylvain Collet,[b] Monique Mathé-Allainmat,*[b] Mohamed Akssira,[c] Jacques Lebreton[b] and Saïd El Kazzouli*[a]

[a] *Euromed Research Center, School of Engineering in Biomedical and Biotechnology, Euromed University of Fes, Route de Meknes, 30000 Fez, Morocco.*
[b] *Laboratoire CEISAM–UMR 6230, Université de Nantes, CNRS, Faculté des Sciences et des Techniques, 2 rue de la Houssinière, BP 92208, 44322 Nantes Cedex 3, France.*
[c] *Faculty of Sciences and Technologies Mohammedia, University Hassan 2, URAC 22 FSTM University Hassan II – Casablanca, BP 146, 28800 Mohammedia, Morocco.*

Supporting Information

Table of Contents

I. Experimental section 2
II. X-Ray analysis and structural data of compound *5a* 15
III. Computational procedure and representation of the f_k^- Fukui function for a radical attack 16
IV. References 18
V. ^{1}H and ^{13}C NMR Spectra 19

I. Experimental section

1. General methods

TLC was performed on silica-covered aluminum (Kieselgel 60F254, Merck). Eluted TLCs were visualized using UV radiation (λ = 254 nm). **Flash column chromatography** was performed on silica gel 60 (ACC 40–63 µm 5SDS-CarloErba). **Infrared spectroscopic analyses** were recorded on an IRTF Bruker Tensor Spectrophotometer Specac Quest ATK system (samples were neat). **NMR spectra** were recorded on a Bruker AC300 (300 MHz for ^{1}H and 75 MHz for ^{13}C) or on a Bruker 400 (400 MHz for ^{1}H and 100.6 MHz for ^{13}C) at room temperature, on samples dissolved in an appropriate deuterated solvent. Tetramethylsilane (TMS) for ^{1}H and the deuterated solvent signal for ^{13}C were used as references. Assignments of individual signals were carried out using COSY, NOESY, HSQC, HMBC and DEPT experiments. Chemical shifts (δ) are expressed in parts per million (ppm), and coupling constants (*J*) in Hertz (Hz). **High-resolution mass spectrometry (HRMS)** was recorded on a Waters Xevo GL-XS Qtof spectrometer coupled with an Acquity H-Class LC apparatus. Ionization sources were performed with the available methods among electrospray ionization (ESI$^+$ or ESI$^-$) or Atmospheric Solid Analysis Probe (ASAP+ or ASAP-). For HRMS, a tolerance of 5 ppm was applied to discriminate the difference between calculated and experimental values. **X-ray single-crystal diffraction analysis** was performed on the SFR MATRIX platform in Angers (49, France) and data were collected at 294K on a Rigaku oxford diffraction SuperNova diffractometer equipped with Atlas CCD detector and micro-focus Cu-Kα radiation (λ = 1.54184 Å). **Fusion points** were recorded with an Electrothermal Digital Melting Point apparatus from Cole – Parmer.

2. Materials

Unless otherwise noted, all chemicals, starting materials and solvents used in this study were commercially available from Sigma-Aldrich or Alfa Aesar and used without further purification. The 4-nitro-1*H*-indazole was prepared as described in the literature.[58]

3. General synthesis

General synthesis of N-(1*H*-Indazol-4-yl)-4-sulfonamides (3)

4-nitro-1*H*-indazole **1** (1 equiv.) was introduced in a round-bottomed flask and ethanol (20 mL) and water (10 mL) were added as solvents. Ammonium chloride (10 equiv.) was added and the solution was stirred for 5 min at room temperature. To this content, the iron powder (10 equiv.) was added and stirring was carried on at room temperature for 3 h. The mixture was filtered and the residue was washed with ethanol. The filtrate was concentrated under reduced pressure and then dissolved in ethyl acetate. The organic phase was washed with brine, dried with anhydrous Na_2SO_4 and concentrated. The crude 4-amino-1*H*-indazole **2** was dissolved in pyridine, then directly reacted with substituted benzenesulfonyl chloride (1 equiv.) at room temperature for 24 h. The reaction mixture was concentrated under vacuum, the resulting residue was purified by column chromatography on silica gel (eluted with EtOAc/Cyclohexane, 1/9).

N-(1*H*-4-Indazolyl)-4-methylbenzenesulfonamide (3a).

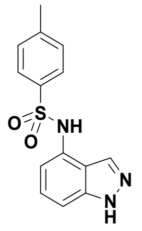

It was obtained as a white solid. 83% yield. mp: 203.0-205.0. (lit[47], Mp 170-172 °C). ^{1}H NMR (300 MHz, DMSO-d_6): δ 13.04 (s, 1H), 10.51 (s, 1H), 8.20 (d, *J* = 1.5 Hz, 1H), 7.67 (d, *J* = 8.3 Hz, 2H), 7.29 (d, *J* = 7.7 Hz, 1H), 7.24 – 7.11 (m, 2H), 6.90 (dd, *J* = 5.7, 2.5 Hz, 1H), 2.28 (s, 3H). ^{13}C NMR (75 MHz, DMSO-d_6): δ 143.2, 140.9, 136.8, 131.8, 129.9, 129.5 (2C), 126.7 (2C), 126.4, 116.8, 110.4, 106.1, 20.9. IR (neat): $ν_{max}$ 3420, 3156, 1601, 1234, 1156, cm^{-1}. HRMS (ESI$^+$): *m/z* calcd for $C_{14}H_{14}N_3O_2S$ [M + H]$^+$: 288.0807, found 288.0803.

N-(1*H*-4-Indazolyl)-4-methoxylbenzenesulfonamide (3b).

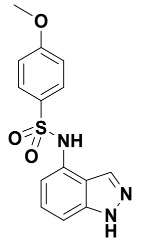

It was obtained as a white solid. 80% yield. mp: 185.5-187.5 °C. ^{1}H NMR (300 MHz, DMSO-d_6): δ 13.04 (s, 1H), 10.44 (s, 1H), 8.21 (s, 1H), 7.72 (d, *J* = 8.9 Hz, 2H), 7.19 –7.18 (m, 2H), 7.01 (d, *J* = 8.9 Hz, 2H), 6.90 (dd, *J* = 4.9, 3.3 Hz, 1H), 3.76 (s, 3H). ^{13}C NMR (75 MHz, DMSO-d_6): δ 162.3, 140.8, 131.8, 131.2, 130.0, 128.8 (2C), 126.4, 116.7, 114.2 (2C), 110.3, 106.0, 55.5. IR (neat): ν_{max} 3356, 3285, 1593, 1324, 1260, 1150, cm^{-1}. HRMS (ESI$^-$): *m/z* calcd for $C_{14}H_{12}N_3O_3S$ [M - H]$^-$: 302.0599, found 302.0597.

N-(1*H*-4-Indazolyl)-4-nitrobenzenesulfonamide (3c).

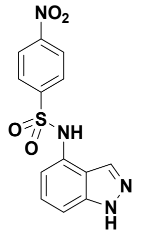

It was obtained as a yellow solid. 75% yield. mp: 272.4-274.0 °C. ^{1}H NMR (300 MHz, DMSO-d_6): δ 13.11 (s, 1H), 10.90 (s, 1H), 8.33 (d, *J* = 8.9 Hz, 2H), 8.14 (s, 1H), 8.01 (d, *J* = 8.9 Hz, 2H), 7.27–7.18 (m, 2H), 6.89 (dd, *J* = 7.1, 1.0 Hz, 1H). ^{13}C NMR (101 MHz, DMSO-d_6): δ 149.7, 145.0, 141.0, 131.7, 128.9, 128.2 (2C), 126.4, 124.5 (2C), 117.4, 111.9, 107.1. IR (neat): ν_{max} 3340, 3235, 1595, 1335, 1160, cm^{-1}. HRMS (ESI$^-$): *m/z* calcd for $C_{13}H_9N_4O_4S$ [M - H]$^-$: 317.0345 found 317.0342.

N-(1*H*-4-Indazolyl)-2-methoxybenzamide (4a).

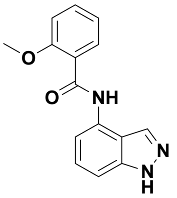

TBTU (0.482 g, 1.5 mmol) and DIPEA (0.8 mL, 4.5 mmol) in DMF (2.5 mL) were added to a solution of 2-methoxybenzoic acid (0.228 g, 1.5 mmol) in DMF (2.5 mL) and the reaction mixture was left for 5 min. The amine **2** (0.2 g, 1.5 mmol) was added and the reaction mixture was left at room temperature overnight. The solvent was removed under reduced pressure and the crude residue diluted in ethyl acetate (15 mL), washed with water and extracted (3 times). The combined organic layers were dried over $MgSO_4$ and concentrated under vacuum. The crude was purified by column chromatography on silica gel (EtOAc/Cyclohexane, 1/9) to give the desired final product **4a**. Yellow solid. 35% yield. mp: 200.3-204.1 °C. ^{1}H NMR (300 MHz, DMSO-d_6): δ 13.14 (s, 1H), 10.37 (s, 1H), 8.24 (s, 1H), 7.87 (d, *J* = 6.9 Hz, 1H), 7.80 (dd, *J* = 7.7, 1.8 Hz, 1H), 7.56 (ddd, *J* = 8.9, 7.4, 1.8, 1.8 Hz, 1H), 7.43 – 7.19 (m, 3H), 7.12 (t, *J* = 7.5 Hz, 1H), 4.00 (s, 3H). ^{13}C NMR (101 MHz, DMSO-d_6): δ 164.1, 156.8, 140.9, 132.5, 131.4, 131.0, 130.2, 126.6, 123.8, 120.7, 115.9, 112.2, 110.3, 105.8, 56.2. IR (neat): ν_{max} 3342.3, 3121.23, 1602.27, 1245.95, cm^{-1}. HRMS (ESI$^+$): *m/z* calcd for $C_{15}H_{14}N_3O_2$ [M+H]$^+$: 268.1086, found 268.1078.

N-(1*H*-4-Indazolyl)-4-methoxybenzamide (4b).

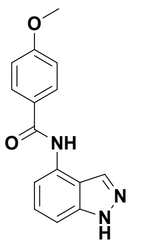

DIPEA (0.4 mL, 2.25 mmol) and the amine **2** (1.88 mmol) were stirred in DCM (5 mL) for 10 min at room temperature. 4-methoxybenzoyl chloride (0.25 mL, 1.88 mmol) was slowly added and the mixture was left at room temperature for 18 h. A precipitate fall down was filtered, washed with petroleum ether, and dried. White solid. 82% yield. mp: 234.9-236.7 °C. ^{1}H NMR (300 MHz, DMSO-*d$_6$*): δ 13.06 (s, 1H), 10.27 (s, 1H), 8.22 (s, 1H), 8.03 (d, *J* = 8.9 Hz, 2H), 7.48 (dd, *J* = 4.9, 3.4 Hz, 1H), 7.36-7.28 (m, 2H), 7.09 (d, *J* = 8.9 Hz, 2H), 3.85 (s, 3H). ^{13}C NMR (101 MHz, DMSO-*d$_6$*): δ 165.1, 161.9, 141.0, 132.9, 131.3, 129.9 (2C), 126.9, 126.2, 117.1, 113.6 (2C), 112.4, 106.1, 55.4. IR (neat): ν_{max} 3255, 3077, 1597, 1319, 1163, 580, 541, cm^{-1}. HRMS (ESI^{+}): *m/z* calcd for $C_{15}H_{14}N_3O_2$ [M+H]$^{+}$: 268.1086, found 268.1091.

N-(1*H*-4-Indazolyl)benzamide (4c).

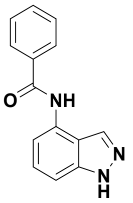

Following general procedure for preparing **4b,** the **4c** was obtained as a purple solid. 87% yield. mp: 209.2-211.5 °C. ^{1}H NMR (400 MHz, DMSO-*d$_6$*): δ 13.07 (s, 1H), 10.42 (s, 1H), 8.23 (s, 1H), 8.02 (d, *J* = 8.9 Hz, 2H), 7.78-7.40 (m, 4H), 7.34 (d, *J* = 8.9 Hz, 2H). ^{13}C NMR (101 MHz, DMSO-*d$_6$*): δ 166.3, 141.4, 135.3, 133.3, 132.0, 131.5, 128.8 (2C), 128.4 (2C), 126.7, 117.5, 112.9, 106.8. IR (neat): ν_{max} 3611, 3224, 1639, 1507, 499, cm^{-1}. HRMS (ESI^{+}): *m/z* calcd for $C_{14}H_{11}N_3O$ [M+H]$^{+}$: 238.0980, found 238.0981.

General procedure for bromination reaction. Synthesis of compounds (5a-c), (6a-c) and (7a-c): To a solution of 4-substituted-1*H*-indazoles **3** or **4** (1 equiv.) dissolved in DMF (3 mL) was added 1.1 equivalent of NBS. The reaction mixture was stirred at 80 °C for 18 h. After cooling, the reaction mixture was dissolved in ethyl acetate, washed with an aqueous solution of thiosulphate, with water, dried over $MgSO_4$, filtered and evaporated. The resulting residue was purified by column chromatography on silica gel (eluted with EtOAc/Cyclohexane, 2/8).

N-(7-Bromo-1*H*-indazol-4-yl)-4-methylbenzenesulfonamide (5a).

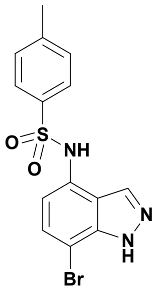

It was obtained as a purple solid. 84% yield. mp: 215.5-217.6 °C. ^{1}H NMR (300 MHz, DMSO-*d$_6$*): δ 13.48 (s, 1H), 10.67 (s, 1H), 8.35 (s, 1H), 7.67 (d, *J* = 8.3 Hz, 2H), 7.43 (d, *J* = 8.1 Hz, 1H), 7.31 (d, *J* = 8.0 Hz, 2H), 6.87 (d, *J* = 8.1 Hz, 1H), 2.29 (s, 3H). ^{13}C NMR (75 MHz, DMSO-*d$_6$*): δ 143.4, 139.6, 136.4, 133.2, 129.6 (2C), 129, 126.6 (2C), 118, 111.8, 97.4 (CBr), 20.9. IR (neat): ν_{max} 3393, 3241, 1594, 1331, 1161, 541, cm^{-1}. HRMS (ESI^{-}): *m/z* calcd for $C_{14}H_{11}BrN_3O_2S$ [M-H]$^{-}$: 363.9755, found 363.9760.

N-(7-Bromo-1*H*-indazol-4-yl)-4-methoxybenzenesulfonamide (5b).

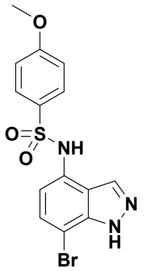

It was obtained as a white solid. 77% yield mp: 208.0-210.2 °C. ^{1}H NMR (300 MHz, DMSO-d_6): δ 13.47 (s, 1H), 10.58 (s, 1H), 8.35 (s, 1H), 7.72 (d, *J* = 8.3 Hz, 2H), 7.43 (d, *J* = 7.8 Hz, 1H), 7.02 (d, *J* = 8.3 Hz, 2H), 6.68 (d, *J* = 7.8 Hz, 1H), 3.75 (s, 3H). ^{13}C NMR (75 MHz, DMSO-d_6): δ 162.5, 139.7, 133.2, 130.9, 129.9, 128.9 (2C), 128.8, 118.0, 114.3 (2C), 111.8, 97.3 (CBr), 55.5. IR (neat): ν_{max} 3264, 3081, 1599, 1260, 1325, 1158, 546, cm^{-1}. HRMS (ESI$^-$): *m/z* calcd for $C_{14}H_{12}BrN_3O_2S$ [M-H]$^-$: 379.9704, found 379.9707.

N-(7-Bromo-1*H*-indazol-4-yl)-4-nitrobenzenesulfonamide (5c).

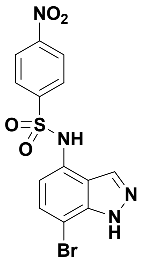

It was obtained as a yellow solid. 17% yield. mp: 239.0-243.2 °C. ^{1}H NMR (300 MHz, DMSO-d_6): δ 13.55 (s, 1H), 11.03 (s, 1H), 8.34 – 8.29 (m, 3H), 8.01 (d, *J* = 8.8 Hz, 2H), 7.46 (d, *J* = 8.0 Hz, 1H), 6.87 (d, *J* = 8.0 Hz, 1H). ^{13}C NMR (101 MHz, DMSO): δ 149.8, 144.7, 139.8, 133.1, 129.0, 128.7, 128.4 (2C), 124.5 (2C), 118.6, 113.3, 98.6 (CBr). IR (neat): ν_{max} 3436, 3041, 1604, 1341, 1167, 1521, 1310, 594, cm^{-1}. HRMS (ESI$^-$): *m/z* calcd for $C_{13}H_8BrN_4O_4S$ [M-H]$^-$: 394.9450, found 3394.9448.

N-(5,7-Dibromo-1*H*-indazol-4-yl)-4-methylbenzenesulfonamide (6a).

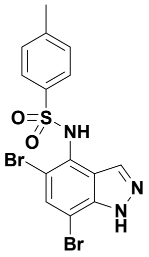

It was obtained as a white solid. 10% yield. mp: 188.0-189.1 °C. ^{1}H NMR (300 MHz, DMSO-d_6): δ 13.72 (s, 1H), 10.13 (s, 1H), 7.78 (s, 1H), 7.66 (d, *J* = 1.5 Hz, 1H), 7.51 (d, *J* = 8.3 Hz, 2H), 7.33 (d, *J* = 8.0 Hz, 2H), 2.37 (s, 3H). ^{13}C NMR (101 MHz, DMSO-d_6): δ 143.3, 139.2, 137.6, 133.9, 131.8, 129.9, 126.9 (2C), 126.8 (2C), 123.9, 113.7 (C-Br), 102.2 (C-Br), 21.0. IR (neat): ν_{max} 3255, 3077, 1597, 1319, 1163, 580, 541, cm^{-1}. HRMS (ESI$^+$): *m/z* calcd for $C_{14}H_{12}Br_2N_3O_2S$ [M+H]$^+$: 443.9017, found 443.9007.

N-(5,7-Dibromo-1*H*-indazol-4-yl)-4-methoxybenzenesulfonamide (6b).

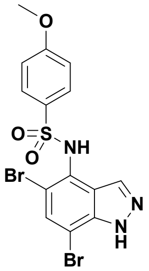

It was obtained as a white solid. 15% yield. mp: 208.1-211.2 °C. ^{1}H NMR (300 MHz, DMSO-d_6): δ 13.71 (s, 1H), 10.04 (s, 1H), 7.79 (s, 1H), 7.69 (d, *J* = 1.5 Hz, 1H), 7.55 (d, *J* = 8.9 Hz, 1H), 7.05 (d, *J* = 9 Hz, 1H), 3.82 (s, 3H). ^{13}C NMR (101 MHz, DMSO-d_6): δ 162.6, 139.2, 134.0, 132.07 131.8, 129.0 (2C), 127.0, 123.8, 114.29 (2C), 113.6 (CBr), 102.1 (CBr), 55.6. IR (neat): $ν_{max}$ 3264, 3081, 1599, 1262, 1325, 1158, 586.26, 546, cm^{-1}. HRMS (ESI^{-}): *m/z* calcd for $C_{14}H_{10}Br_2N_3O_3S$ [M-H]$^{-}$: 457.8810, found 457.8804.

N-(5,7-Dibromo-1*H*-indazol-4-yl)-4-nitrobenzenesulfonamide (6c).

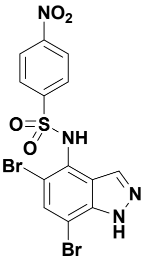

It was obtained as a brown solid. 20% yield. mp: 248.2-250.4 °C. ^{1}H NMR (300 MHz, DMSO-d_6): δ 13.81 (s, 1H), 10.72 (s, 1H), 8.37 (d, *J* = 8.9 Hz, 2H), 7.90 (d, *J* = 8.9 Hz, 2H), 7.83 (s, 1H), 7.79 (s, 1H). ^{13}C NMR (101 MHz, DMSO-d_6): δ 149.8, 145.88, 139.3, 133.8, 131.8, 128.4 (2C), 126.1, 124.5 (2C), 124.0, 113.7 (CBr), 102.9 (CBr). IR (neat): $ν_{max}$ 3435, 3039, 1625, 1342, 1166, 1520, 1310, 595, 537, cm^{-1}. HRMS (ESI^{-}): *m/z* calcd for $C_{13}H_7Br_2N_4O_4S$ [M-H]$^{-}$: 472.8555, found 472.8549.

N-(4-Bromo-1*H*-indazol-7-yl)-4-methoxybenzamide (7a).

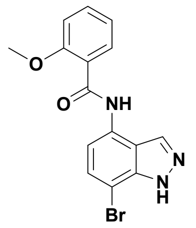

It was obtained as a white solid. 82% yield. mp: 262.4-264.9 °C. ^{1}H NMR (300MHz, DMSO-d_6): δ 13.45 (s, 1H), 10.34 (s, 1H), 8.37 (s, 1H), 8.01 (d, *J* = 8.8 Hz, 2H), 7.55 (d, *J* = 8.1 Hz, 1H), 7.48 (d, *J* = 8.1 Hz, 1H), 7.09 (d, *J* = 8.8 Hz, 2H), 3.86 (s, 3H). ^{13}C NMR (101 MHz, DMSO-d_6): δ 166.2, 162.1, 139.7, 134.3, 131.1, 129.9 (2C), 128.8, 126.6, 118.4, 113.7, 113.6 (2C), 97.0 (CBr), 55.4. IR (neat): $ν_{max}$ 3343, 3244, 1588, 1648, 1236, 591, cm^{-1}. HRMS (ESI^{-}): *m/z* calcd for $C_{15}H_{11}N_3O_2Br$ [M-H]$^{-}$: 344.0035 found 344.0033.

N-(4-Bromo-1*H*-indazol-7-yl)-4-methoxybenzamide (7b).

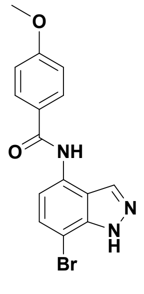

It was obtained as a yellow solid. 56% yield. mp: 215-217.2 °C. ^{1}H NMR (300 MHz, DMSO-d_6): δ 13.55 (s, 1H), 10.44 (s, 1H), 8.40 (s, 1H), 7.85 (d, *J* = 8.1 Hz, 1H), 7.76 (dd, *J* = 7.6, 1.7 Hz, 1H), 7.59 – 7.52 (m, 2H), 7.24 (d, *J* = 8.3 Hz, 1H), 7.14 – 7.08 (m, 1H), 3.98 (s, 3H). ^{13}C NMR (101 MHz, DMSO-d_6): δ 164.4, 156.8, 139.7, 132.9, 132.6, 130.8, 130.2, 129.1, 123.9, 120.6, 117.2, 112.2, 111.8, 96.5 (CBr), 56.01. IR (neat): $ν_{max}$ 3343, 3244, 1588, 1648, 1236, 591, cm^{-1}. HRMS (ESI^{-}): *m/z* calcd for $C_{15}H_{11}$ Br N_3O_2 [M-H]$^{-}$: 344.0035 found 344.0026.

N-(4-Bromo-1*H*-indazol-7-yl)benzamide (7c).

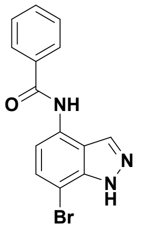

It was obtained as a white solid. 89% yield. mp: 222-225.2 °C. ^{1}H NMR (400 MHz, DMSO-d_6): δ 13.48 (s, 1H), 10.52 (s, 1H), 8.42 (s, 1H), 8.02 (d, *J* = 7.1 Hz, 2H), 7.62 (d, *J* = 7.2, 1.7 Hz, 1H), 7.59 – 7.50 (m, 4H). ^{13}C NMR (101 MHz, DMSO-d_6): δ 166.4, 140.2, 135.1, 134.8, 134.2, 132.2, 131.4, 129.3, 128.8 (2C), 128.4 (2C), 118.8, 97.8 (CBr). IR (neat): ν $_{max}$ 3611, 3224, 1639, 1507, 499, cm^{-1}. HRMS (ESI^{+}): *m/z* calcd for $C_{14}H_{10}BrN_3O_3$ [M+H]$^{+}$: 316.0085, found 316.0084.

General procedure for Suzuki reaction. Synthesis of compounds (8aa-af), (8ba-be), (8cb), (9ab), (9ba-bb and 9bd-bf) and (9ca and 9cg): To a solution of 7-bromo-4-substituted *NH*-indazoles **5** or **7** dissolved in 1.5 mL of a mixture of dioxane/EtOH/H_2O (3/1/0.5, v/v/v) in a vial microwave tube with a stir bar, were added arylboronic acid (1.5 equiv.), cesium carbonate (1.3 equiv. in 0.5 mL of H_2O) and $Pd(PPh_3)_4$ (0.1 equiv.) under argon. The reaction vessel was sealed with a silicon septum and stirred at 140°C for 4 h under conventional heating or for 2 h under microwave irradiation. The reaction mixture was then allowed to cool to room temperature, diluted with ethyl acetate (15 mL) and water (10 mL), and extracted (3 times). The combined organic layer was dried over $MgSO_4$ and concentrated under vacuum. The crude material was purified by column chromatography on silica gel (EtOAc/Ether, 1/9) to give the desired final product.

N-(7-(4-Butylphenyl)-1*H*-indazol-4-yl)-4-methylbenzenesulfonamide (8aa).

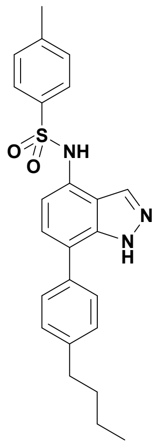

It was obtained as a white solid. 62% yield. mp: 102.0-104.0 °C. ^{1}H NMR (400 MHz, DMSO-d_6): δ 13.11 (s, 1H), 10.54 (s, 1H), 8.30 (s, 1H), 7.72 (d, *J* = 8.3 Hz, 2H), 7.51 (d, *J* = 7.7 Hz, 2H), 7.33-7.29 (m, 4H), 7.24 (d, *J* = 7.8 Hz, 1H), 7.00 (d, *J* = 7.8 Hz, 1H), 2.64 (t, *J* = 7.6 Hz, 2H), 2.32 (s, 3H), 1.60 (ddt, *J* = 9.0, 7.7, 3.6 Hz, 2H), 1.35 (dq, *J* = 14.5, 7.3 Hz, 2H), 0.92 (t, *J* = 9.2 Hz, 3H). ^{13}C NMR (101 MHz, DMSO-d_6): δ 143.6, 141.9, 138.5, 136.5, 134.0, 132.2, 129.6 (2C), 128.9 (2C), 128.8, 127.6 (2C), 126.7 (2C), 125.9, 120.7, 117.8, 111.9, 34.4, 32.9, 21.6, 20.8, 13.6. IR (neat): $ν_{max}$ 3133, 3072, 1598, 1337, 1154, cm^{-1}. HRMS (ESI^{-}): *m/z* calcd for $C_{24}H_{24}N_3O_2S$ [M-H]$^{-}$: 418.1589, found 418.1589.

N-(7-(4-Methoxyphenyl)-1*H*-indazol-4-yl)-4-methylbenzenesulfonamide (8ab).

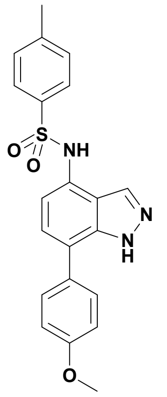

It was obtained as a yellow paste. 70% yield. ^{1}H NMR (300 MHz, DMSO-d_6): δ 13.13 (s, 1H), 10.56 (s, 1H), 8.30 (s, 1H), 7.72 (d, *J* = 8.3 Hz, 2H), 7.53 (d, *J* = 8.6 Hz, 2H), 7.32 (d, *J* = 8.1 Hz, 2H), 7.20 (d, *J* = 7.8 Hz, 1H), 7.04 (d, *J* = 8.7 Hz, 2H), 6.99 (d, *J* = 7.8 Hz, 1H), 3.81 (s, 3H), 2.30 (s, 3H). ^{13}C NMR (75 MHz, DMSO-d_6): δ 158.7, 143.5, 138.5, 136.6, 132.4, 129.6 (2C), 129.1 (2C), 128.9, 128.5, 126.7 (2C), 125.6, 120.5, 117.8, 114.4 (2C), 111.9, 55.1, 20.8. IR (neat): ν_{max} 3342, 3121, 1602, 1332, 1156, 1245, cm^{-1}. HRMS (ESI$^+$): *m/z* calcd for $C_{21}H_{19}N_3O_3S$ [M+H]$^+$: 394.1225, found 394.1218.

N-(7-(2-Methoxyphenyl)-1*H*-indazol-4-yl)-4-methylbenzenesulfonamide (8ac).

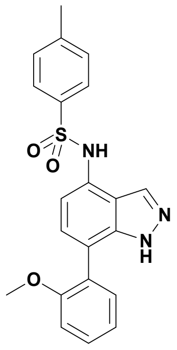

It was obtained as a white solid. 76% yield. mp: 123.2-124.3 °C. ^{1}H NMR (400 MHz, DMSO-d_6): δ 12.65 (s, 1H), 10.55 (s, 1H), 8.23 (d, *J* = 1.4 Hz, 1H), 7.76 (d, *J* = 8.3 Hz, 2H), 7.39 (ddd, *J* = 8.3, 7.5, 1.7 Hz, 1H), 7.34 (d, *J* = 8.0 Hz, 2H), 7.25 (dd, *J* = 7.5, 1.7 Hz, 1H), 7.12 – 7.09 (m, 1H), 7.08 (d, *J* = 7.7 Hz, 1H), 7.02 (td, *J* = 7.4, 1.0 Hz, 1H), 6.96 (d, *J* = 7.7 Hz, 1H), 3.70 (s, 3H), 2.32 (s, 3H). ^{13}C NMR (75 MHz, DMSO-d_6): δ 156.4, 143.4, 139.0, 136.9, 131.6, 130.8, 129.7 (2C), 129.3, 129.0, 127.1, 126.7 (2C), 125.7, 120.5, 118.2, 116.8, 111.2, 110.6, 54.9, 20.9. IR (neat): ν_{max} 3357, 3133, 1595, 1339, 1154, 1239, cm^{-1}. HRMS (ESI$^-$): *m/z* calcd for $C_{21}H_{17}N_3O_3S$ [M-H]$^-$: 392.1069, found 392.1062.

4-Methyl-N-(7-(4-nitrophenyl)-1*H*-indazol-4-yl)benzenesulfonamide (8ad).

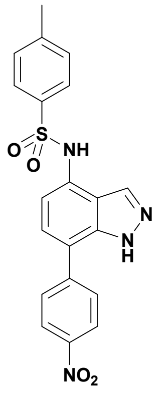

It was obtained as a yellow Solid. 78% yield. mp: 222.8-224.2 °C. ^{1}H NMR (400 MHz, DMSO-d_6): δ 13.35 (s, 1H), 10.78 (s, 1H), 8.42 (s, 1H), 8.31 (d, *J* = 8.7 Hz, 2H), 7.89 (d, *J* = 8.7 Hz, 2H), 7.76 (d, *J* = 8.3 Hz, 2H), 7.42 (d, *J* = 7.9 Hz, 1H), 7.34 (d, *J* = 8.1 Hz,

2H), 7.09 (d, J = 7.9 Hz, 1H), 2.31 (s, 3H). ^{13}C NMR (101 MHz, DMSO-d_6): δ 146.3, 143.7, 143.5, 138.4, 136.9, 132.8, 131.3, 129.7 (2C), 129.0 (2C), 127.3, 126.7 (2C), 124.0 (2C), 117.7, 117.5, 110.3, 20.9. IR (neat): ν_{max} 3393, 3158, 1592, 1338, 1150, 1515, 1338, cm^{-1}. HRMS (ESI$^-$): *m/z* calcd for $C_{20}H_{15}N_4O_4S$ [M-H]$^-$: 407.0814, found 407.0809.

N-(7-(Furan-2-yl)-1*H*-indazol-4-yl)-4-methylbenzenesulfonamide (8ae).

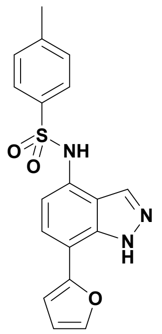

It was obtained as a white solid. 75% yield. mp: 210.3-211.4 °C. ^{1}H NMR (400 MHz, DMSO-d_6): δ 13.17 (s, 1H), 10.61 (s, 1H), 8.35 (s, 1H), 7.76 (d, J = 1.4 Hz, 1H), 7.69 (d, J = 8.4 Hz, 2H), 7.57 (d, J = 7.9 Hz, 1H), 7.30 (d, J = 8.0 Hz, 2H), 7.09 (d, J = 3.1 Hz, 1H), 7.03 (d, J = 8.0 Hz, 1H), 6.65 (dd, J = 3.4, 1.8 Hz, 1H), 2.30 (s, 3H). ^{13}C NMR (101 MHz, DMSO-d_6): δ 150.2, 143.3, 142.6, 136.7, 135.8, 132.6, 129.6 (2C), 129.5, 126.7 (2C), 122.1, 117.9, 112.0, 111.0, 110.2, 106.6, 20.88. IR (neat): ν_{max} 3359, 3133, 1616, 1338, 1155, 1289 cm^{-1}. HRMS (ESI$^-$): *m/z* calcd for $C_{18}H_{14}N_3O_3S$ [M-H]$^-$: 352.0756, found 352.0754.

4-Methyl-N-(7-(thiophen-2-yl)-1*H*-indazol-4-yl)benzenesulfonamide (8af).

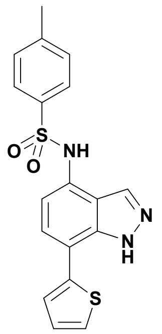

It was obtained as a white solid. 80% yield. mp: 188.7-190.1 °C. ^{1}H NMR (300 MHz, DMSO-d_6): δ 13.23 (s, 1H), 10.67 (s, 1H), 8.36 (s, 1H), 7.71 (d, J = 8.3 Hz, 2H), 7.59 (d, J = 5.0 Hz, 1H), 7.55 (d, J = 3.1 Hz, 1H), 7.36 (d, J = 6.3 Hz, 1H), 7.31 (d, J = 8.1 Hz, 2H), 7.22 – 7.17 (m, 1H), 7.01 (d, J = 7.8 Hz, 1H), 2.29 (s, 3H). ^{13}C NMR (75 MHz, DMSO-d_6): δ 143.4, 139.0, 137.5, 136.7, 132.8, 129.7 (2C), 129.6, 128.4, 126.7 (2C), 125.5, 125.2, 117.7, 113.6, 110.9, 20.9. IR (neat): ν_{max} 3347, 3122, 1596, 1331, 1154, cm^{-1}. HRMS (ESI$^+$): *m/z* calcd for $C_{18}H_{16}N_3O_2S_2$ [M+H]$^+$: 370.0684, found 370.0675.

N-(7-(4-Butylphenyl)-1*H*-indazol-4-yl)-4-methoxybenzenesulfonamide (8ba).

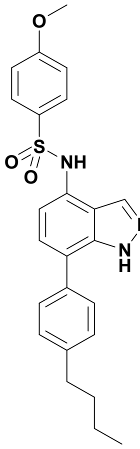

It was obtained as a white solid. 57% yield. mp: 172.8-174.1 °C. ^{1}H NMR (300 MHz, DMSO-d_6): δ 13.14 (s, 1H), 10.51 (s, 1H), 8.32 (s, 1H), 7.77 (d, J = 8.9 Hz, 2H), 7.51 (d, J = 8.1 Hz, 2H), 7.30 (d, J = 8.1 Hz, 2H), 7.24 (d, J = 7.8 Hz, 1H), 7.07 – 6.99 (m, 3H), 3.76 (s, 3H), 2.63 (t, J = 7.6 Hz, 2H), 1.65 – 1.54 (m, 2H), 1.34 (dq, J = 14.4, 7.3 Hz, 2H), 0.92 (t, J = 7.3 Hz, 3H). ^{13}C NMR (75 MHz, DMSO-d_6): δ 162.4, 141.6, 138.5, 134.4, 132.5, 131.3, 129.2, 128.9 (2C), 128.8 (2C), 127.7 (2C), 125.8, 120.3, 117.6, 114.3 (2C), 111.1, 55.2, 34.5, 33.0, 21.7, 13.8. IR (neat): ν_{max} 3344, 3222, 1596, 1333, 1150, 1261 cm^{-1}. HRMS (ESI$^+$): m/z calcd for $C_{24}H_{26}N_3O_3S$ [M+H]$^+$: 436.1695, found 436.1686.

4-Methoxy-N-(7-(4-methoxyphenyl)-1*H*-indazol-4-yl)benzenesulfonamide (8bb).

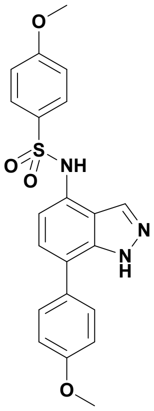

It was obtained as a white solid. 81% yield. mp: 105.1-109.8 °C. ^{1}H NMR (300 MHz, DMSO-d_6): δ 13.13 (s, 1H), 10.48 (s, 1H), 8.30 (s, 1H), 7.76 (d, J = 8.4 Hz, 2H), 7.53 (d, J = 8.7 Hz, 2H), 7.20 (d, J = 7.8 Hz, 1H), 7.04 (dd, J = 8.8, 1.6 Hz, 4H), 7.00 (d, J = 7.8 Hz, 1H), 3.81 (s, 3H), 3.77 (s, 3H). ^{13}C NMR (75 MHz, DMSO-d_6): δ 162.4, 158.7, 138.5, 132.5, 131.3, 130.0, 129.0 (2C), 128.9 (2C), 125.5, 120.3, 117.8, 114.4 (2C), 111.6, 55.5, 55.1. IR (neat): ν_{max} 3251, 2928, 1595, 1331, 1180, 1148 cm^{-1}. HRMS (ESI$^+$): m/z calcd for $C_{21}H_{20}N_3O_4S$ [M+H]$^+$: 410.1175, found 410.1164.

4-Methoxy-N-(7-(2-methoxyphenyl)-1*H*-indazol-4-yl)benzenesulfonamide (8bc).

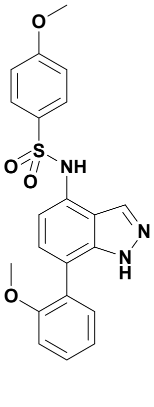

It was obtained as a white solid. 75% yield. mp: 108.8-111.0 °C. ^{1}H NMR (300 MHz, DMSO-d_6): δ 12.67 (s, 1H), 10.45 (s, 1H), 8.25 (s, 1H), 7.81 (d, *J* = 8.4 Hz, 2H), 7.39 (ddd, *J* = 8.3, 7.3, 1.8 Hz, 1H), 7.25 (dd, *J* = 7.5, 1.8 Hz, 1H), 7.07 (m, 5H), 3.77 (s, 3H), 3.70 (s, 3H). ^{13}C NMR (75 MHz, DMSO-d_6): δ 170.3, 162.4, 156.5, 139.0, 131.6, 130.8, 129.4, 129.2, 128.9 (2C), 127.1, 125.9, 120.4, 117.9, 116.8, 114.4 (4C), 111.2, 110.2, 55.6, 55.0. IR (neat): ν_{max} 3358, 3071, 1594, 1340, 1150, 1239 cm^{-1}. HRMS (ESI$^+$): *m/z* calcd for $C_{21}H_{20}N_3O_4S$ [M+H]$^+$: 410.1175, found 410.1165.

4-Methoxy-N-(7-(4-nitrophenyl)-1*H*-indazol-4-yl)benzenesulfonamide (8bd).

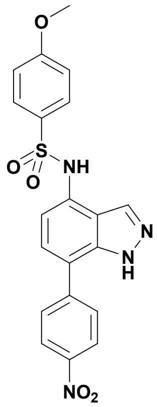

It was obtained as a yellow solid. 75% yield. mp: 100.2-103.2 °C. ^{1}H NMR (300 MHz, DMSO-d_6): δ 13.37 (s, 1H), 10.75 (s, 1H), 8.43 (s, 1H), 8.31 (d, *J* = 8.8 Hz, 2H), 7.89 (d, *J* = 8.6 Hz, 2H), 7.80 (d, *J* = 8.9 Hz, 2H), 7.43 (d, *J* = 7.8 Hz, 1H), 7.31 – 7.24 (m, 1H), 7.19 (d, *J* = 7.2 Hz, 1H), 7.08 (dd, *J* = 11.6, 8.5 Hz, 3H), 3.77 (s, 3H). ^{13}C NMR (75 MHz, DMSO-d_6): δ 162.5, 146.2, 143.9, 140.9, 131.3, 130.0, 128.9 (4C), 127.3, 126.5, 123.9 (2C), 116.8, 114.4 (2C), 114.3 (2C), 110.3, 106.1, 55.6. IR (neat): ν_{max} 3393, 3158, 1592, 1338, 1150, 1515, 1338, 1274, cm^{-1}. HRMS (ESI$^-$): *m/z* calcd for $C_{20}H_{15}N_4O_5S$ [M-H]$^-$: 423.0763, found 423.0758.

N-(7-(Furan-2-yl)-1*H*-indazol-4-yl)-4-methoxybenzenesulfonamide (8be).

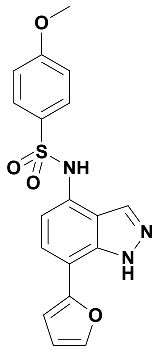

It was obtained as a white Solid. 72% yield. mp: 227.0-230.1 °C. ^{1}H NMR (300 MHz, DMSO-d_6): δ 13.19 (s, 1H), 10.57 (s, 1H), 8.34 (s, 1H), 7.76 (d, *J* = 1.4 Hz, 1H), 7.73 (d, *J* = 8.9 Hz, 2H), 7.58 (d, *J* = 8.0 Hz, 1H), 7.09 (d, *J* = 3.3 Hz, 1H), 7.02 (m, 3H), 6.65 (dd, *J* = 3.4, 1.8 Hz, 1H), 3.75 (s, 3H). ^{13}C NMR (101 MHz, DMSO-d_6): δ 162.5, 150.2, 142.7, 132.1, 130.6, 129.1, 128.9 (2C), 122.2, 118.1, 114.5, 114.2 (2C), 112.2, 112.1, 110.7, 106.5, 55.5. IR (neat): ν_{max} 3222, 3154 cm^{-1}.HRMS (ESI$^-$): *m/z* calcd for $C_{18}H_{14}N_3O_4S$ [M-H]$^-$: 368.0705, found 368.0699.

N-(7-(4-Methoxyphenyl)-1*H*-indazol-4-yl)-4-nitrobenzenesulfonamide (8cb).

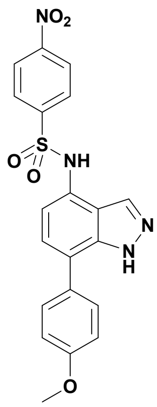

It was obtained as a white solid. 71% yield. mp: 99.9-102 °C. ^{1}H NMR (300 MHz, DMSO-d_6): δ 13.20 (s, 1H), 10.94 (s, 1H), 8.36 (d, J = 8.9 Hz, 2H), 8.23 (s, 1H), 8.06 (d, J = 8.9 Hz, 2H), 7.54 (d, J = 8.4 Hz, 2H), 7.23 (d, J = 7.8 Hz, 1H), 7.05 (d, J = 8.7 Hz, 2H), 6.99 (d, J = 7.8 Hz, 1H), 3.81 (s, 3H). ^{13}C NMR (101 MHz, DMSO-d_6): δ 158.8, 149.8, 145.2, 138.6, 132.3, 129.2, 129.1 (2C), 128.3 (2C), 127.6, 125.5, 124.6 (2C), 121.3, 118.3, 114.4 (2C), 112.9, 55.2. IR (neat): ν_{max} 3105, 2922, 1606, 1310, 1162, 1504, 1345, 1246 cm^{-1}. HRMS (ESI$^-$): *m/z* calcd for $C_{20}H_{15}N_4O_5S$ [M-H]$^-$: 423.0763, found 423.0764.

2-Methoxy-N-(7-(4-methoxyphenyl)-1*H*-indazol-4-yl)benzamide (9ab).

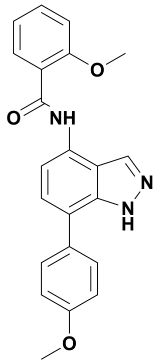

It was obtained as a yellow solid. 78% yield. mp: 228.3-231.1 °C . ^{1}H NMR (300 MHz, DMSO-d_6): δ 13.24 (s, 1H), 10.41 (s, 1H), 8.34 (s, 1H), 7.98 (d, J = 7.8 Hz, 1H), 7.83 (dd, J = 7.6, 1.6 Hz, 1H), 7.63 (d, J = 8.3 Hz, 2H), 7.60 – 7.53 (m, 1H), 7.35 (d, J = 7.9 Hz, 1H), 7.26 (d, J = 8.3 Hz, 1H), 7.14 (t, J = 5.5 Hz, 1H), 7.09 (d, J = 8.8 Hz, 2H), 4.03 (s, 3H), 3.84 (s, 3H). ^{13}C NMR (101 MHz, DMSO-d_6): δ 164.1, 158.7, 156.9, 138.5, 132.6, 131.9, 130.3, 129.9, 129.8, 129.1 (2C), 125.7, 123.8, 120.7, 119.9, 116.8, 114.4 (2C), 112.2, 111.3, 56.2, 55.2. IR (neat): ν_{max} 3341, 3250, 1600, 1661 cm^{-1}. HRMS (ESI$^-$): *m/z* calcd for $C_{22}H_{18}N_3O_3$ [M-H]$^-$: 372.1348, found 372.1350.

N-(7-(4-propylphenyl)-1*H*-indazol-4-yl))-4-methoxybenzamideb(9ba).

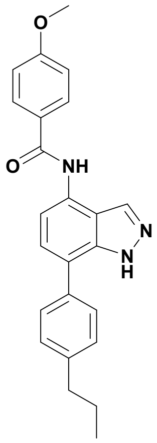

It was obtained as a white solid. 70% yield. mp: 214-216 °C. ^{1}H NMR (250MHz, DMSO-d_6): δ 13.12 (s, 1H), 10.29 (s, 1H), 8.34 (s, 1H), 8.05 (d, *J* = 8.8 Hz, 2H), 7.62 (d, *J* = 7.8 Hz, 2H), 7.37 (t, *J* = 7.8 Hz, 4H), 7.11 (d, *J* = 8.9 Hz, 2H), 3.87 (s, 3H), 2.65 (d, *J* = 8.5 Hz, 2H), 1.68 (dd, *J* = 7.5, 1.3 Hz, 2H), 0.97 (t, *J* = 7.3 Hz, 3H). ^{13}C NMR (63 MHz, DMSO-d_6): δ 166.4, 130.4 (2C), 130.0, 129.4 (2C), 128.5, 128.5, 128.3 (2C), 126.1, 114.0 (2C), 113.7, 55.9, 37.5, 24.4, 14.20. IR (neat): ν_{max} 3251, 2926, 1644, 1606 cm^{-1}. HRMS (ASAP$^+$): *m/z* calcd for $C_{24}H_{23}N_3O_2$ [M+H]$^+$: 386.1868, found 386.1867.

4-Methoxy-N-(7-(4-methoxyphenyl)-1*H*-indazol-4-yl)benzamide (9bb).

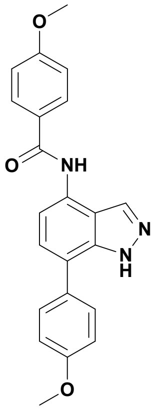

It was obtained as a yellow solid. 82% yield. mp: 268.2-270.2 °C . ^{1}H NMR (300 MHz, DMSO-d_6): δ 13.13 (s, 1H), 10.31 (s, 1H), 8.32 (s, 1H), 8.05 (d, *J* = 8.8 Hz, 2H), 7.65 - 7.58 (m, 3H), 7.34 (d, *J* = 7.8 Hz, 1H), 7.10 (dd, *J* = 8.9, 1.9 Hz, 4H), 3.86 (s, 3H), 3.84 (s, 3H). ^{13}C NMR (101 MHz, DMSO-d_6): δ 179.3, 165.2, 162.0, 158.7, 130.2, 129.9 (2C), 129.8 (2C), 129.1 (2C), 126.8, 125.3, 117.9, 114.40 (2C), 113.5 (2C), 113.3, 55.4, 55.2. IR (neat): ν_{max} 3256.21, 2928.98, 606.17, 1636.45 cm^{-1}. HRMS (ASAP$^+$): *m/z* calcd for $C_{22}H_{20}N_3O_3$ [M+H]$^+$: 374.1505, found 374.1493.

4-Methoxy-N-(7-(4-nitrophenyl)-1H-indazol-4-yl)benzamide (9bd).

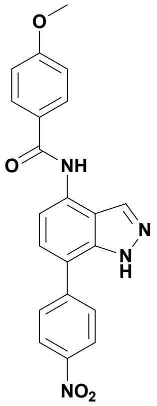

It was obtained as a yellow solid. 76% yield. mp: 254.6-258.4 °C . ^{1}H NMR (300MHz, DMSO-d_6): δ 13.37 (s, 1H), 10.42 (s, 1H), 8.42 (s, 1H), 8.36 (d, *J* = 8.8 Hz, 2H), 8.04 (d, *J* = 8.9 Hz, 2H), 7.99 (d, *J* = 8.7 Hz, 2H), 7.73 (d, *J* = 7.9 Hz, 1H), 7.56 (d, *J* = 7.9 Hz, 1H), 7.11 (d, *J* = 8.9 Hz, 2H), 3.87 (s, 3H). ^{13}C NMR (101 MHz, DMSO-d_6): δ 165.4, 162.1, 144.1, 130.0 (2C), 129.0 (2C), 127.0, 126.6, 124.0 (2C), 117.8, 113.6 (2C), 113.0, 55.4. IR (neat): ν_{max} 3243.98, 2918.27, 1603.15, 1643.65 cm^{-1}. HRMS (ASAP$^-$): *m/z* calcd for $C_{21}H_{15}N_4O_4$ [M-H]$^-$: 387.1093, found 387.1099.

N-(7-(Furan-2-yl)-1*H*-indazol-4-yl)-4-methoxybenzamide (9be).

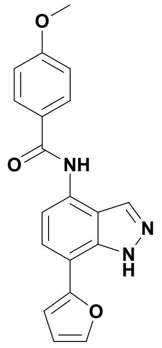

It was obtained as a yellow solid. 91% yield. mp: 248.3-251.4 °C. ^{1}H NMR (300MHz, DMSO-d_6): δ 13.19 (s, 1H), 10.35 (s, 1H), 8.39 (s, 1H), 8.03 (d, *J* = 8.9 Hz, 2H), 7.81 (d, *J* = 1.6 Hz, 1H), 7.73 (d, *J* = 8.0 Hz, 1H), 7.64 (d, *J* = 8.0 Hz, 1H), 7.18 (d, *J* = 3.4 Hz, 1H), 7.10 (d, *J* = 8.9 Hz, 2H), 6.70 (dd, *J* = 3.4, 1.8 Hz, 1H), 3.86 (s, 3H). ^{13}C NMR (101 MHz, DMSO-d_6): δ 165.3, 162.0, 150.6, 142.4, 136.1, 133.2, 130.8, 129.9 (2C), 126.6, 121.9, 118.0, 113.6 (2C), 112.9, 112.0, 110.3, 106.4, 55.4. IR (neat): ν_{max} 3253.77, 2926.60, 1605.62, 1638.15 cm^{-1}. HRMS (ASAP$^+$): *m/z* calcd for $C_{19}H_{16}N_3O_3$ [M+H]$^+$: 334.1192, found 334.1180.

N-(7-(thiophen-2-yl)-1*H*-indazol-4-yl)-4-methoxybenzamide (9bf).

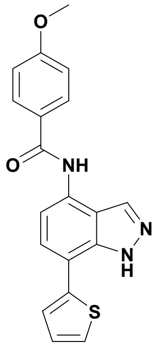

It was obtained as a white solid. 85% yield. mp: 205-207. ^{1}H NMR (250MHz, DMSO-d_6): δ 13.22 (s, 1H), 10.35 (s, 1H), 8.42 (s, 1H), 8.06 (d, *J* = 8.8 Hz, 2H), 7.87 – 7.37 (m, 4H), 7.25 (dd, *J* = 5.1, 3.6 Hz, 1H), 7.10 (d, *J* = 8.8 Hz, 2H), 3.87 (s, 3H). ^{13}C NMR (63 MHz, DMSO-d_6): δ 132.5, 132.0, 131.8, 130.4 (2C), 130.3, 129.3, 129.1, 128.8, 127.6, 126.8, 126.5, 125.7, 125.6, 114.1, 114.0 (2C), 113.5, 55.9. IR (neat): ν_{max} 3248, 2926, 2867, 1606, 1577 cm^{-1}. HRMS (ASAP$^+$): *m/z* calcd for $C_{19}H_{15}N_3O_2S$ [M+H]$^+$: 350.0963, found 350.0955.

N-(7-(4-propylphenyl)-1*H*-indazol-4-yl)benzamide (9ca).

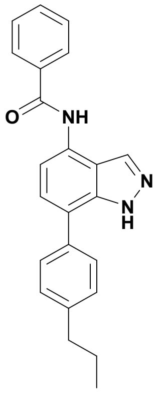

It was obtained as a yellow solid. 82% yield. mp: 196-198 °C. ^{1}H NMR (400MHz, DMSO-d_6): δ 13.17 (s, 1H), 10.48 (s, 1H), 8.37 (s, 1H), 8.06 (d, *J* = 8.8 Hz, 2H), 7.76 – 7.50 (m, 6H), 7.40 (d, *J* = 7.7 Hz, 1H), 7.35 (d, *J* = 8.8 Hz, 2H), 3.38 (s, 3H), 2.64 (t, *J* = 7.6 Hz, 2H), 1.67 (q, *J* = 7.4 Hz, 2H), 0.96 (t, *J* = 7.3 Hz, 3H). ^{13}C NMR (101 MHz, DMSO-d_6): δ 166.4, 141.8, 139.0, 135.3, 133.9,

132.1, 130.8, 129.4 (2C), 128.8 (2C), 128.4 (2C), 128.3 (2C), 128.1, 126.1, 121.1, 118.4, 113.8, 37.4, 24.5, 14.2. IR (neat): ν_{max} 3250, 2957, 2923, 1647, 1615 cm^{-1}. HRMS (ASAP+): *m/z* calcd for $C_{23}H_{21}N_3O$ $[M+H]^+$: 356.1763, found 356.1768.

N-(7-(p-tolyl)-1*H*-indazol-4-yl)benzamide (9cb).

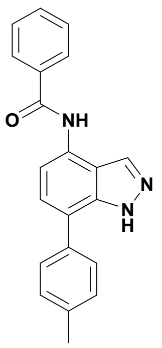

It was obtained as a yellow solid. 75% yield. mp: 202-204 °C. ^1H NMR (400MHz, DMSO-d_6): δ 13.15 (s, 1H), 10.47 (s, 1H), 8.36 (s, 1H), 8.05 (d, *J* = 8.8 Hz, 2H), 7.69 – 7.55 (m, 6H), 7.62 (d, *J* = 7.7 Hz, 1H), 7.37 7.35 (d, J = 7.9 Hz, 2H), 2.40 (s, 3H). ^{13}C NMR (101 MHz, DMSO-d_6): δ 166.4, 137.1, 135.3, 134.6, 132.1, 130.7, 130.0 (2C), 129.4, 129.2, 128.8 (2C), 128,83, 128.4 (2C), 128.34 (2C), 126.0, 118.4, 113.8, 21.2. IR (neat): ν_{max} 3612, 3227, 2919, 1639, 1616 cm^{-1}. HRMS (ASAP+): *m/z* calcd for $C_{21}H_{17}N_3O$ $[M+H]^+$: 328.1450, found 328.1450

II. X-Ray analysis and structural data of compound *5a*

X-ray single-crystal diffraction data were collected on a Rigaku Oxford Diffraction SuperNova diffractometer equipped with Atlas CCD detector and micro-focus Cu-K_α radiation (λ = 1.54184 Å). The structure was solved by dual-space algorithm and refined on F^2 by full matrix least-squares techniques using SHELX programs (G. M. Sheldrick, SHELXT 2014/5 and SHELXL 2016/4). All non-H atoms were refined anisotropically and multiscan empirical absorption was corrected using CrysAlisPro program (CrysAlisPro, Rigaku Oxford Diffraction, V1.171.40.45a, 2019). The H atoms were placed at calculated positions and refined using a riding model.

Crystallographic data for **5a**: $C_{14}H_{12}BrN_3O_2S$, M = 366.24, T=150K, colorless needle, 0.312 x 0.056 x 0.024 mm^3, monoclinic, space group $P2_1/c$, a = 14.3718(4) Å, b = 5.0528(2) Å, c = 19.9057(6) Å, β = 92.002(2)°, V = 1444.63(8) $Å^3$, Z = 4, ρcalc = 1.684 g/cm^3, μ = 5.306 mm^{-1}, F(000) = 736, θmin = 3.077°, θmax = 71.842°, 5496 reflections collected, 2750 unique (R_{int} = 0.0225), parameters / restraints = 191 / 0, R1 = 0.0350 and wR2 = 0.0887 using 2528 reflections with I>2σ(I), R1 = 0.0380 and wR2 = 0.0909 using all data, GOF = 1.093, $-0.538 < \Delta\rho < 1.134$ e.$Å^{-3}$.

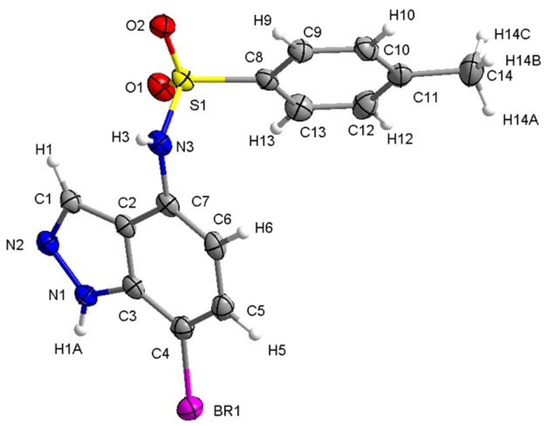

III. Computational procedure and representation of the f_k^- Fukui function for a radical attack

The theoretical calculations were carried out with the Gaussian16 program using the default algorithms, procedures, and thresholds.[1] The conformational analysis of **3a** and **3c** was performed at the MN15/6-31++G(d,p) level of theory.[2] The solvent effects (N,N-dimethylformamide) were taken into account using the SMD solvation continuum model, which provides valuable estimates of the impact of the interactions between the solute and the solvent.[3] The vibrational spectrum of each optimized conformer was computed to confirm that the stationary points located by the optimization procedure were minima in the potential energy surface. The absolute energetic minimum was selected for each compound on the basis of the lowest Gibbs energy value. The partial atomic charges computed within the natural population analysis model[4] have then been used to analyze the SMD-MN15/6-31++G(d,p) densities in terms of nucleophilicity, electrophilicity and radical reaction sites indices according to the conceptual density functional theory (DFT),[5] and represented on isodensity surfaces (0.001 e bohr^{-3}).

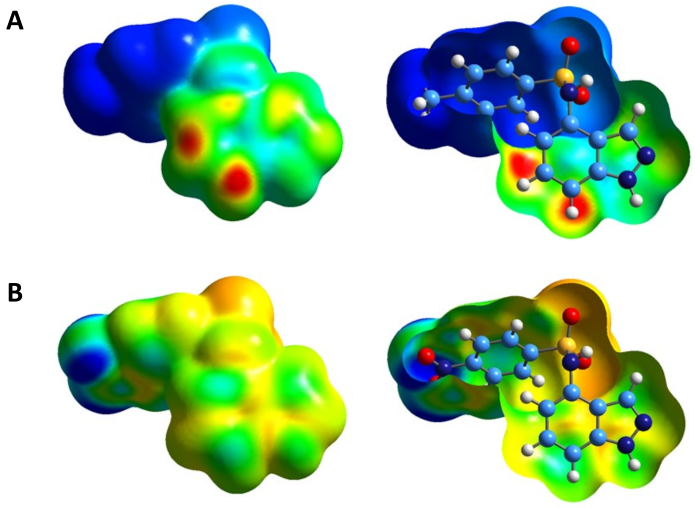

Figure S1. Representation of the f_k^- Fukui function for a radical attack: Radical index f_k^0 Fukui function of compounds **3a** (A) and **3c** (B), shown on the electron density isosurfaces (0.001 e bohr^{-3}) and calculated at the MN15/6-31++G(d,p) level of theory. The sites in red are the most prone to a radical attack.

Table S1. Cartesian coordinates of the absolute minima^a found for compounds **3a** and **3c** optimized at the SMD(DMF)/MN15/6-31++G(d,p) level of theory.

Compound **3a** : E_{el} = -1253.0916445 au				Compound **3c** : E_{el} = -1418.1316111 au			
6	2.659116	-2.54051	-0.033188	6	-2.021865	1.16968	-0.958118

6	1.335367	-2.547813	-0.43666	6	-1.203924	1.066447	0.169221
6	0.64298	-1.356182	-0.774077	6	-1.537125	0.282721	1.271314
6	1.27004	-0.124817	-0.690678	6	-2.735777	-0.429033	1.249376
6	2.619206	-0.080253	-0.274855	6	-3.54276	-0.323135	0.121127
6	3.588961	0.943088	-0.056763	6	-3.21797	0.461432	-0.985337
7	4.736024	0.419873	0.340717	16	0.335999	1.95191	0.170366
7	4.555239	-0.919075	0.395087	8	0.945204	1.769066	1.482562
6	3.290735	-1.283327	0.035296	7	-4.804854	-1.08003	0.093001
7	0.566545	1.077353	-1.019803	7	1.187564	1.161876	-1.023769
1	0.806321	-3.494047	-0.511954	6	1.720991	-0.135913	-0.729044
1	-0.388279	-1.407481	-1.116024	6	0.945626	-1.269081	-0.900731
1	3.187327	-3.455756	0.216881	6	1.467033	-2.555771	-0.607579
1	3.481807	2.014846	-0.174567	6	2.763733	-2.73767	-0.160185
1	5.318943	-1.520715	0.686969	6	3.546321	-1.577547	0.000341
16	-0.400793	1.769506	0.159117	6	3.046232	-0.283845	-0.265176
1	1.144553	1.809695	-1.444239	7	4.831213	-1.398216	0.42152
6	-1.817155	0.720341	0.164666	7	5.182261	-0.092889	0.447099
8	-0.746146	3.081709	-0.381972	6	4.128	0.593708	0.041063
8	0.228604	1.686279	1.474447	8	0.12484	3.300291	-0.343489
6	-2.072074	-0.08779	1.269685	1	0.826471	-3.421295	-0.753396
6	-3.182218	-0.934443	1.238593	1	-0.068901	-1.168567	-1.279729
6	-4.025589	-0.978806	0.121247	1	3.162012	-3.724706	0.055018
6	-3.736477	-0.152757	-0.980644	1	4.162094	1.674855	-0.021133
6	-2.637141	0.69761	-0.968314	1	5.499442	-2.107761	0.705618
1	-1.411057	-0.05917	2.131304	1	1.846972	1.814765	-1.459748
1	-3.394596	-1.57173	2.093974	1	-0.87099	0.223646	2.126448
6	-5.229385	-1.880746	0.094226	1	-3.031552	-1.05368	2.08477
1	-5.238101	-2.555612	0.955	1	-3.881703	0.511739	-1.841242
1	-6.153138	-1.289972	0.114209	1	-1.725895	1.785147	-1.80347
1	-5.248819	-2.481468	-0.821773	8	-5.508515	-0.989354	-0.908517
1	-4.384279	-0.182331	-1.854295	8	-5.088818	-1.763982	1.071746
1	-2.410525	1.329121	-1.824392				

[a] exhibiting the most negative electronic energies among the structures identified within the conformational analysis, and without any imaginary frequency.

IV. References

1. M. J. Frisch, G. W. Trucks, H. B. Schlegel, G. E. Scuseria, M. A. Robb, J. R. Cheeseman, G. Scalmani, V. Barone, G. A. Petersson, H. Nakatsuji, X. Li, M. Caricato, A. Marenich, J. Bloino, B. G. Janesko, R. Gomperts, B. Mennucci, H. P. Hratchian, J. V. Ortiz, A. F. Izmaylov, J. L. Sonnenberg, D. Williams-Young, F. Ding, F. Lipparini, F. Egidi, J. Goings, B. Peng, A. Petrone, T. Henderson, D. Ranasinghe, V. G. Zakrzewski, J. Gao, N. Rega, G. Zheng, W. Liang, M. Hada, M. Ehara, K. Toyota, R. Fukuda, J. Hasegawa, M. Ishida, T. Nakajima, Y. Honda, O. Kitao, H. Nakai, T. Vreven, K. Throssell, J. A. Montgomery, Jr., J. E. Peralta, F. Ogliaro, M. Bearpark, J. J. Heyd, E. Brothers, K. N. Kudin, V. N. Staroverov, T. Keith, R. Kobayashi, J. Normand, K. Raghavachari, A. Rendell, J. C. Burant, S. S. Iyengar, J. Tomasi, M. Cossi, J. M. Millam, M. Klene, C. Adamo, R. Cammi, J. W. Ochterski, R. L. Martin, K. Morokuma, O. Farkas, J. B. Foresman, and D. J. Fox, Gaussian , Inc., Wallingford CT, 2016
2. H. S. Yu, X. He, S. L. Li, D. G. Truhlar, MN15: A Kohn–Sham Global-Hybrid Exchange–Correlation Density Functional with Broad Accuracy for Multi-Reference and Single-Reference Systems and Noncovalent Interactions. *Chem. Sci.* 2016, **7** , 5032-5051.
3. A. V. Marenich, C. J. Cramer, D. G. Truhlar, Universal Solvation Model Based on Solute Electron Density and on a Continuum Model of the Solvent Defined by the Bulk Dielectric Constant and Atomic Surface Tensions. *J. Phys. Chem. B* 2009, **113**, 6378-6396.
4. A. E. Reed, L. A. Curtiss, F. Weinhold, Intermolecular Interactions from a Natural Bond Orbital, Donor-Acceptor Viewpoint. *Chem. Rev.* 1988, **88**, 899-926.
5. P. Geerlings, F. De Proft, W. Langenaeker, Conceptual Density Functional Theory. *Chem. Rev.* 2003, **103**, 1793-1874.

V. ^{1}H and ^{13}C NMR Spectra

***N*-(1H-4-Indazolyl)-4-methylbenzenesulfonamide 3a:**

^{1}H NMR (300 MHz, DMSO-d_6)

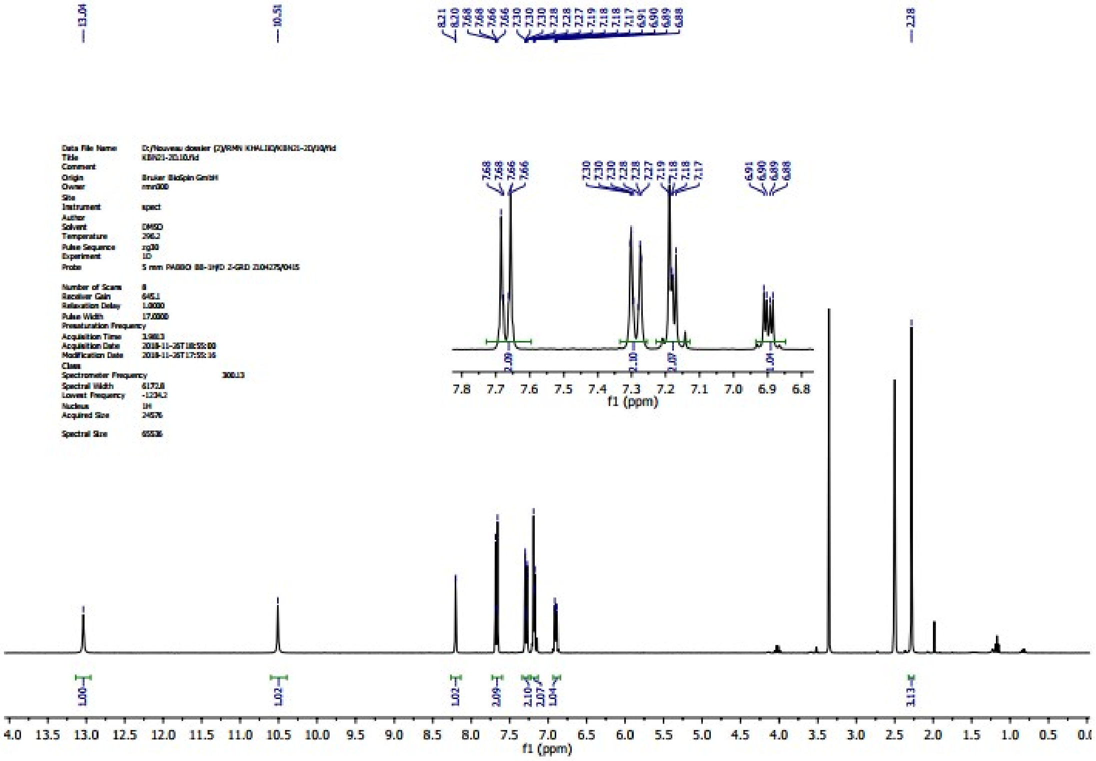

^{13}C NMR (75 MHz, DMSO-d_6)

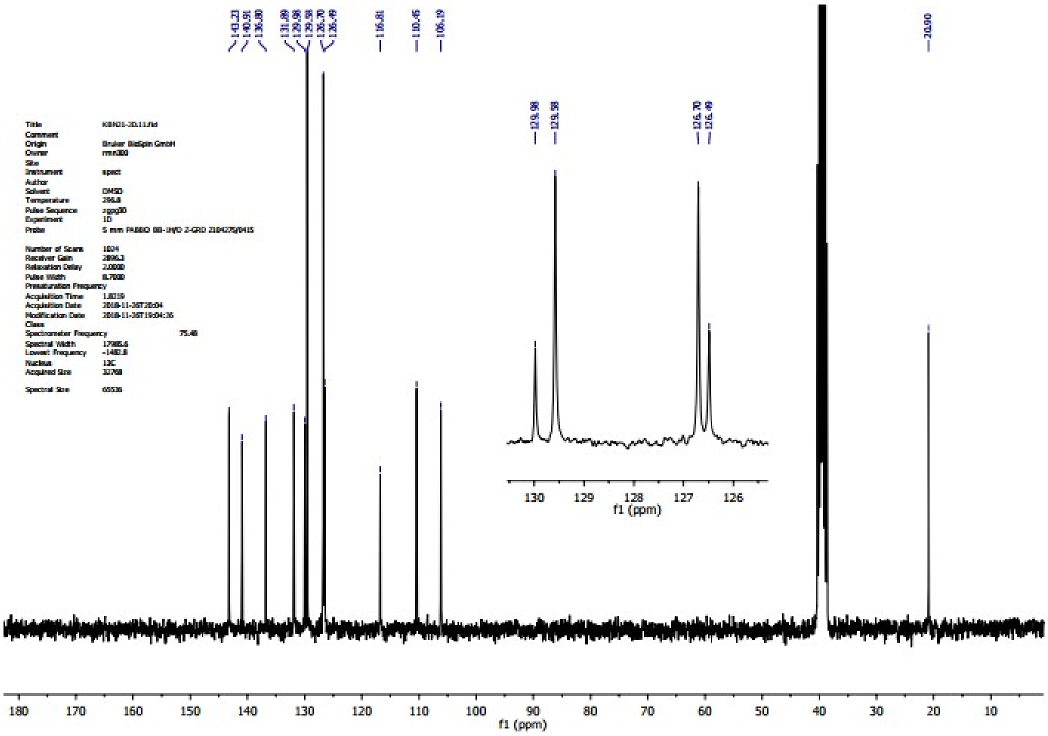

N-(1H-4-Indazolyl)-4-methoxylbenzenesulfonamide 3b

^{1}H NMR (300 MHz, DMSO-d_6)

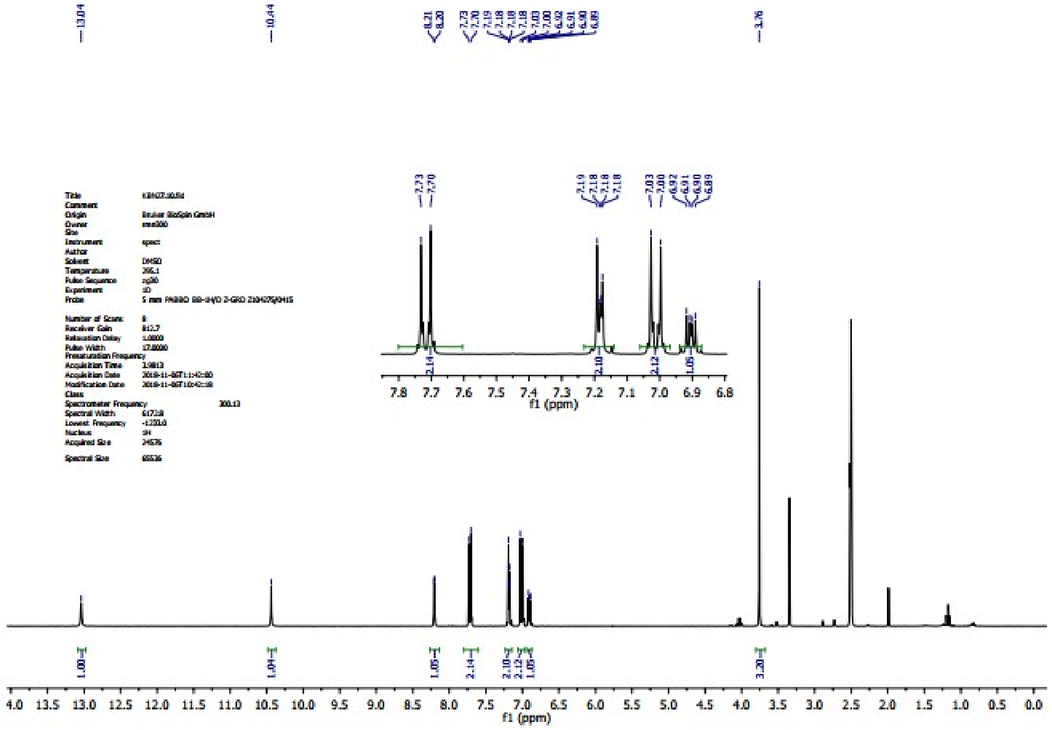

^{13}C NMR (75 MHz, DMSO-d_6)

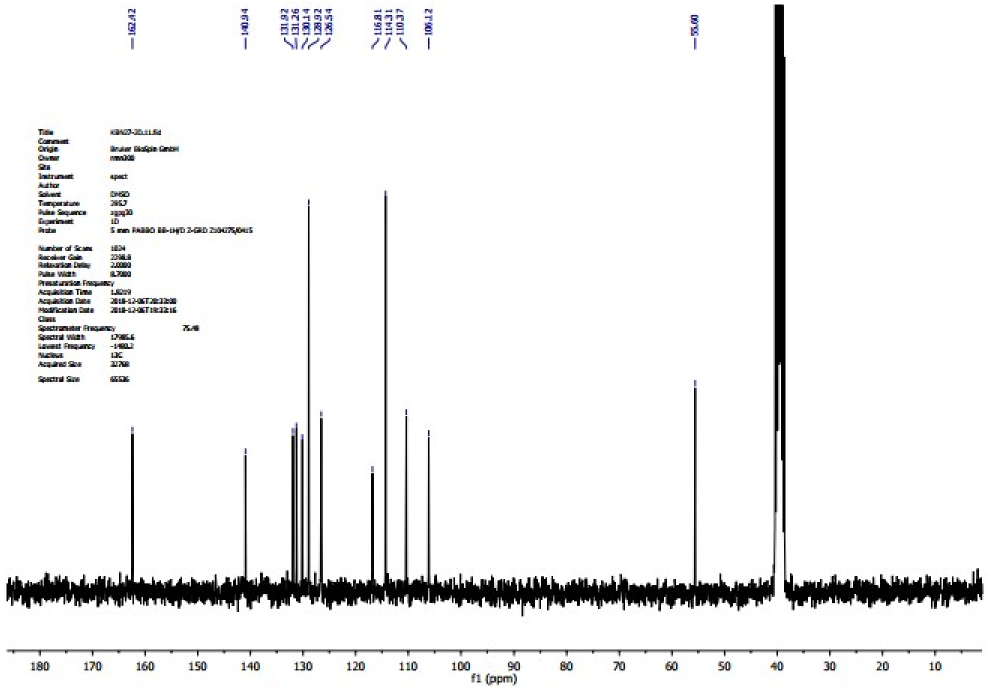

N-(1H-4-Indazolyl)-4-nitrobenzenesulfonamide 3c

^{1}H NMR (300 MHz, DMSO-d_6)

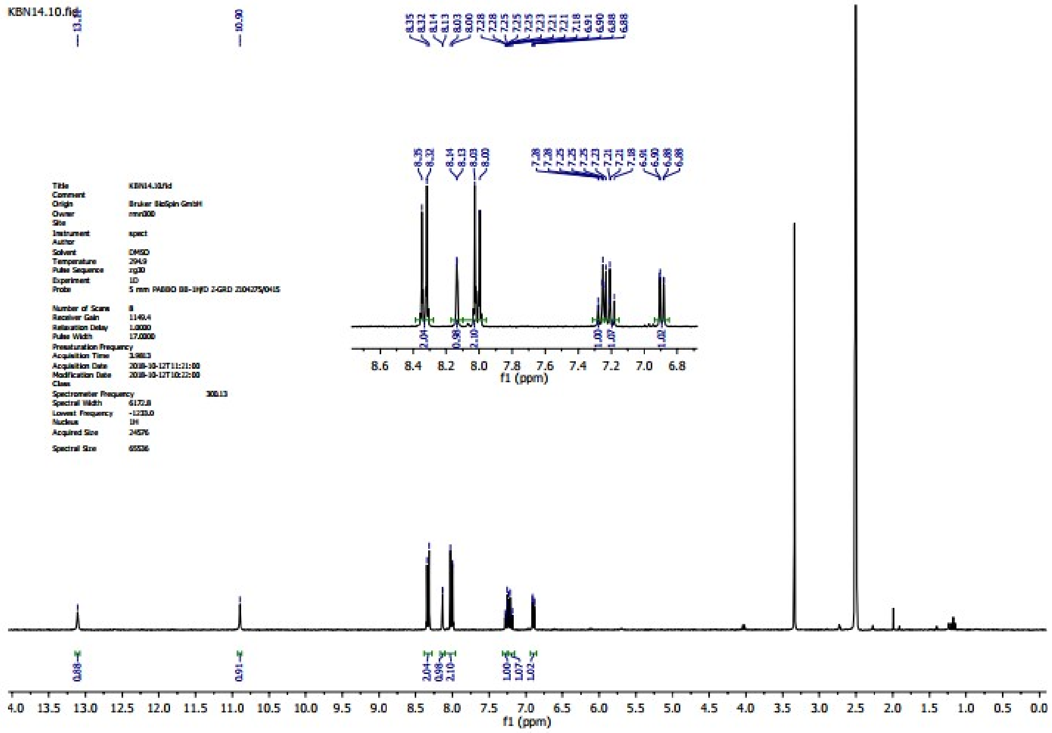

^{13}C NMR (101 MHz, DMSO-d_6)

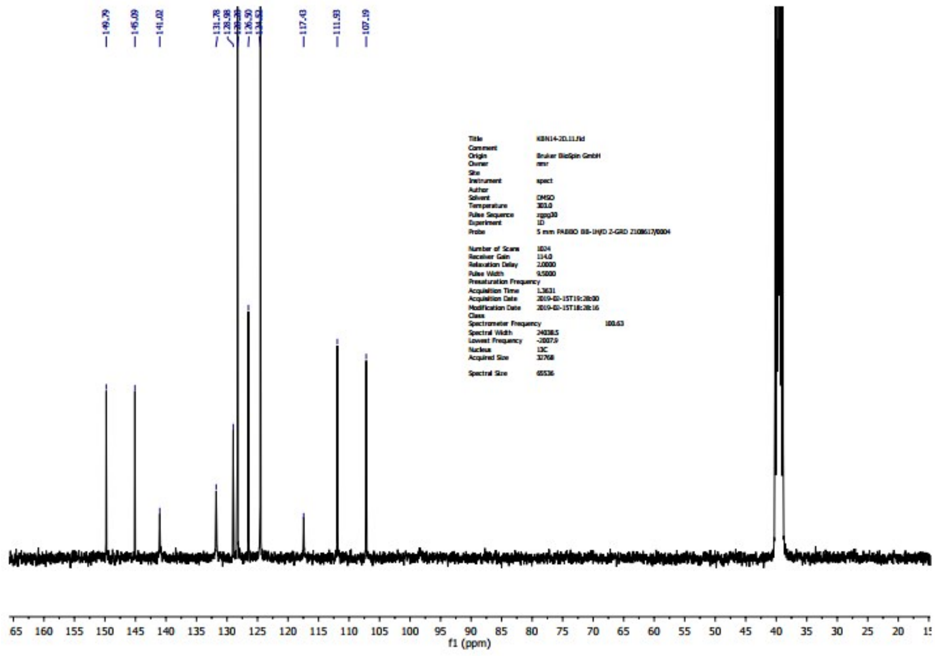

N-(1H-4-Indazolyl)-4-methoxybenzamide 4a

^{1}H NMR (300 MHz, DMSO-d_6)

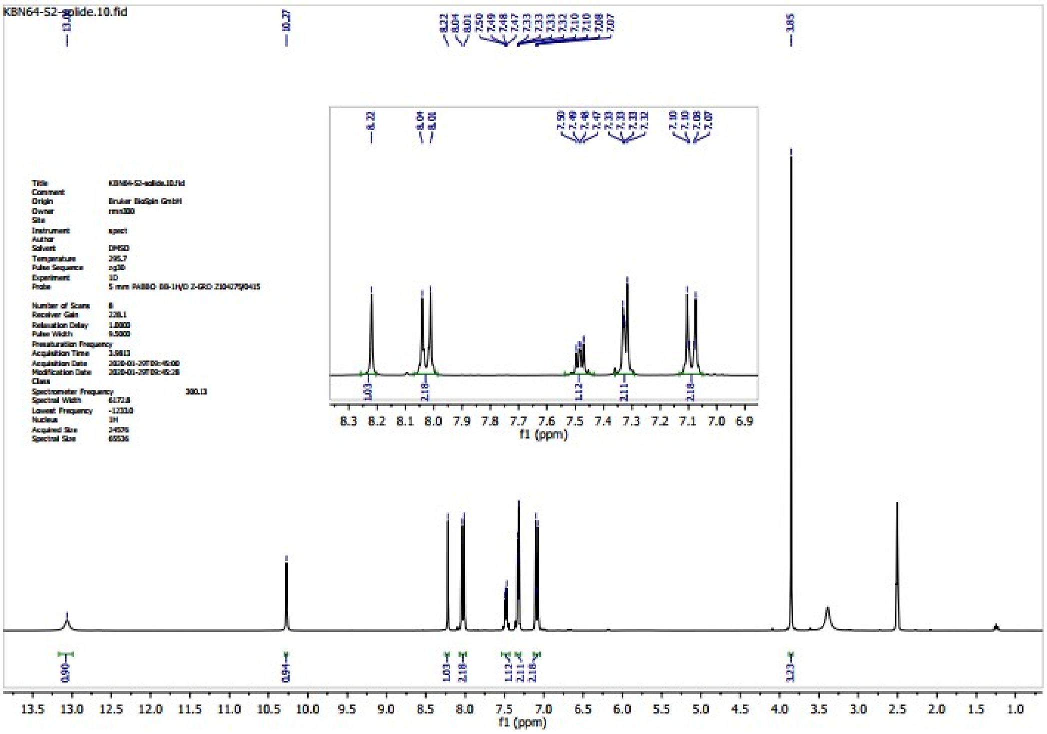

^{13}C NMR (101 MHz, DMSO-d_6)

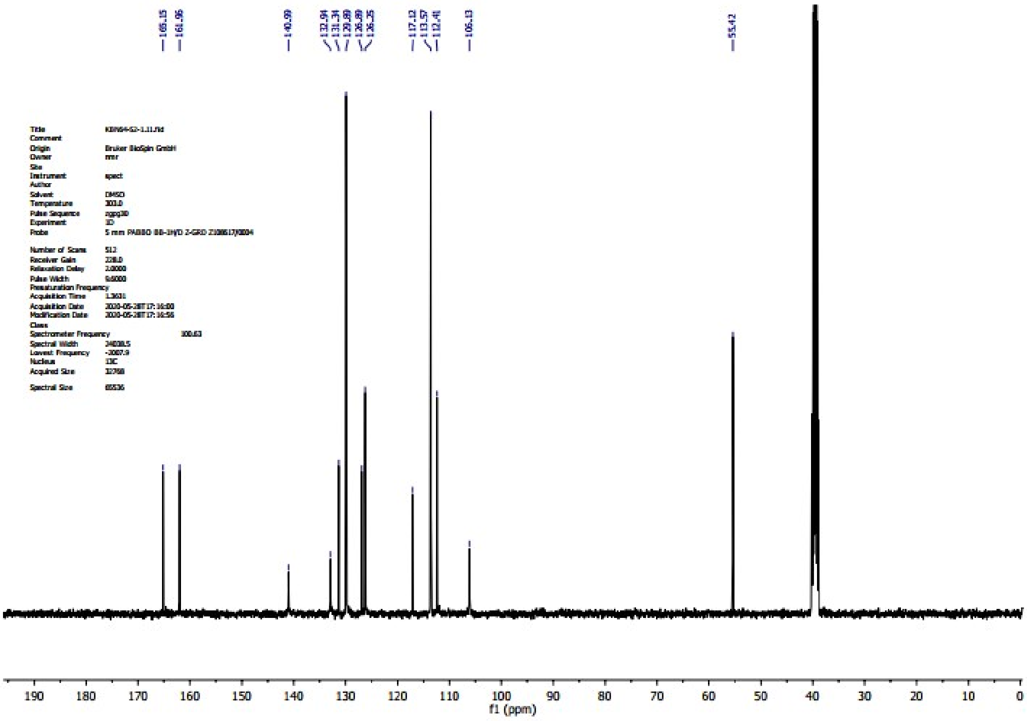

N-(1H-4-Indazolyl)-2-methoxybenzamide 4b

^{1}H NMR (300 MHz, DMSO-d_6)

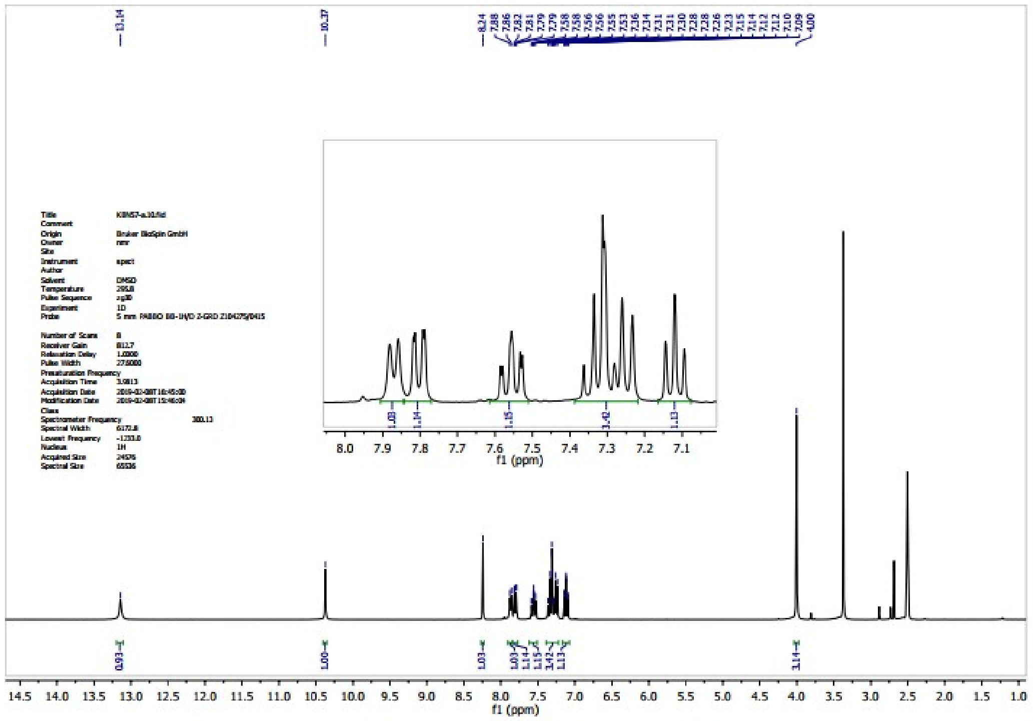

^{13}C NMR (101 MHz, DMSO-d_6)

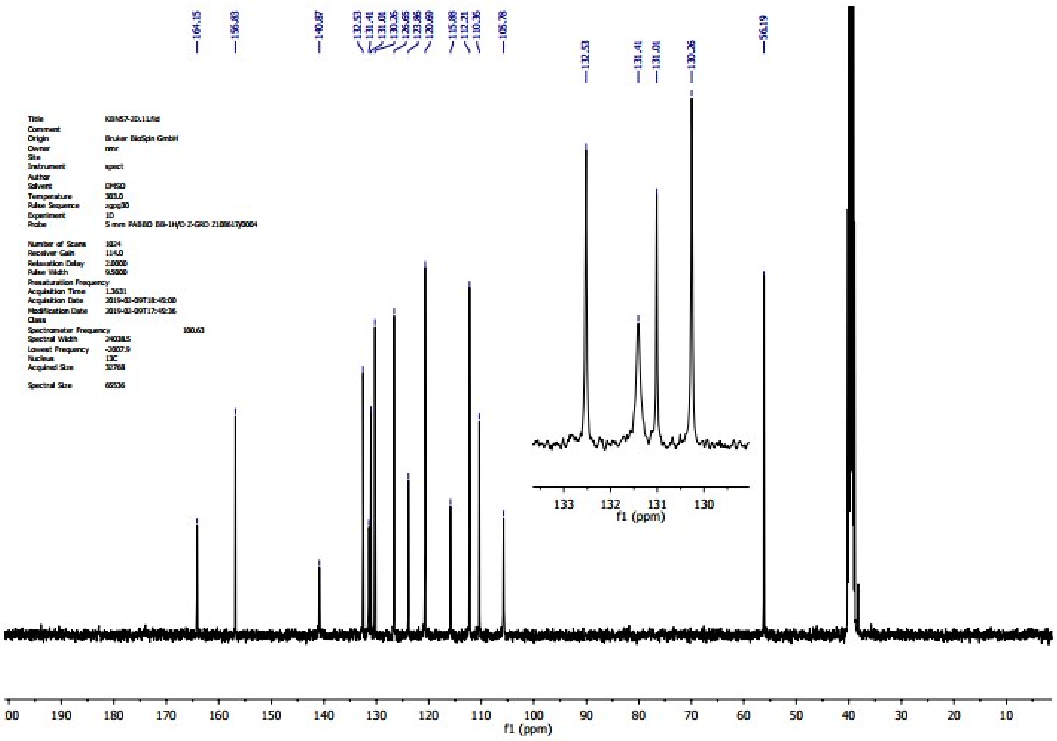

N-(1H-4-Indazolyl)benzamide 4c

^{1}H NMR (400 MHz, DMSO-d_6)

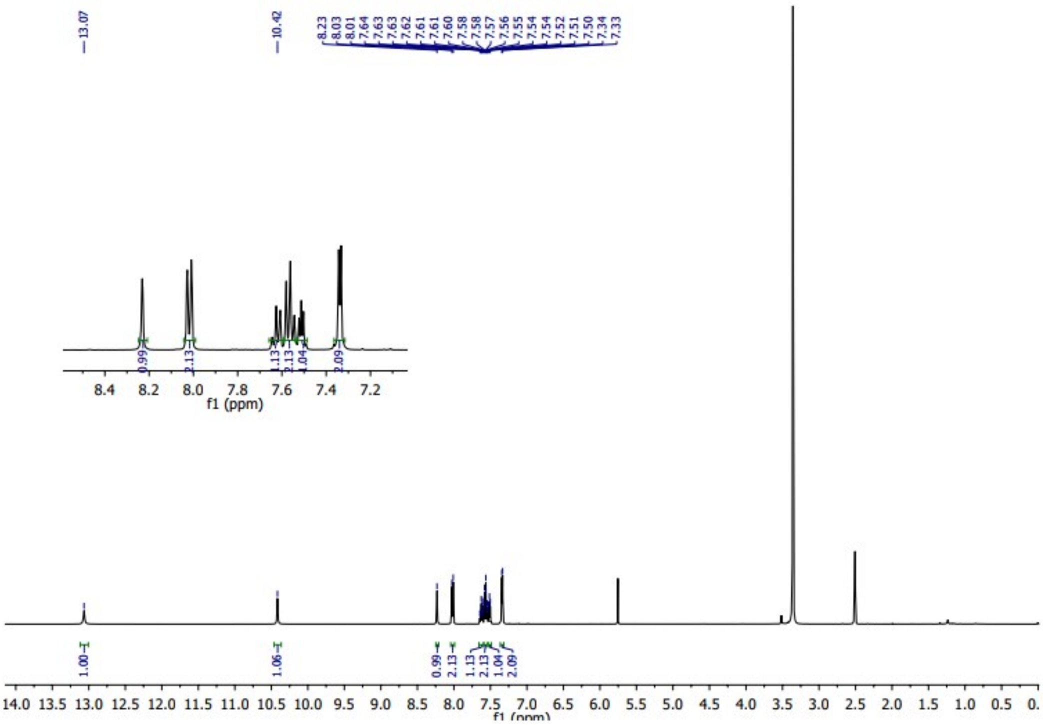

^{13}C NMR (101 MHz, DMSO-d_6)

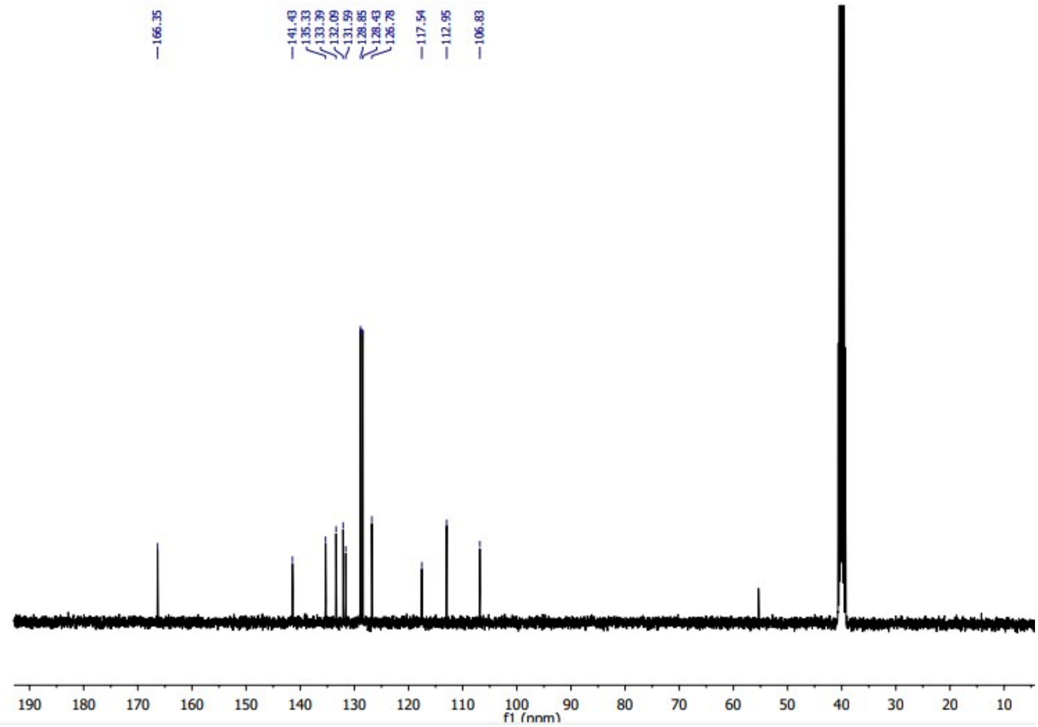

N-(7-bromo-1H-indazol-4-yl)-4-methylbenzenesulfonamide 5a

^{1}H NMR (300 MHz, DMSO-d_6)

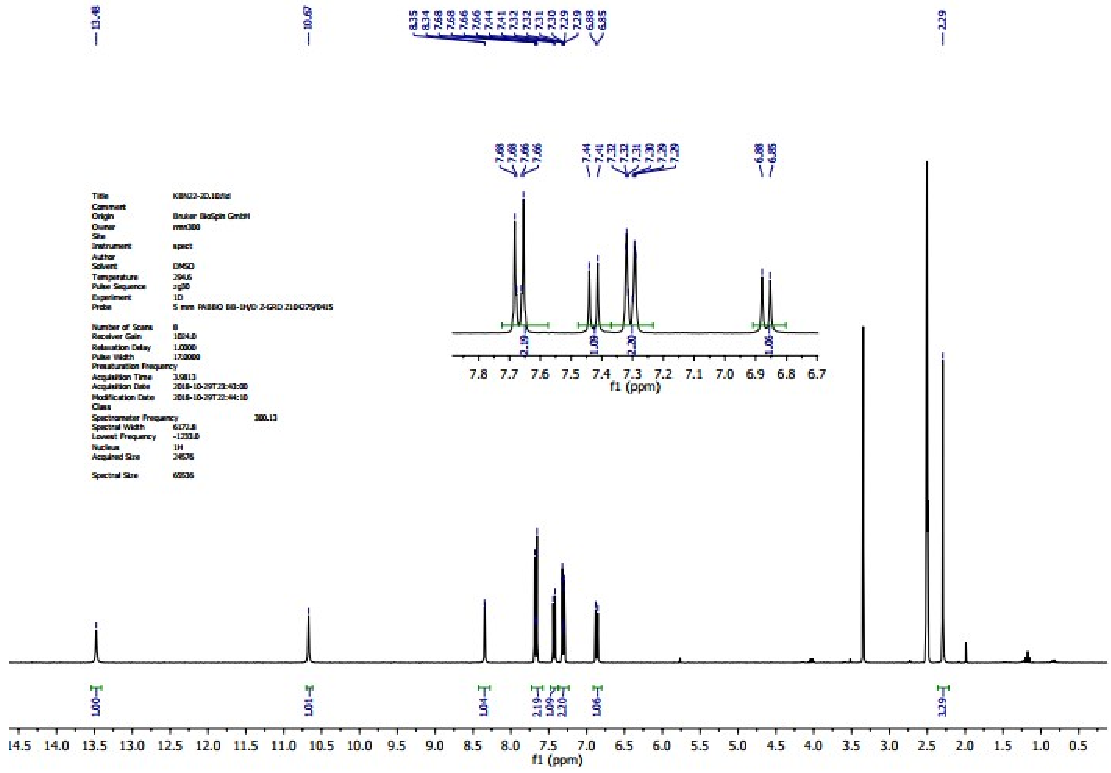

^{13}C NMR (75 MHz, DMSO-d_6)

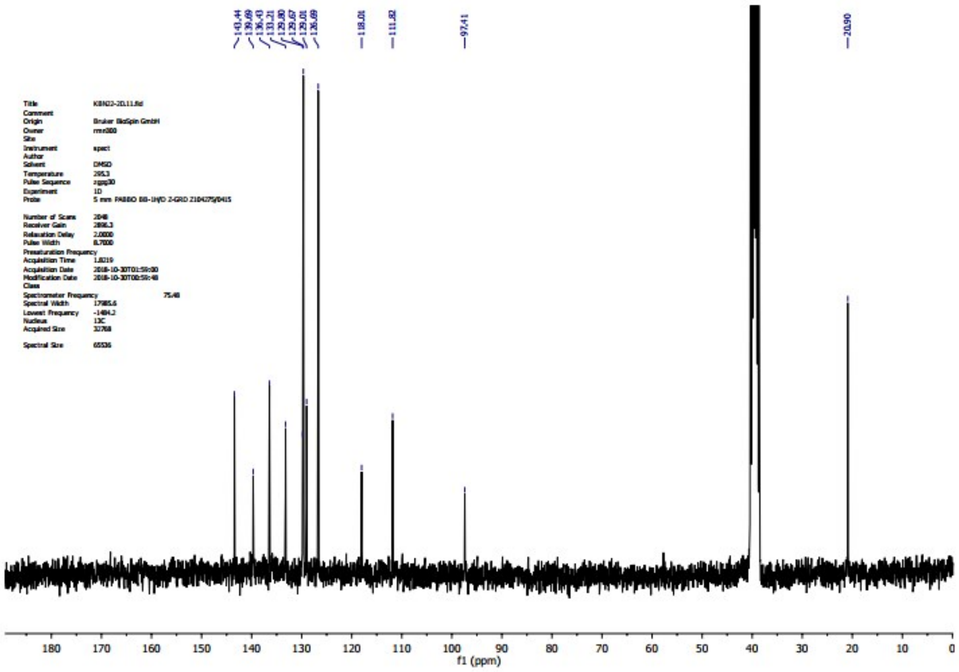

N-(7-bromo-1H-indazol-4-yl)-4-methoxybenzenesulfonamide 5b

^{1}H NMR (300 MHz, DMSO-d_6)

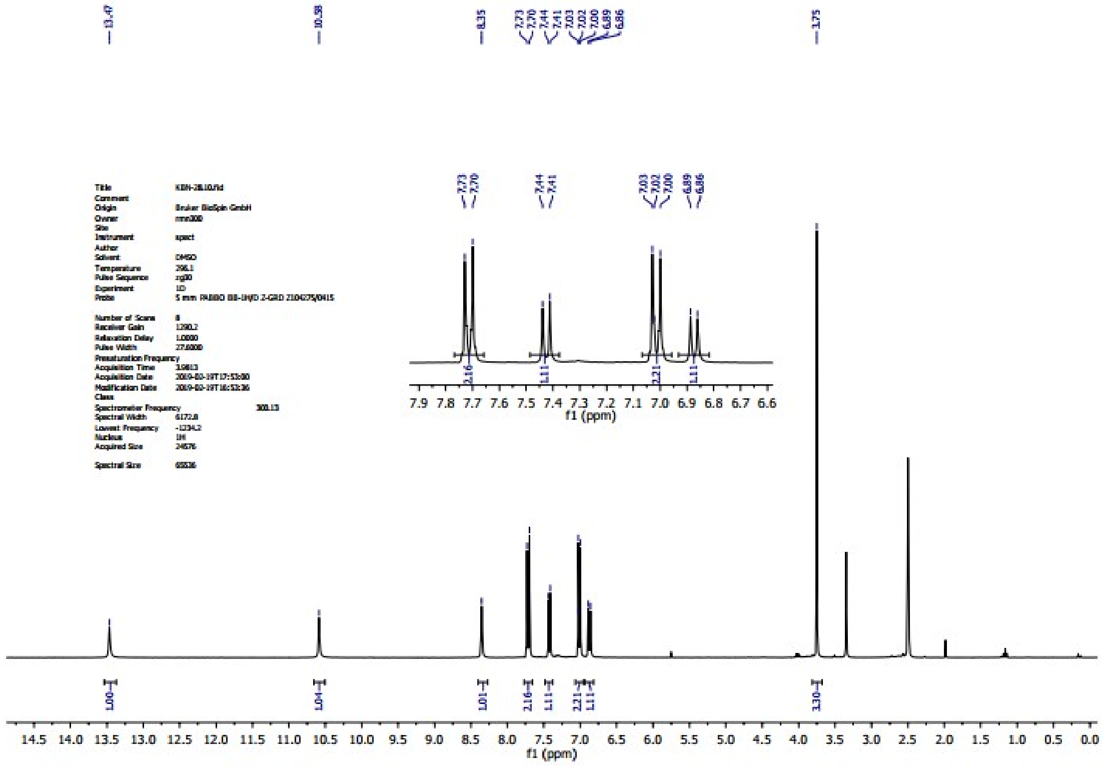

^{13}C NMR (101 MHz, DMSO-d_6)

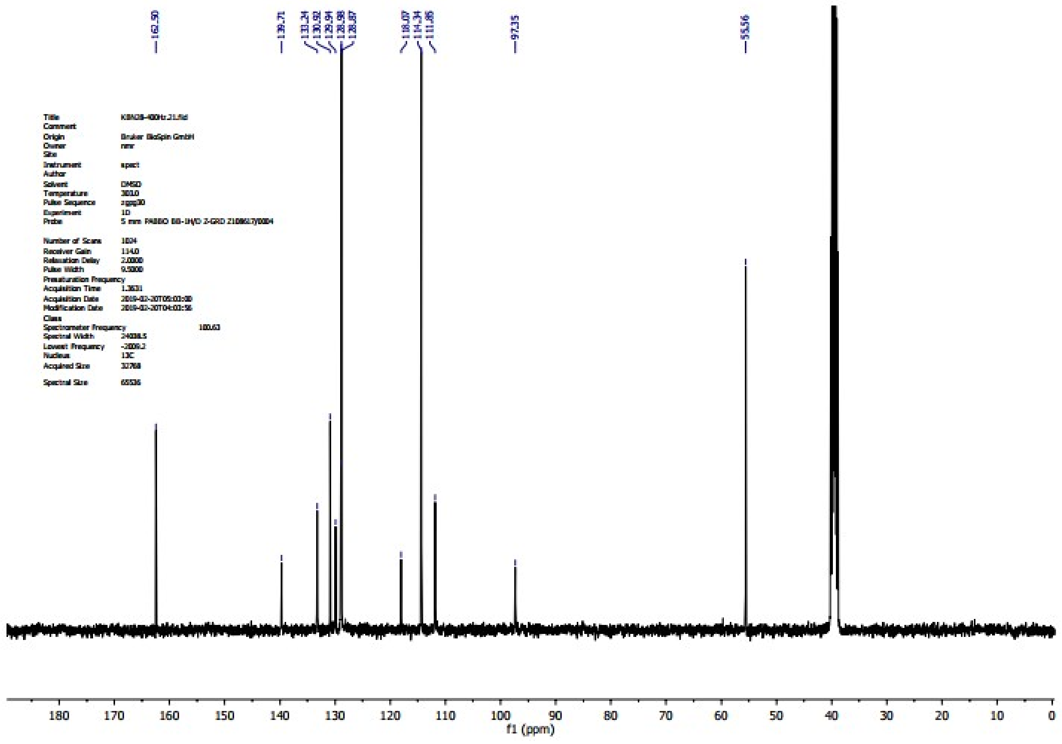

N-(7-bromo-1H-indazol-4-yl)-4-nitrobenzenesulfonamide 5c

^{1}H NMR (300 MHz, DMSO-d_6)

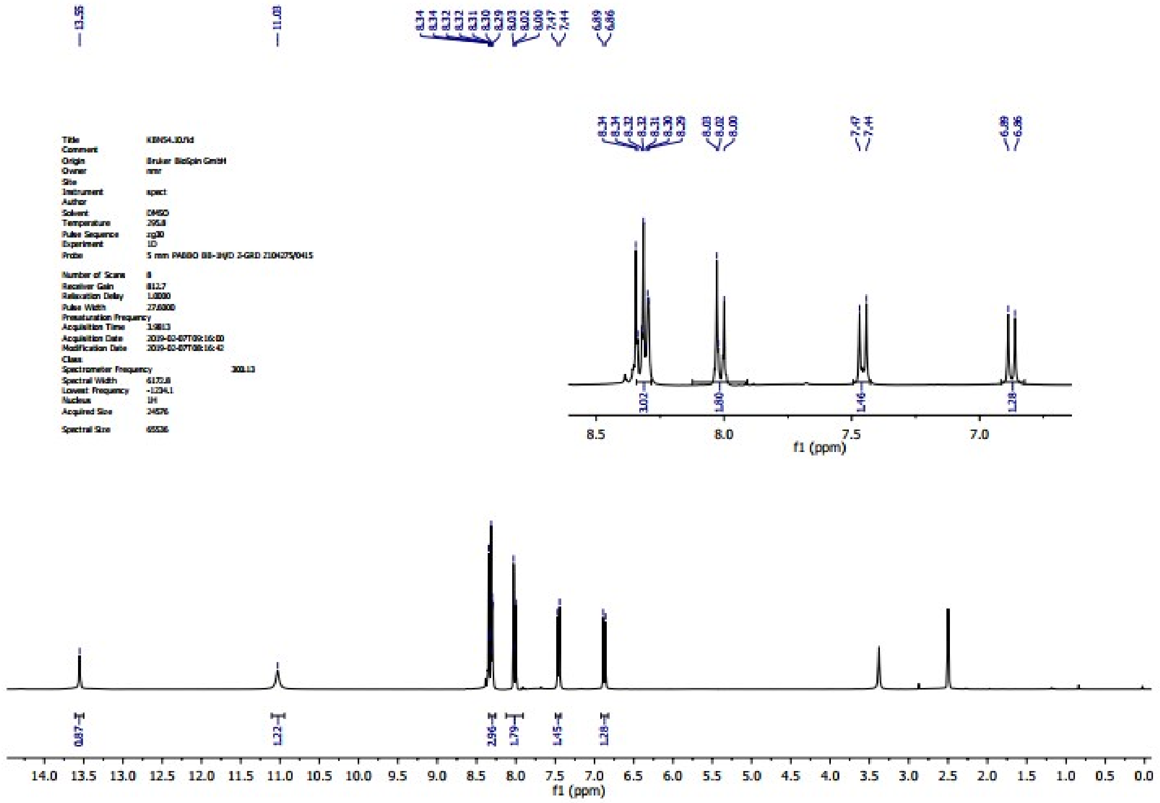

^{13}C NMR (101 MHz, DMSO-d_6)

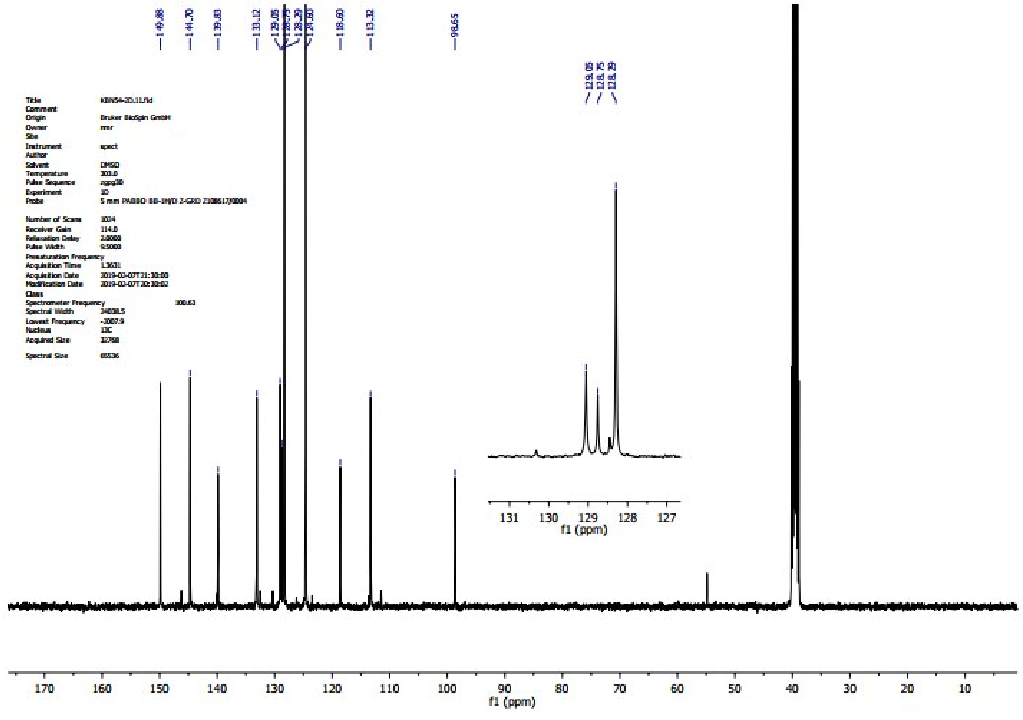

N-(5,7-dibromo-1H-indazol-4-yl)-4-methylbenzenesulfonamide 6a

^{1}H NMR (300 MHz, DMSO-d_6)

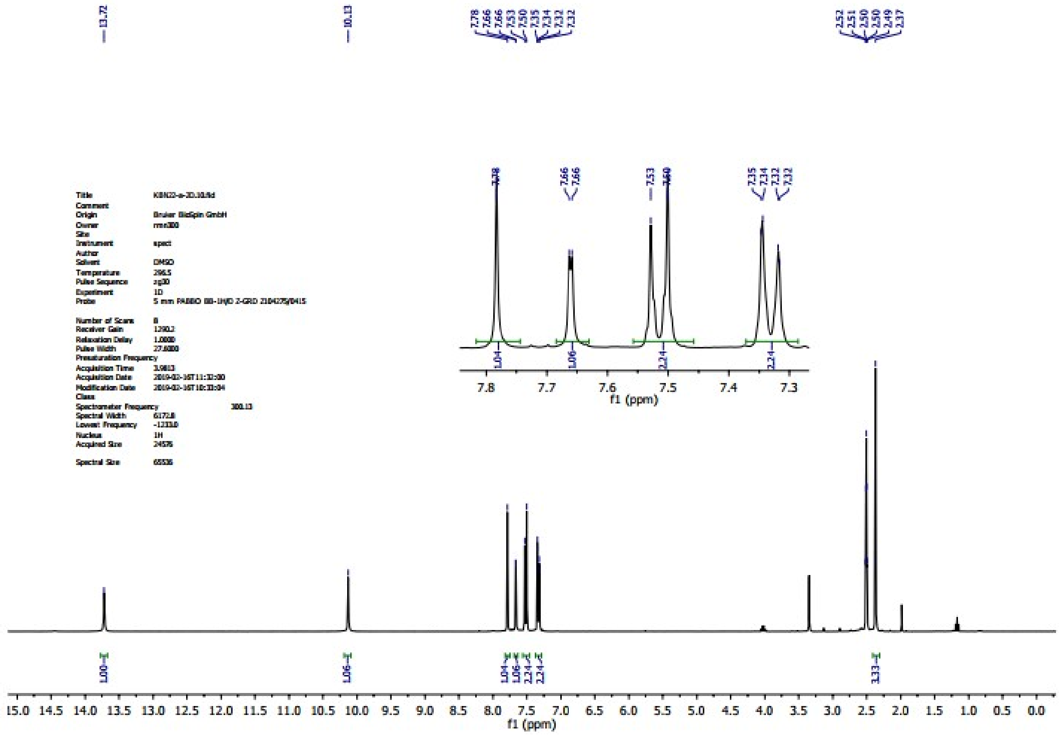

^{13}C NMR (75 MHz, DMSO-d_6)

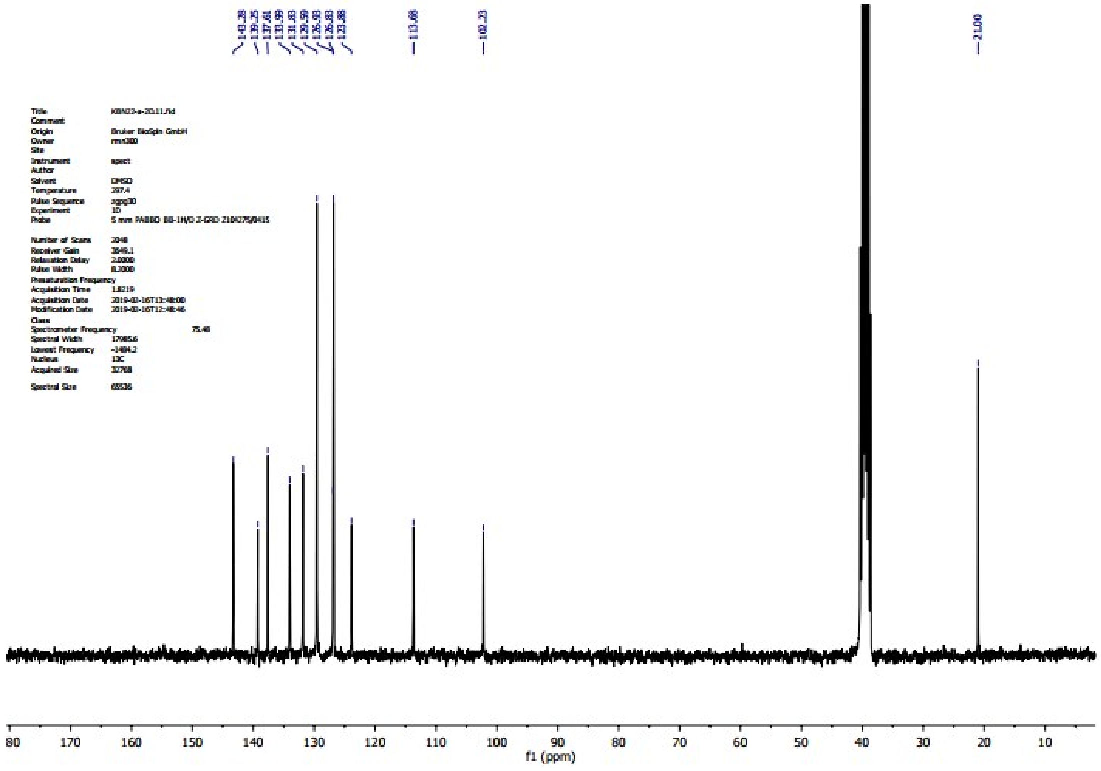

***N*-(5,7-dibromo-1*H*-indazol-4-yl)-4-methoxybenzenesulfonamide 6b**

^{1}H NMR (300 MHz, DMSO-d_6)

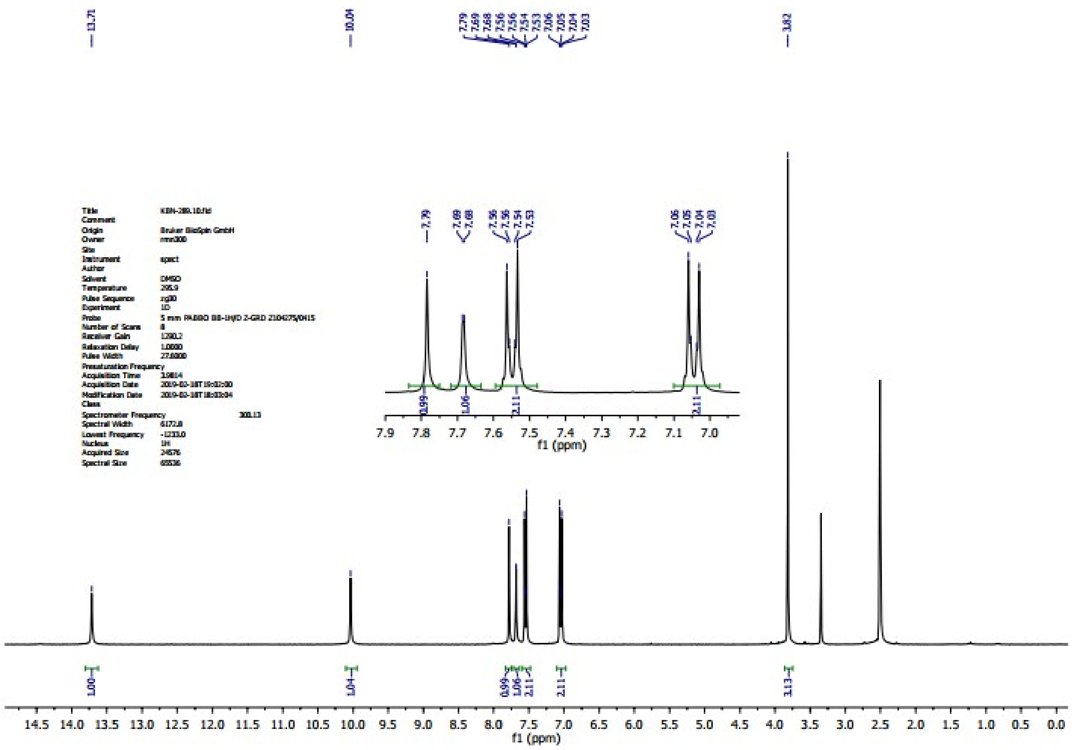

^{13}C NMR (75 MHz, DMSO-d_6)

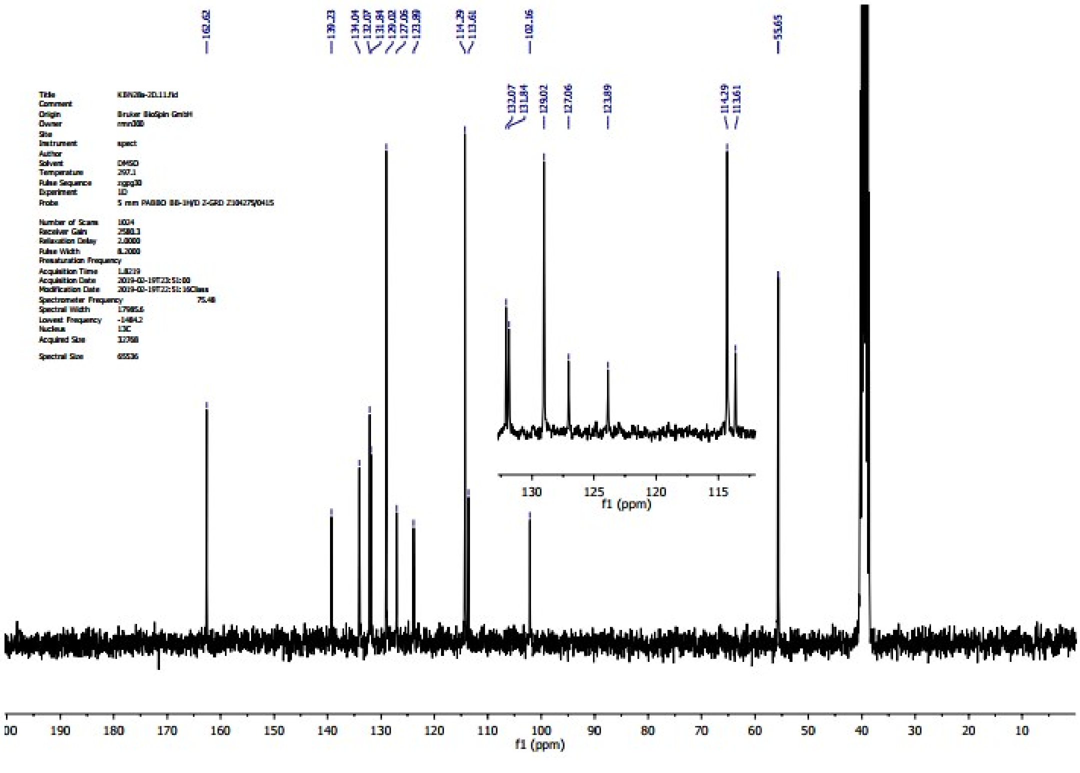

***N*-(5,7-dibromo-1*H*-indazol-4-yl)-4-nitrobenzenesulfonamide 6c**:

^{1}H NMR (300 MHz, DMSO-d_6)

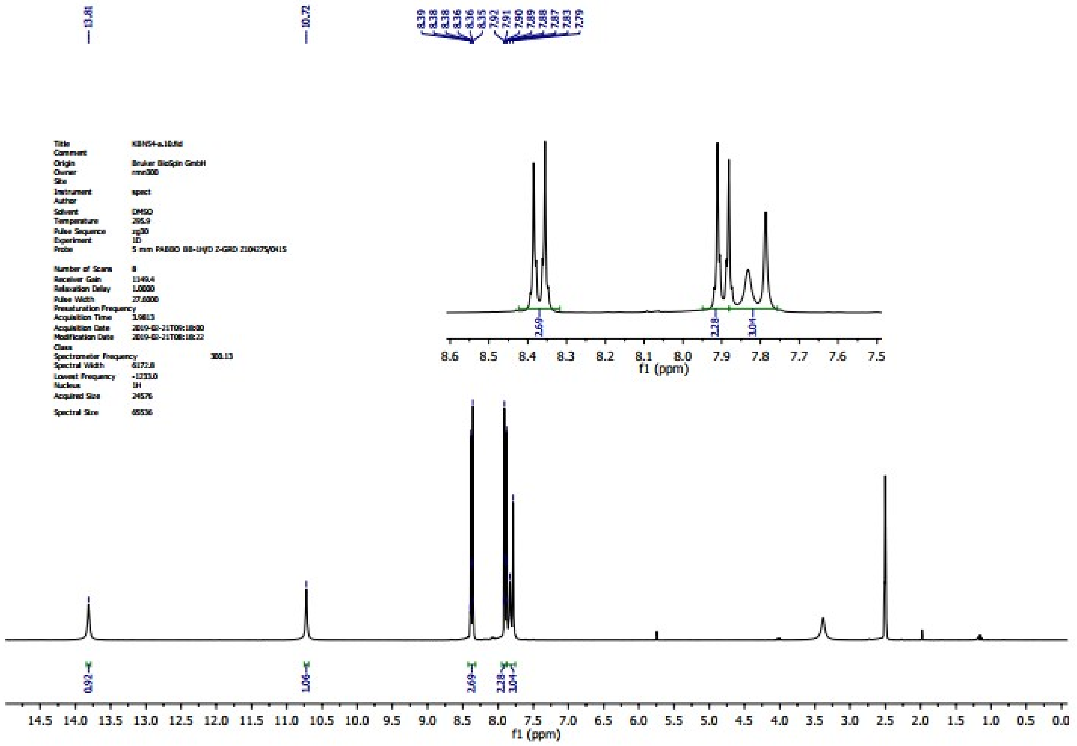

^{13}C NMR (75 MHz, DMSO-d_6)

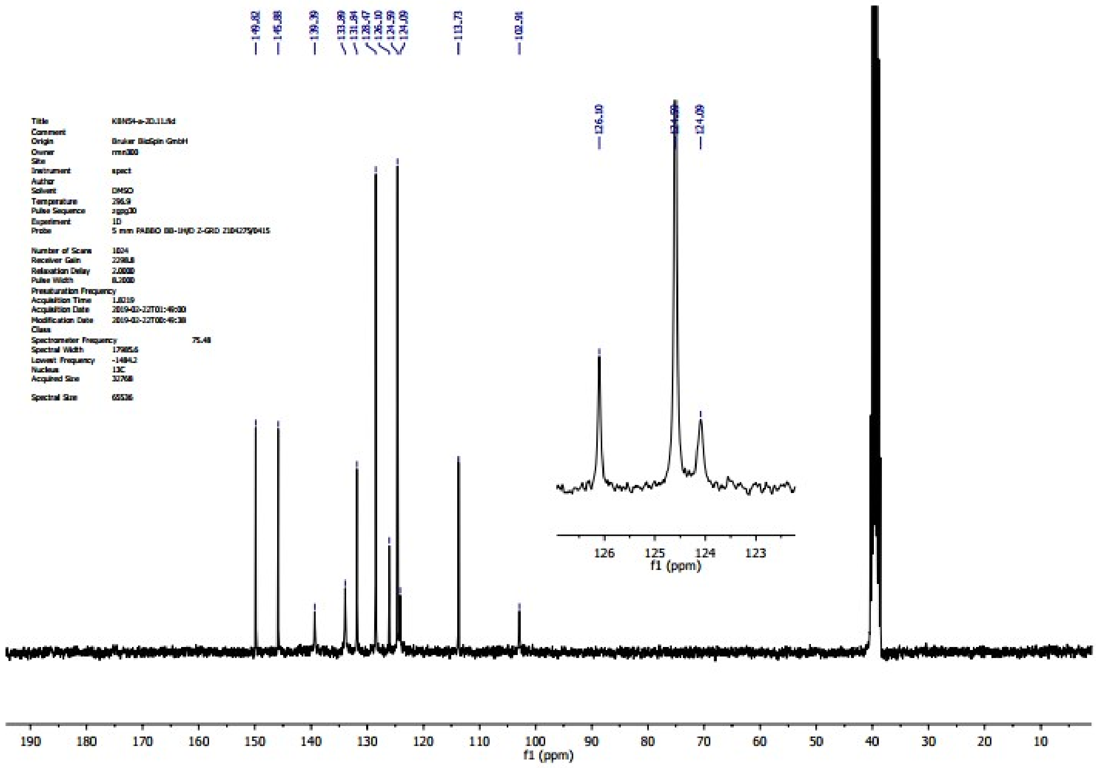

N-(4-bromo-1H-indazol-7-yl)-4-methoxybenzamide 7a

^{1}H NMR (300 MHz, DMSO-d_6)

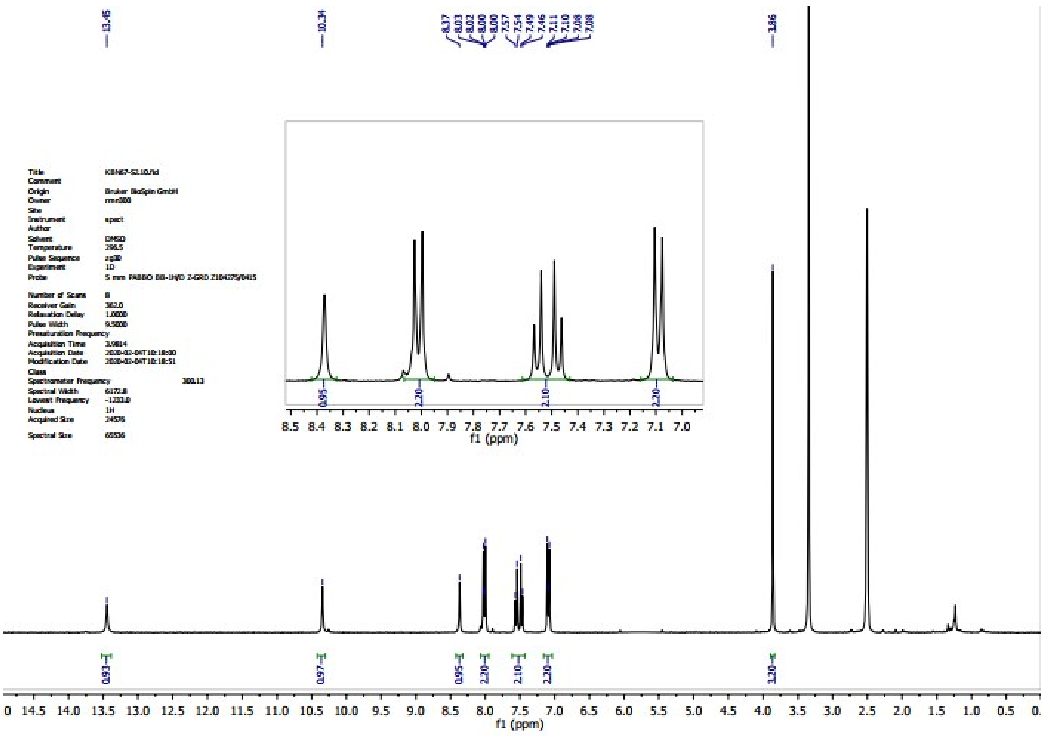

^{13}C NMR (101 MHz, DMSO-d_6)

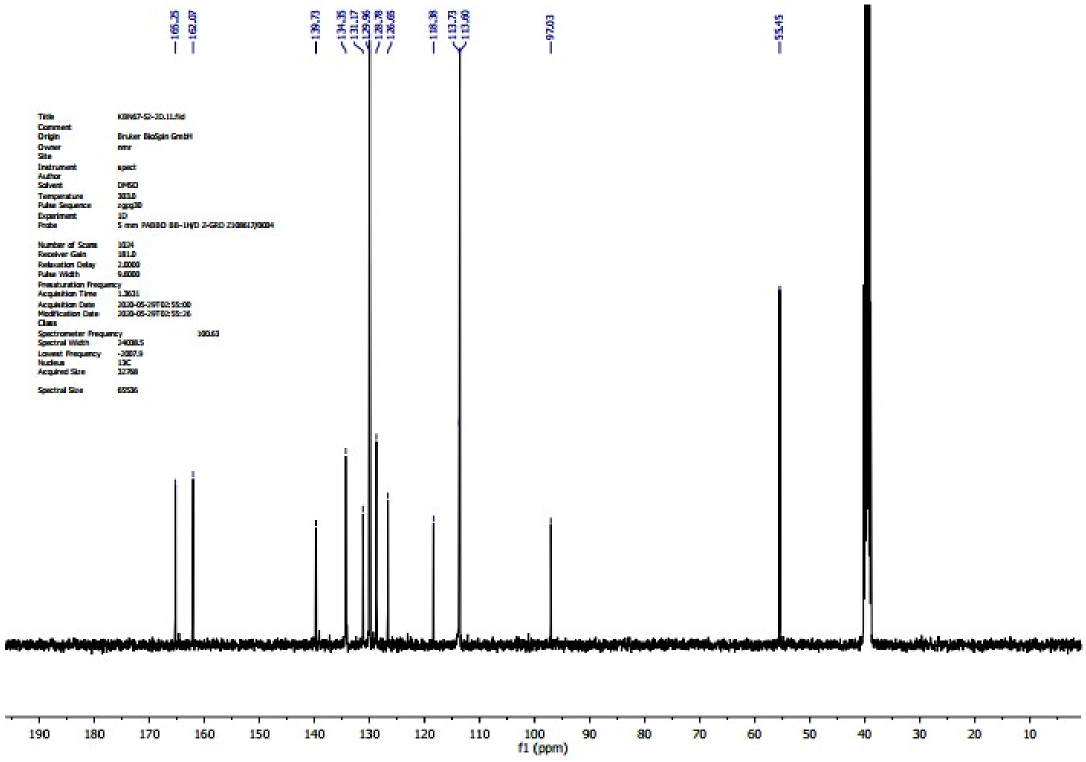

N-(4-bromo-1H-indazol-7-yl)-2-methoxybenzamide 7b

^{1}H NMR (300 MHz, DMSO-d_6)

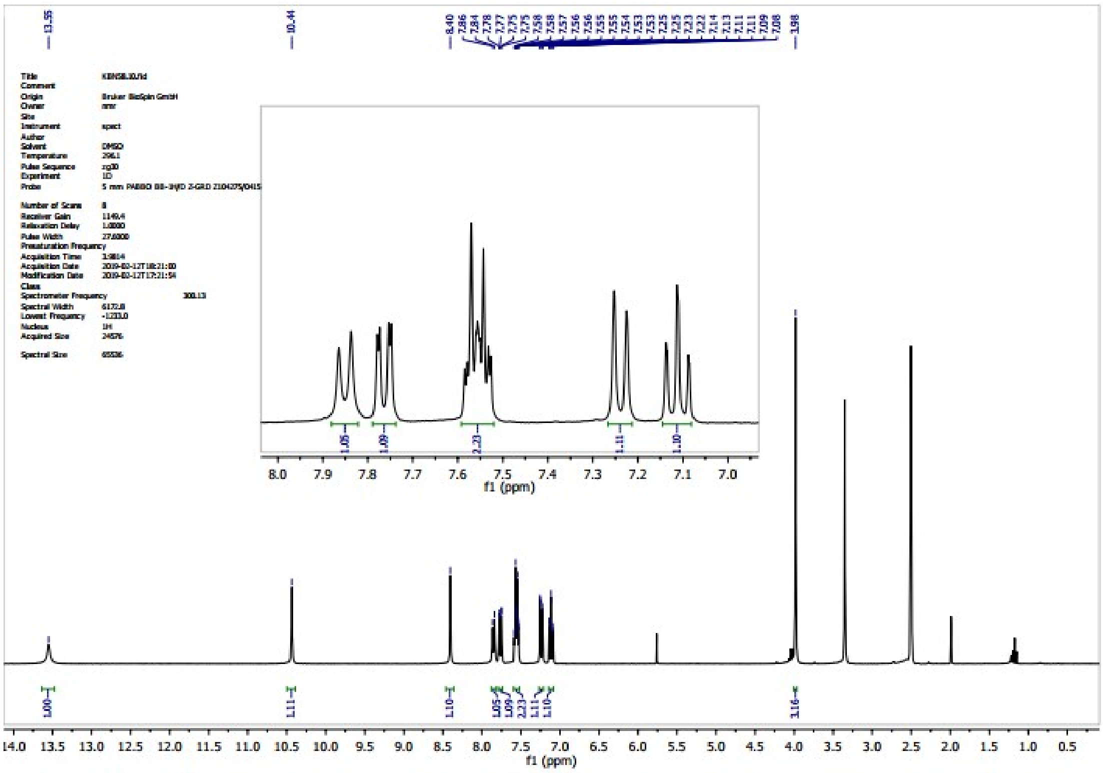

^{13}C NMR (101 MHz, DMSO-d_6)

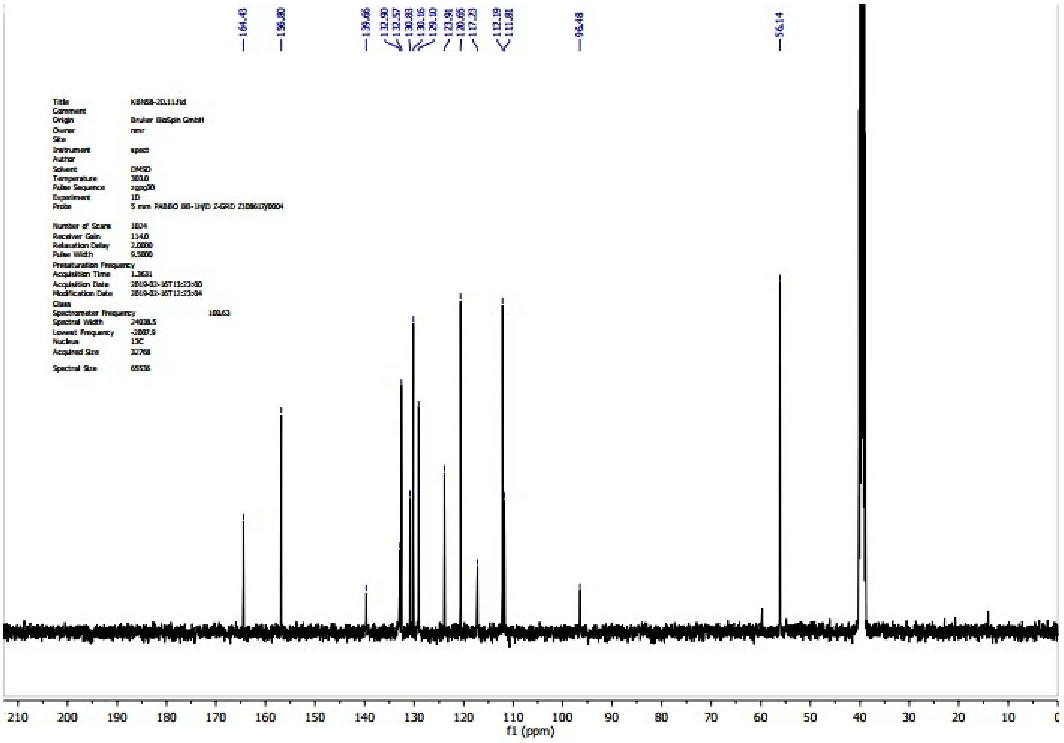

***N*-(4-bromo-1*H*-indazol-7-yl)benzamide 7c**

^{1}H NMR (400 MHz, DMSO-d_6)

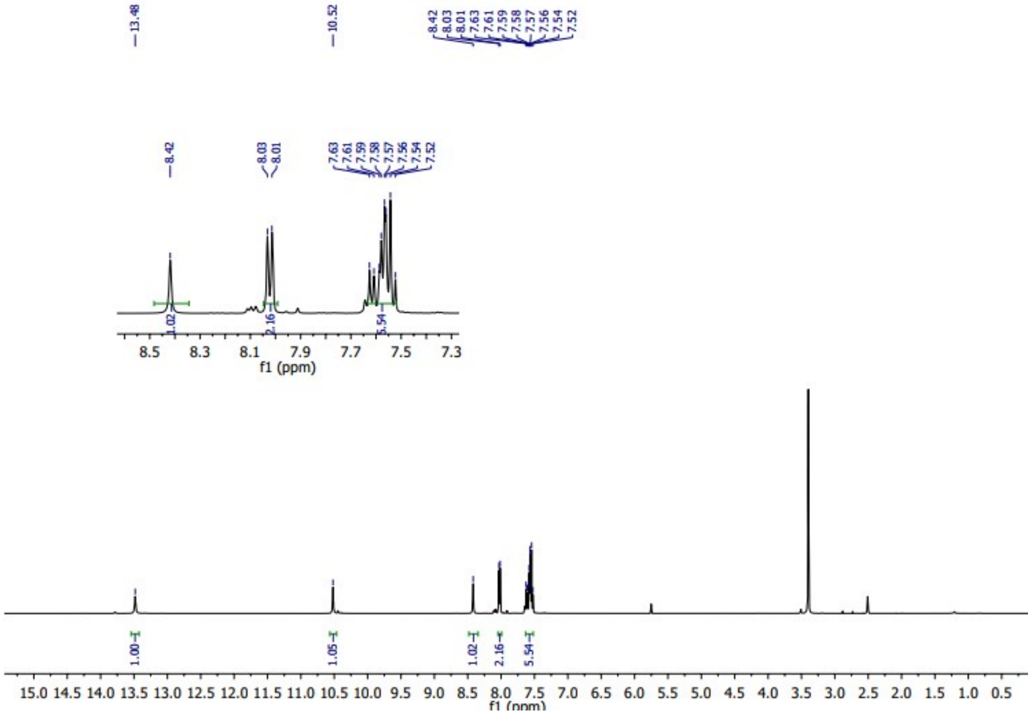

^{13}C NMR (101 MHz, DMSO-d_6)

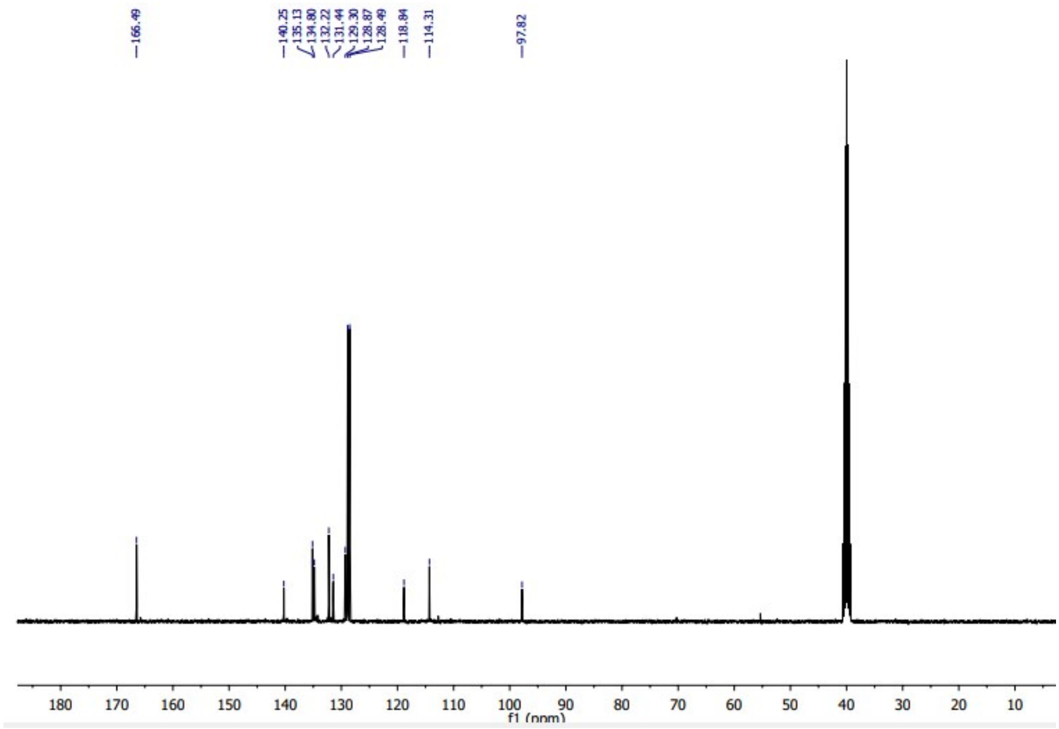

Compound 8aa

^{1}H NMR (400 MHz, DMSO-d_6)

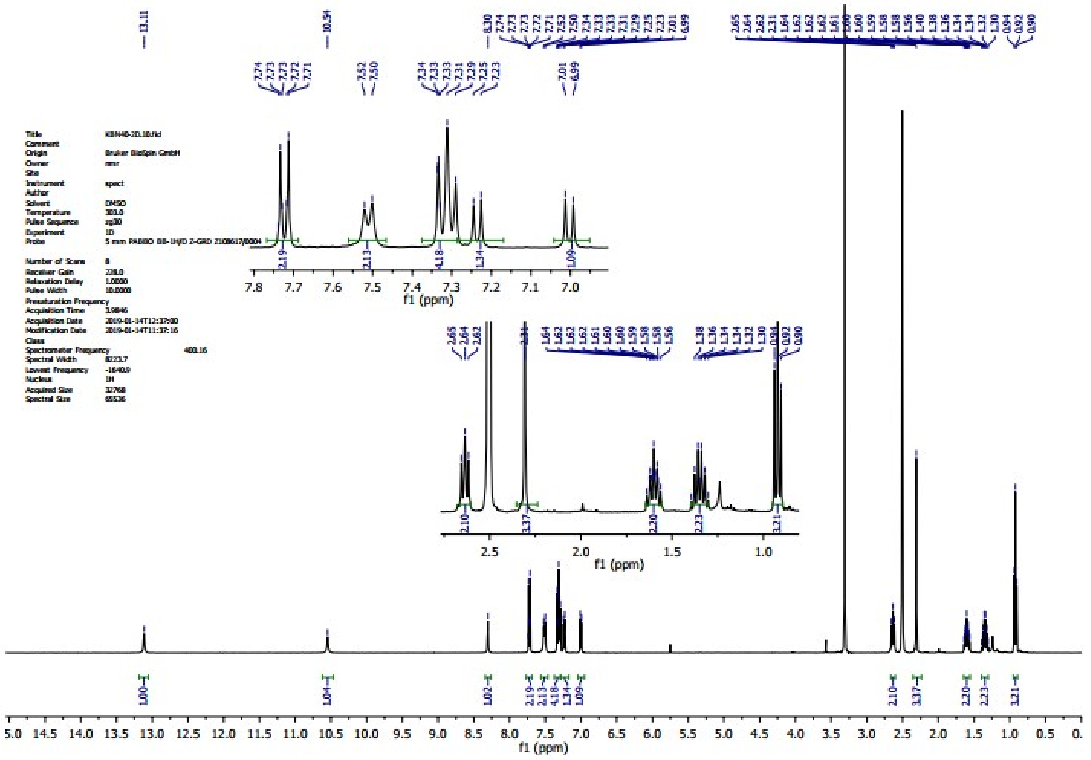

^{13}C NMR (75 MHz, DMSO-d_6)

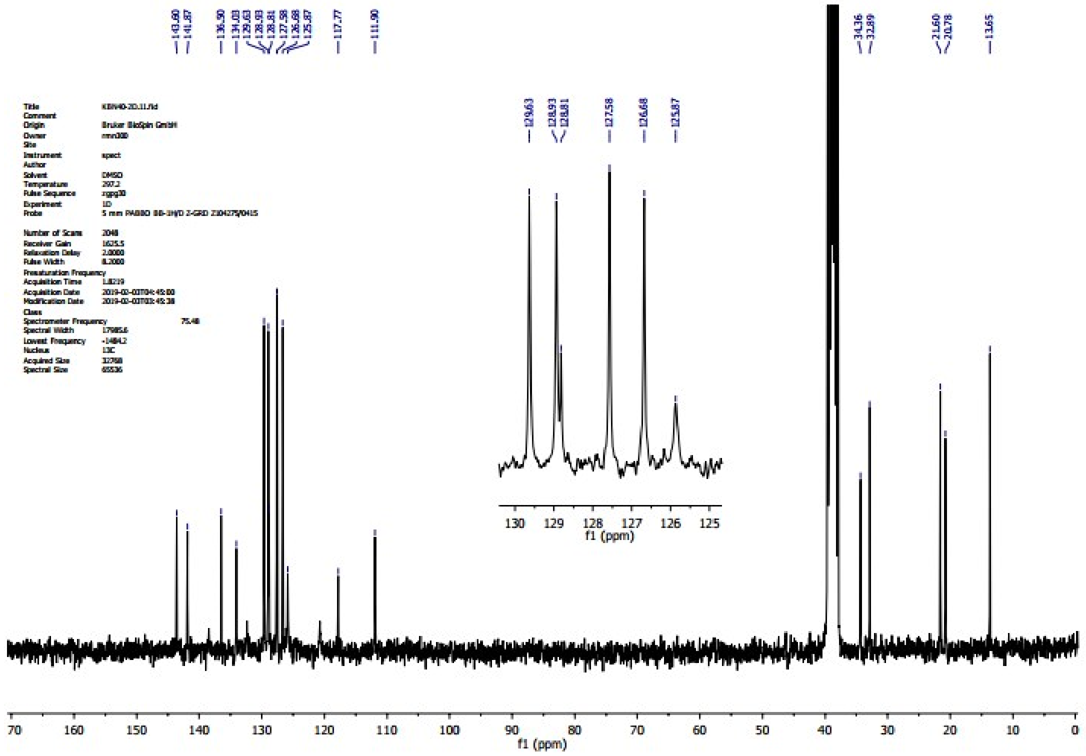

Compound 8ab

^{1}H NMR (300 MHz, DMSO-d_6)

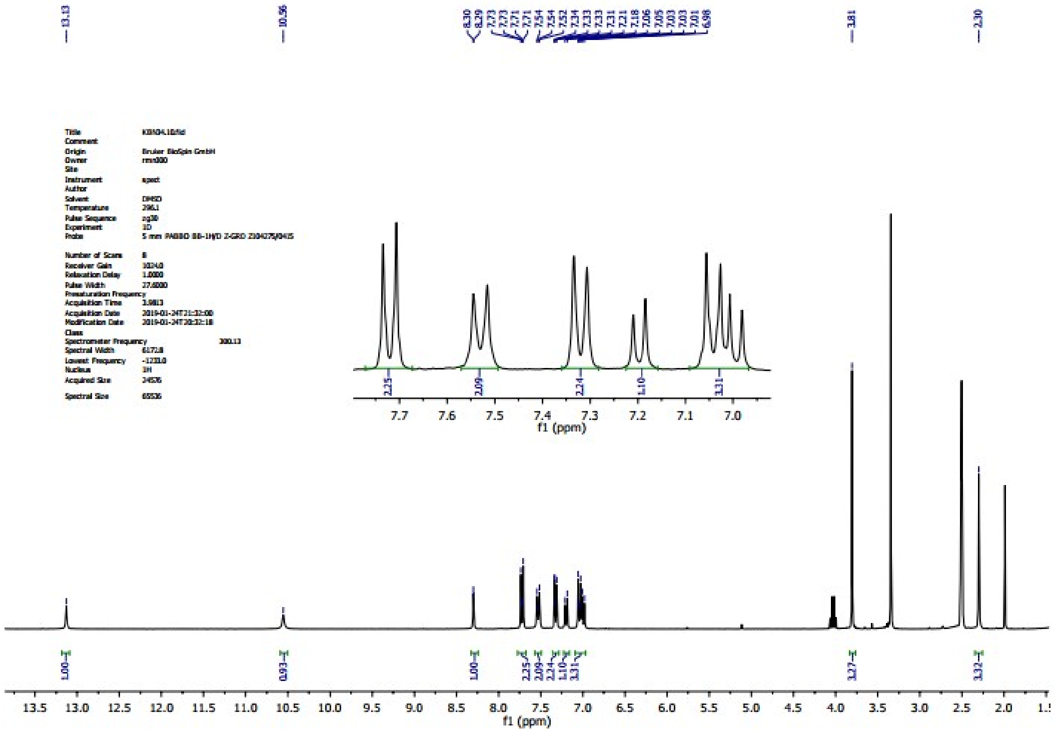

^{13}C NMR (75 MHz, DMSO-d_6)

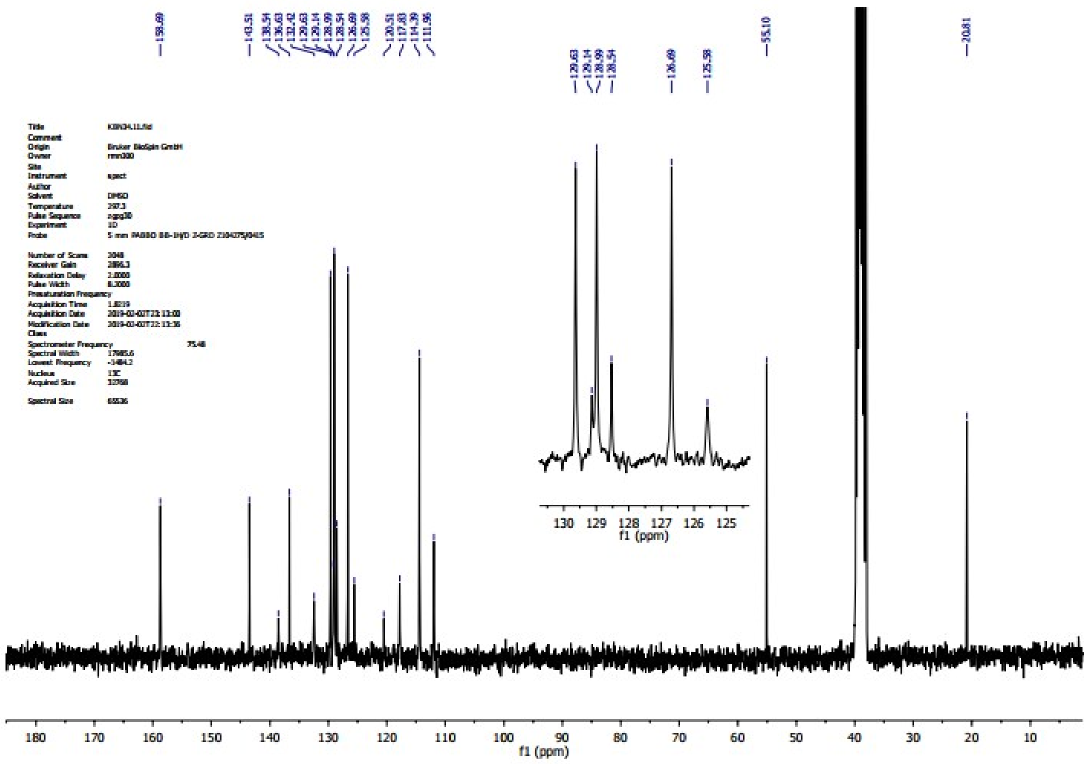

Compound 8ac

^{1}H NMR (400 MHz, DMSO-d_6)

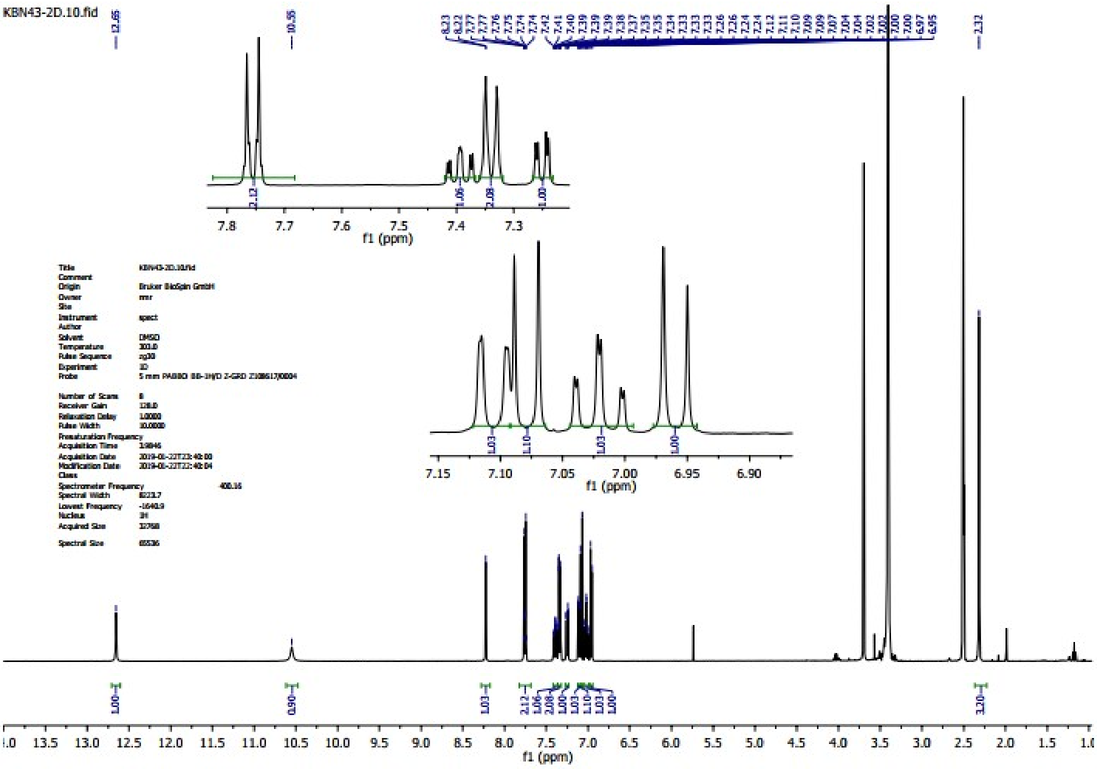

^{13}C NMR (75 MHz, DMSO-d_6)

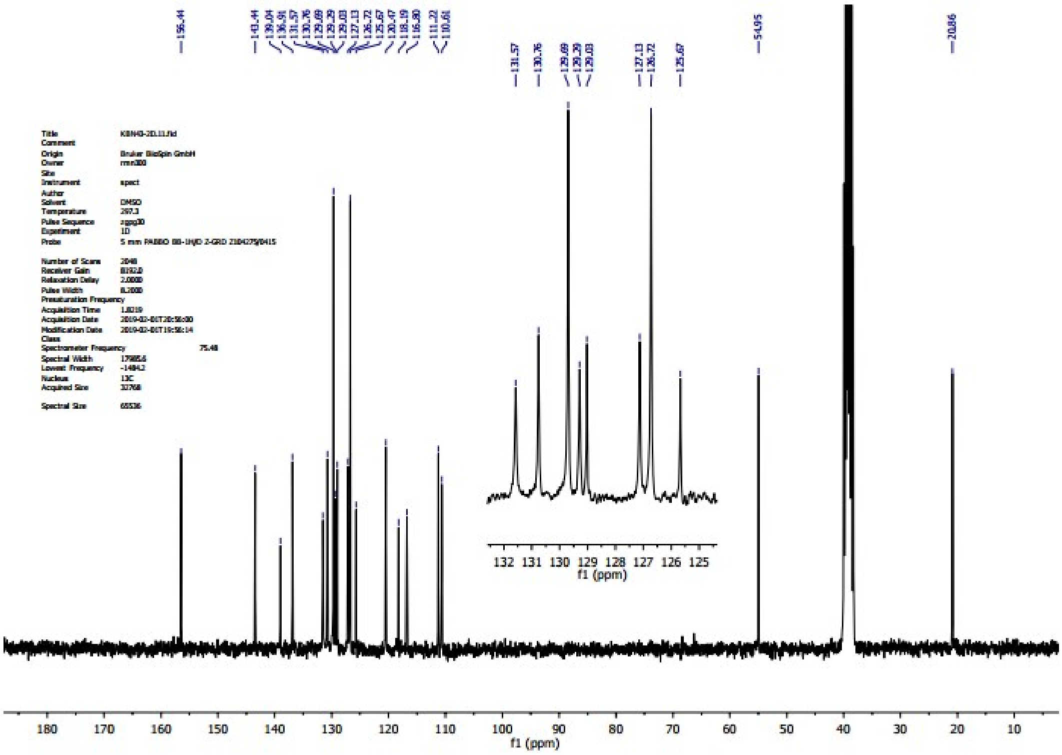

Compound 8ad:

^{1}H NMR (400 MHz, DMSO-d_6)

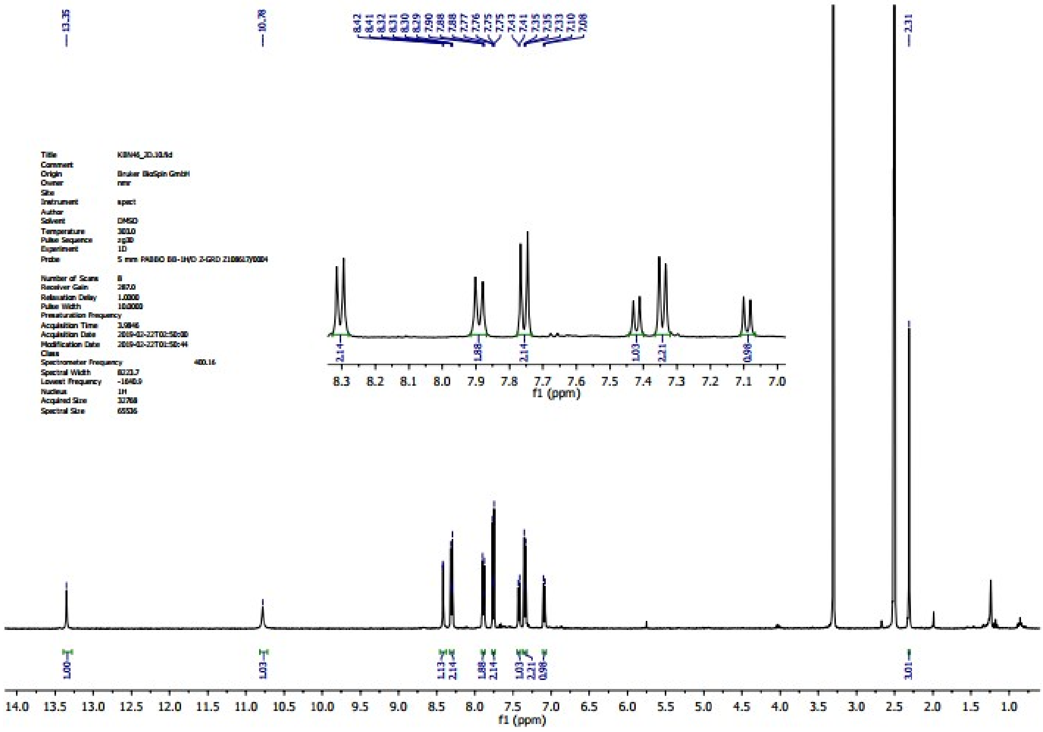

^{13}C NMR (101 MHz, DMSO-d_6)

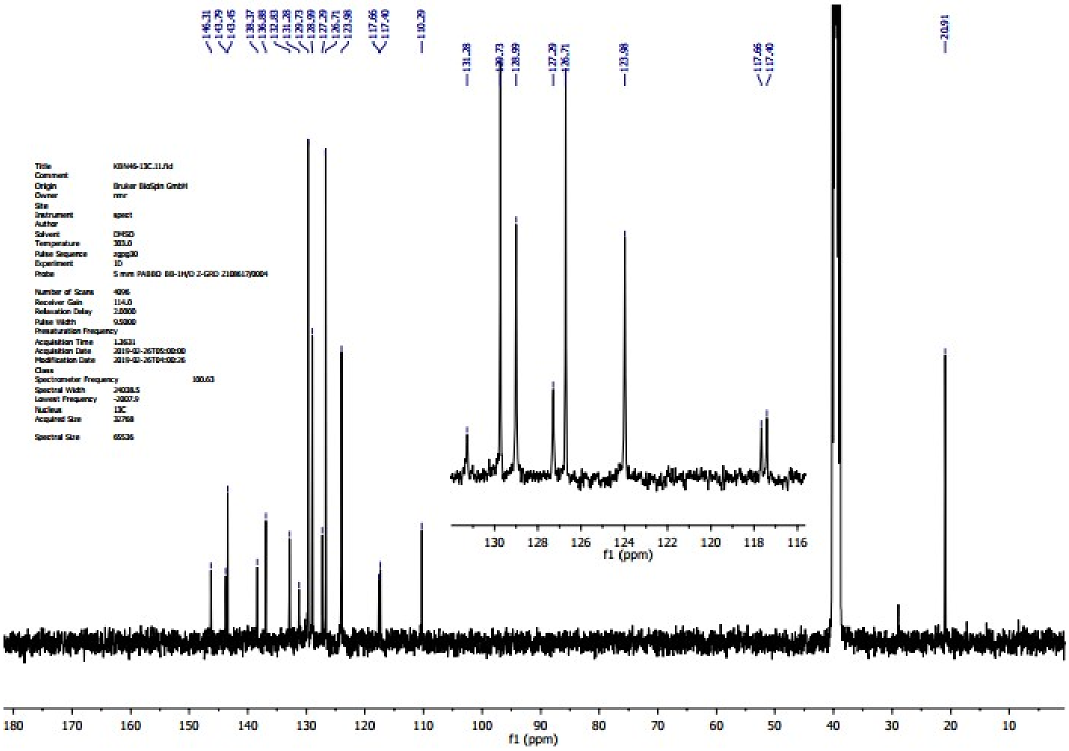

Compound 8ae

^{1}H NMR (400 MHz, DMSO-d_6)

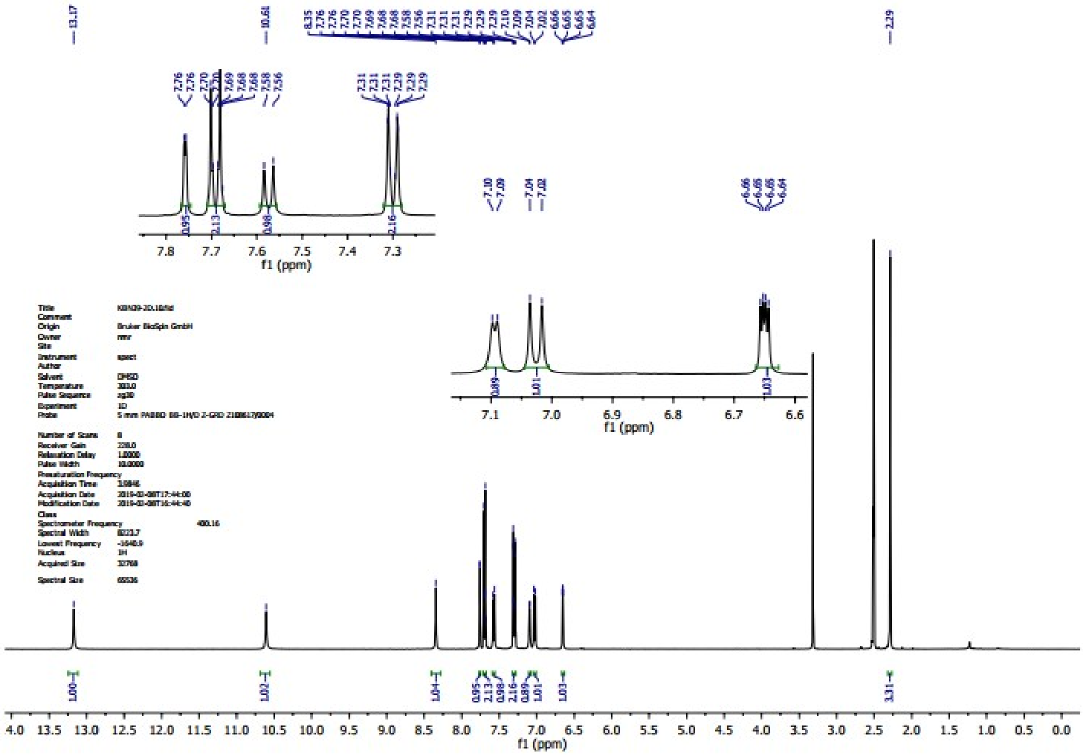

^{13}C NMR (101 MHz, DMSO-d_6)

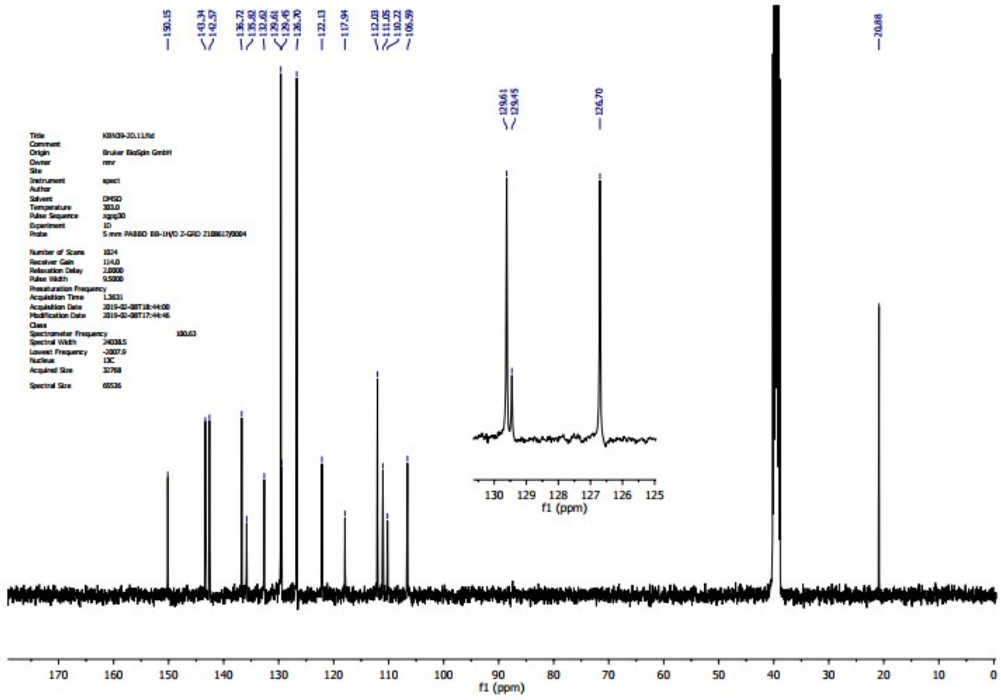

Compound 8af

^{1}H NMR (300 MHz, DMSO-d_6)

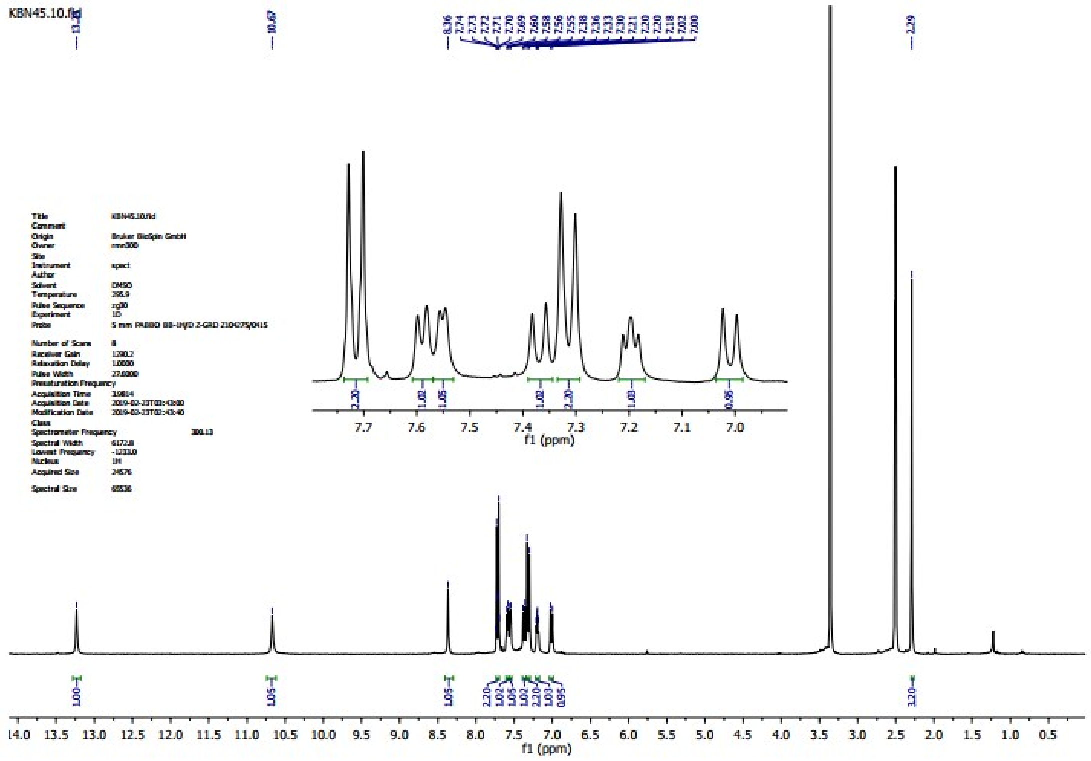

^{13}C NMR (75 MHz, DMSO-d_6)

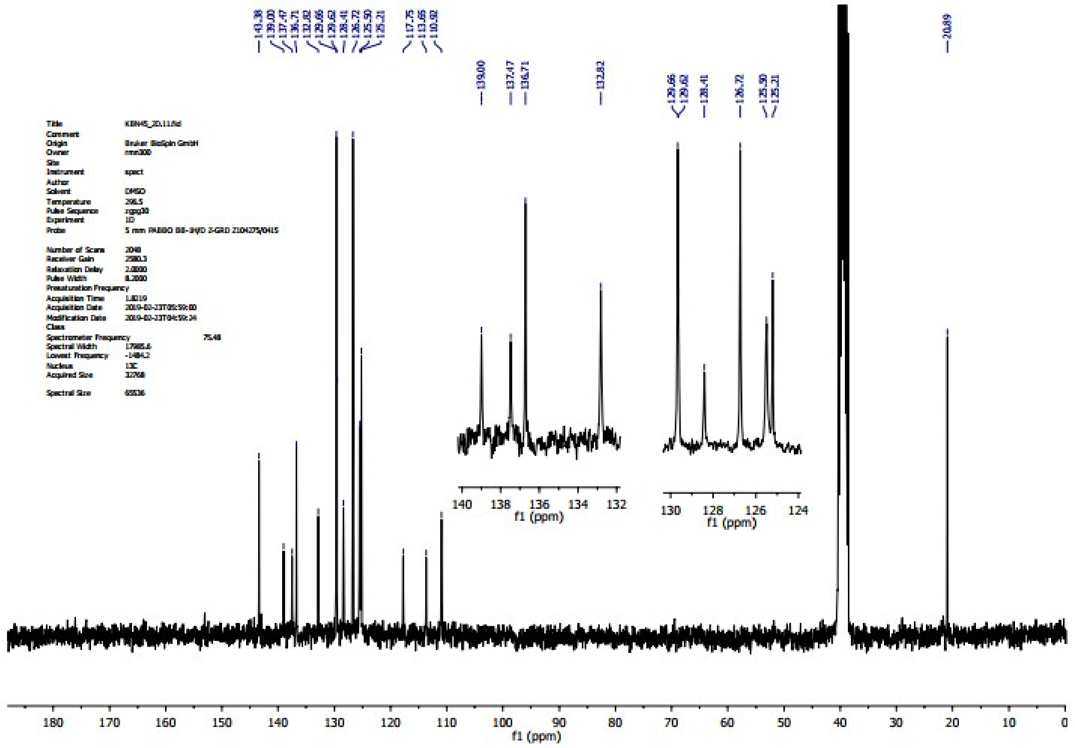

Compound 8ba

^{1}H NMR (300 MHz, DMSO-d_6)

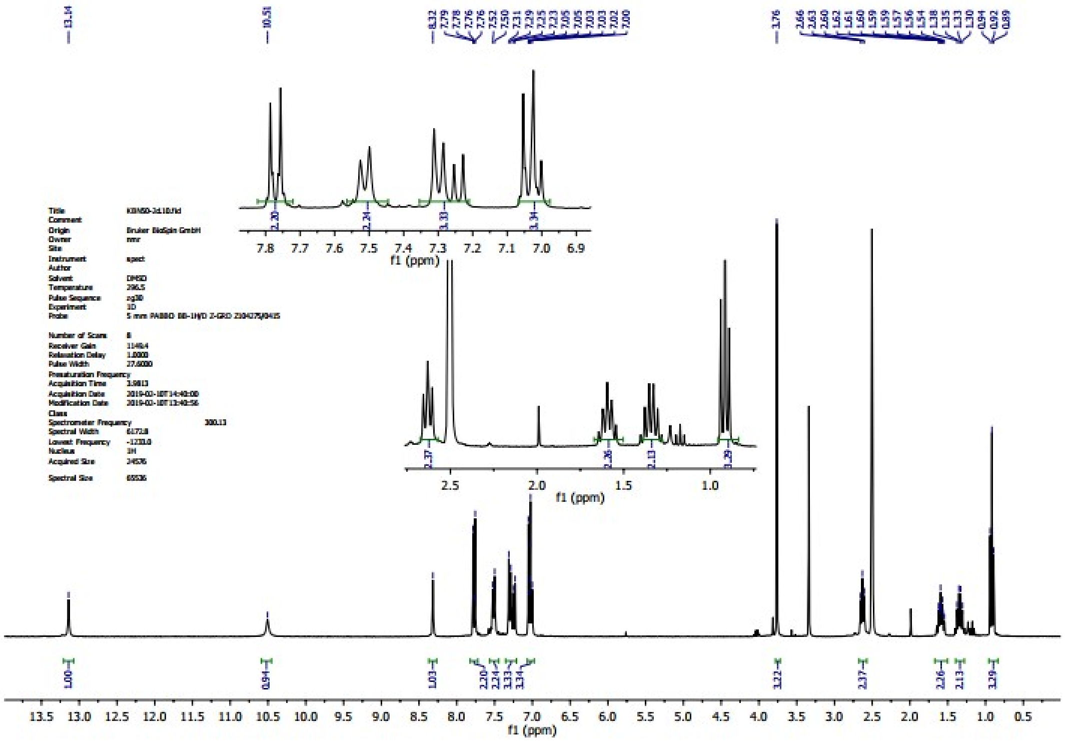

^{13}C NMR (75 MHz, DMSO-d_6)

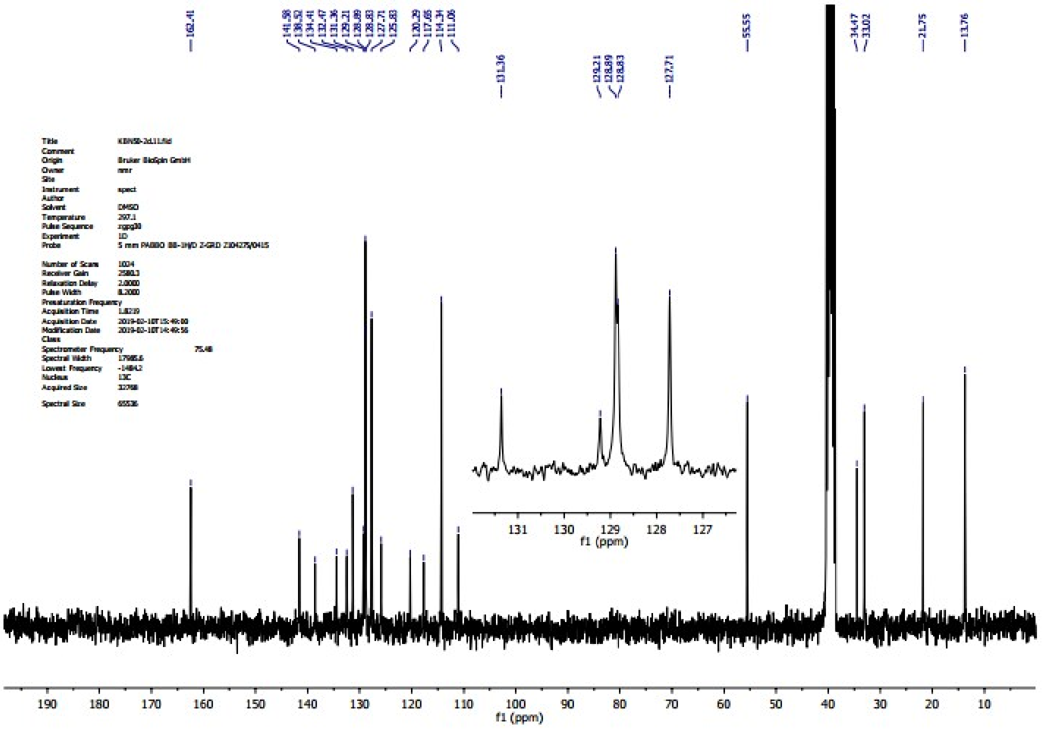

Compound 8bb

^{1}H NMR (300 MHz, DMSO-d_6)

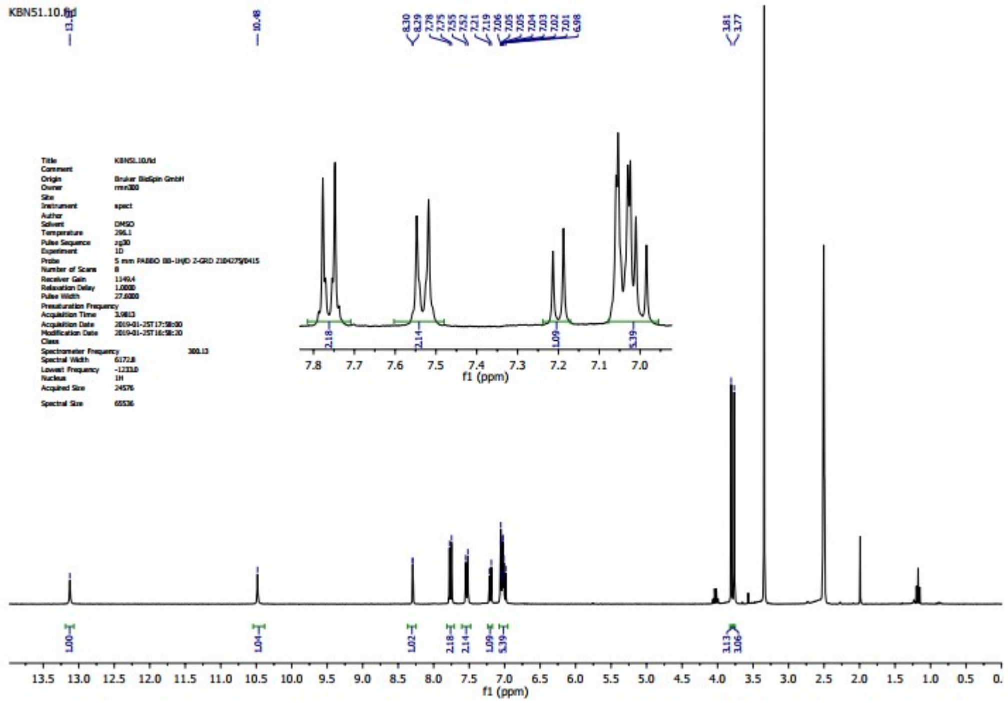

^{13}C NMR (75 MHz, DMSO-d_6)

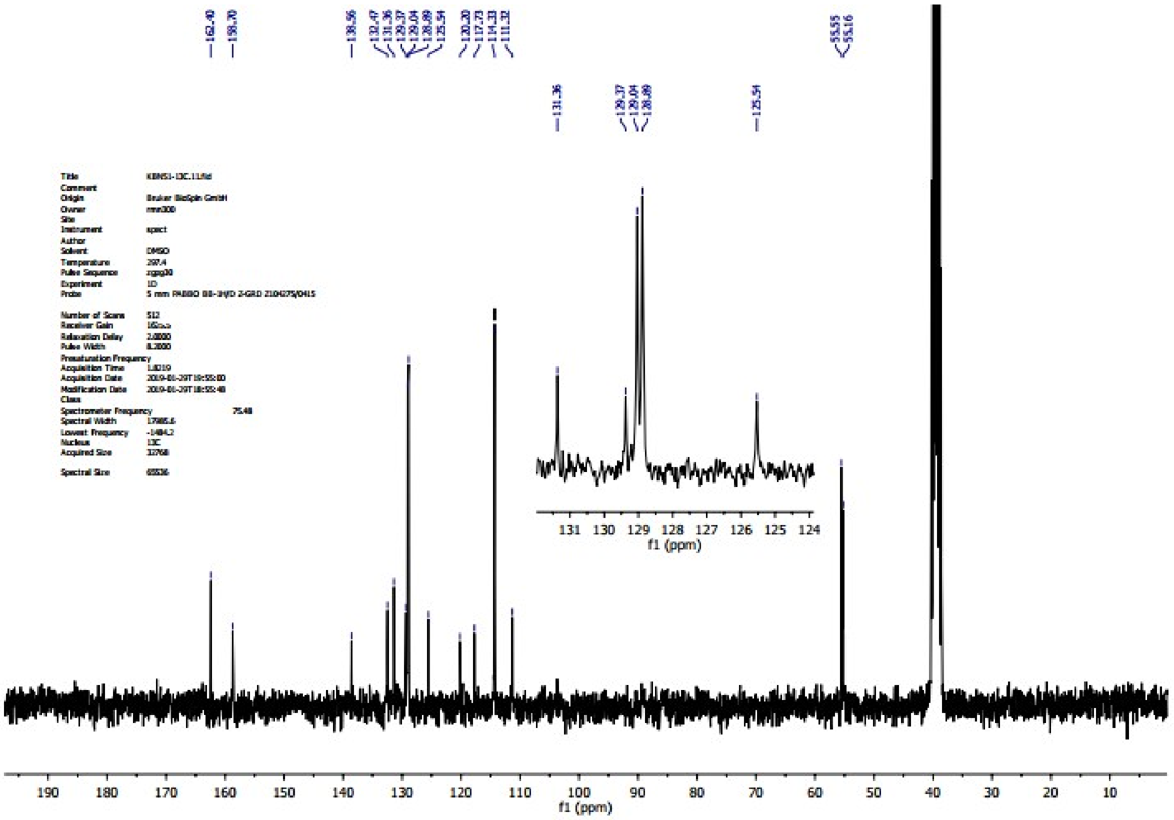

Compound 8bc

^{1}H NMR (300 MHz, DMSO-d_6)

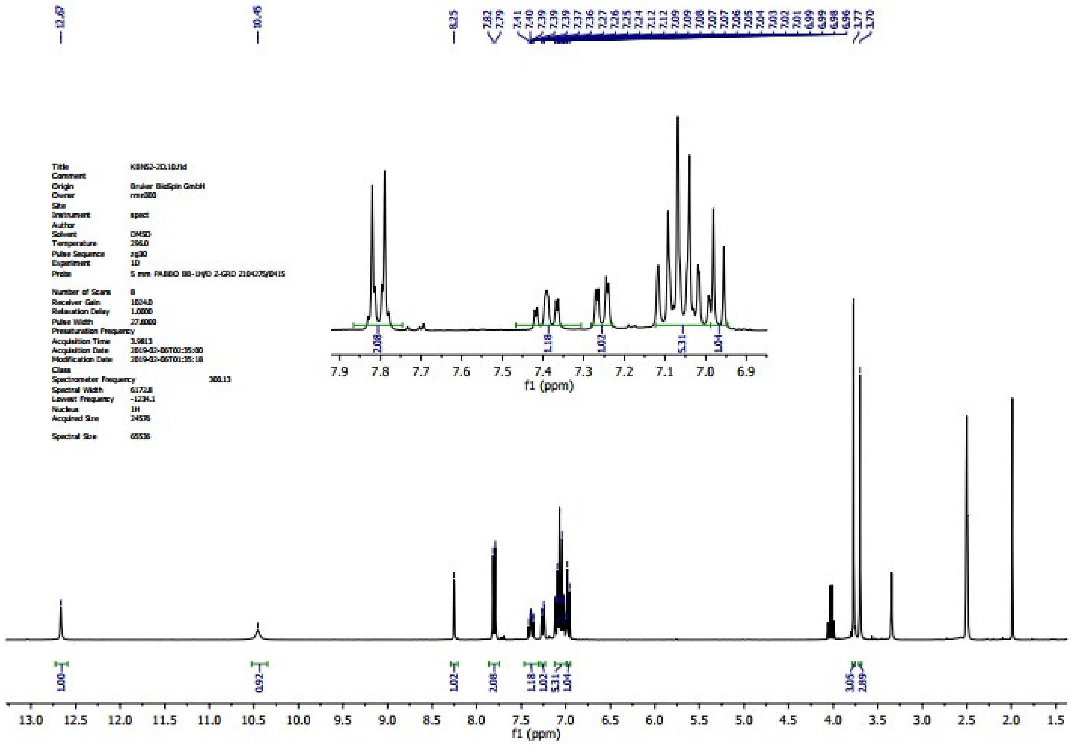

^{13}C NMR (75 MHz, DMSO-d_6)

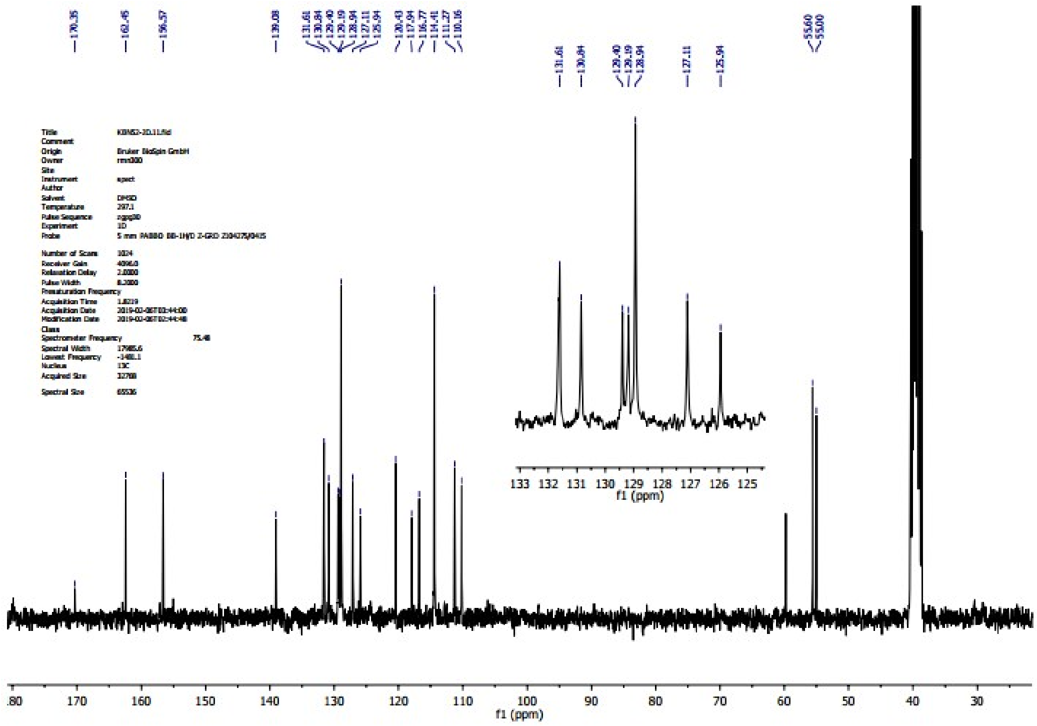

Compound 8bd

^{1}H NMR (300 MHz, DMSO-$_{d6}$)

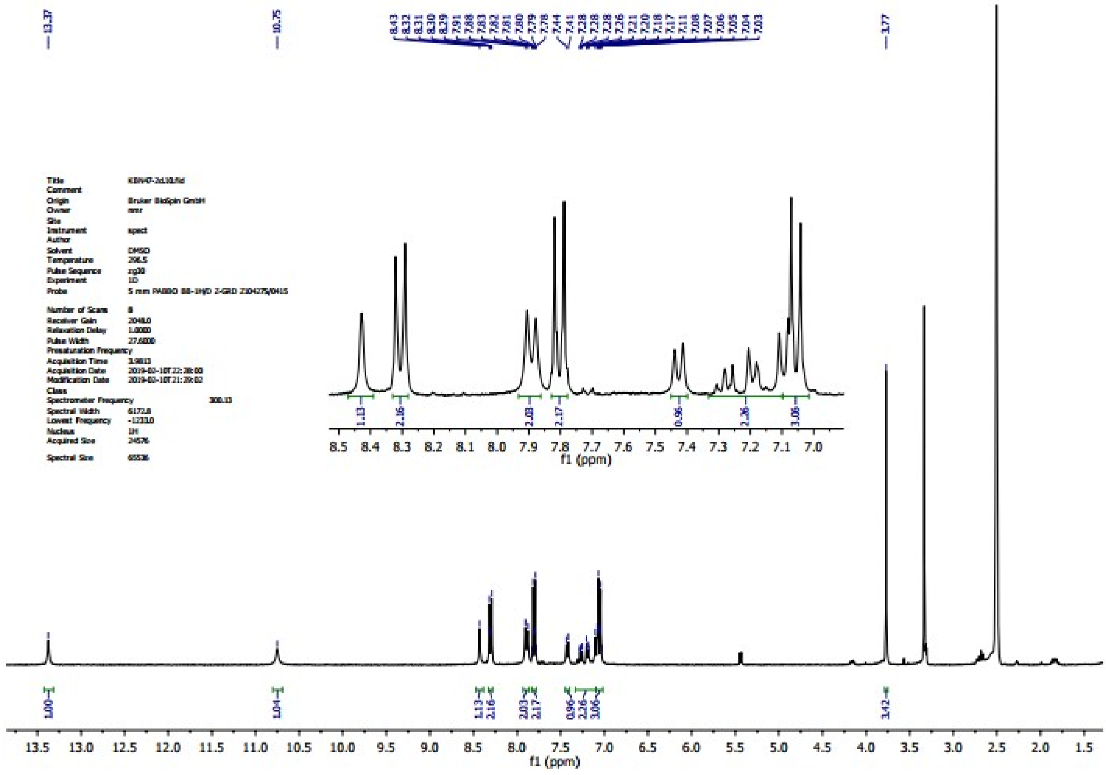

^{13}C NMR (75 MHz, DMSO-d_6)

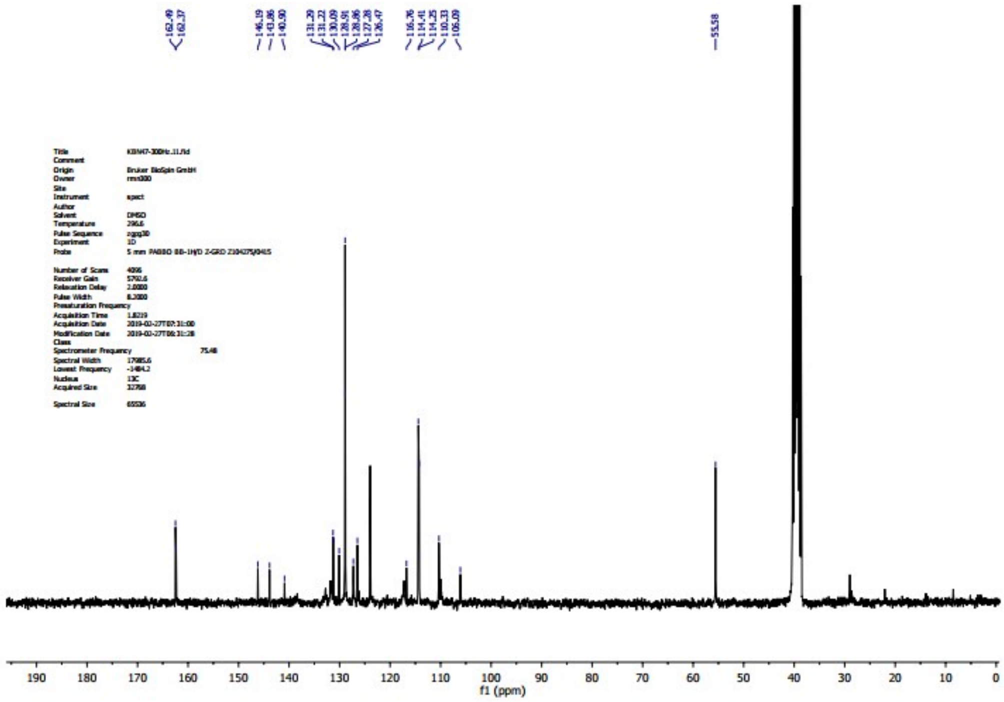

Compound 8be

^{1}H NMR (300 MHz, DMSO-d_6)

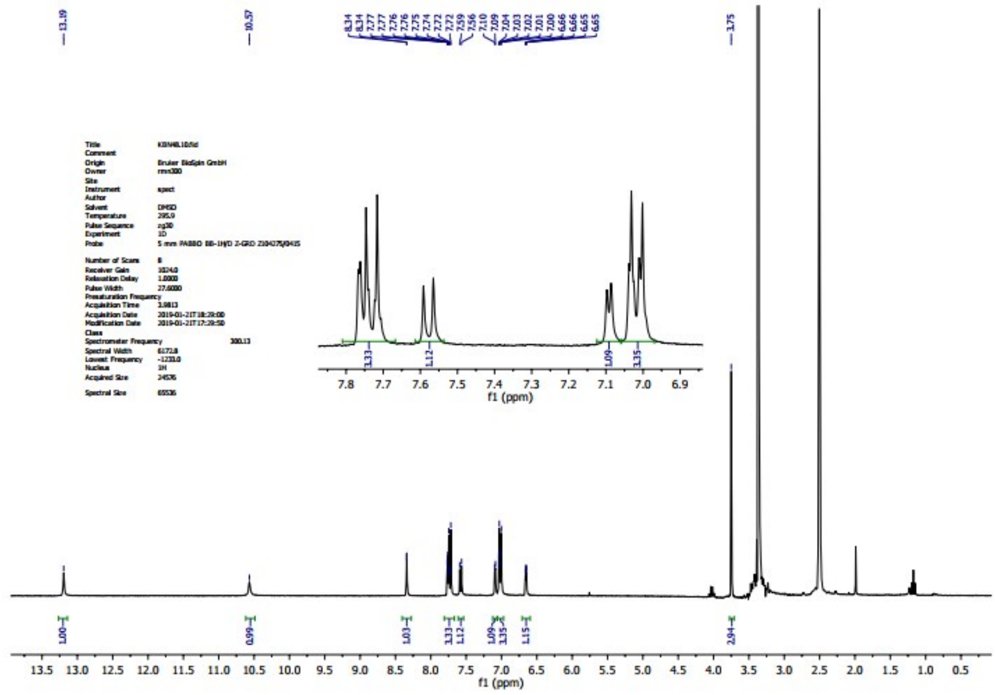

^{13}C NMR (101 MHz, DMSO-d_6)

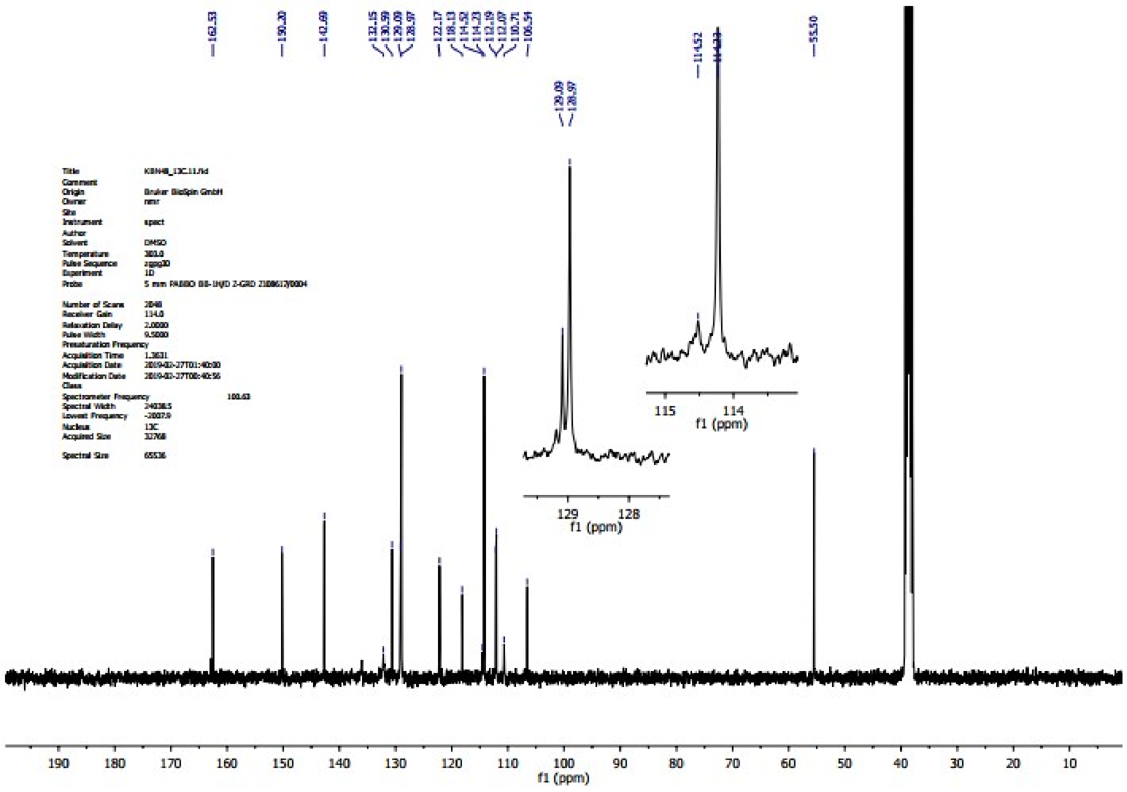

Compound 8cb

^{1}H NMR (300 MHz, DMSO-d_6)

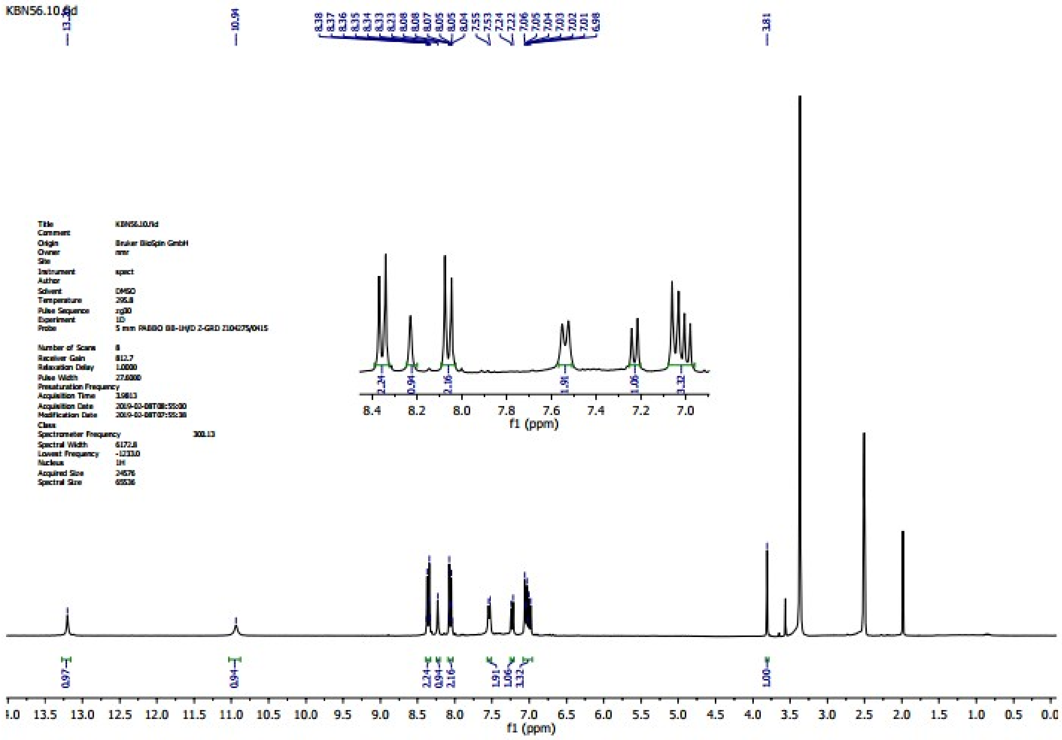

^{13}C NMR (101 MHz, DMSO-d_6)

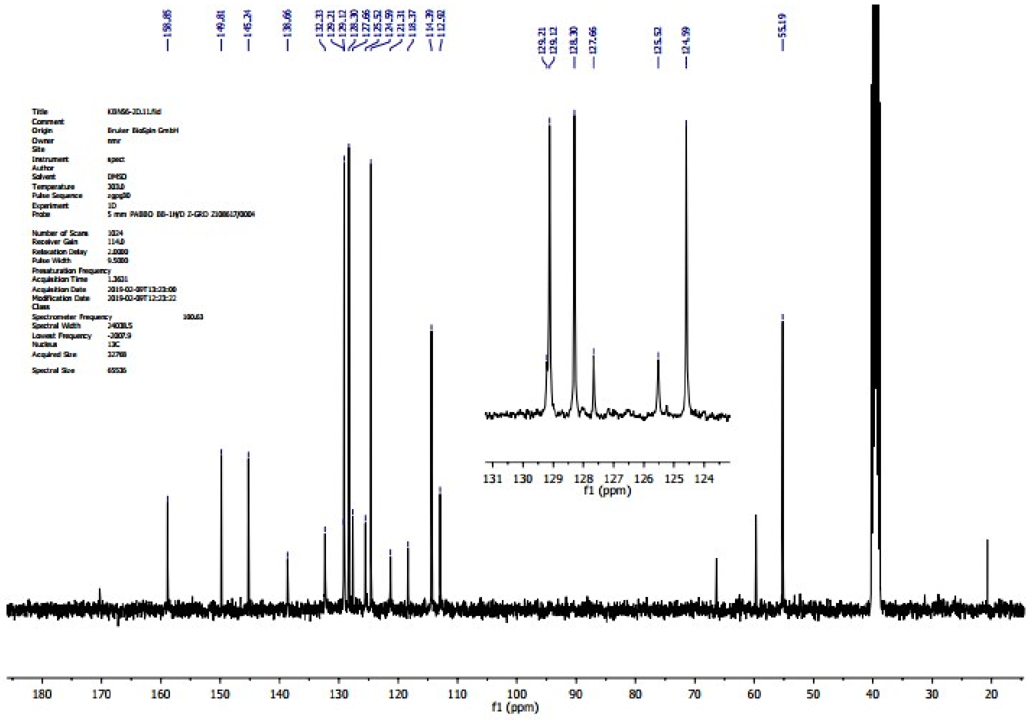

Compound 9ab

^{1}H NMR (300 MHz, DMSO-d_6)

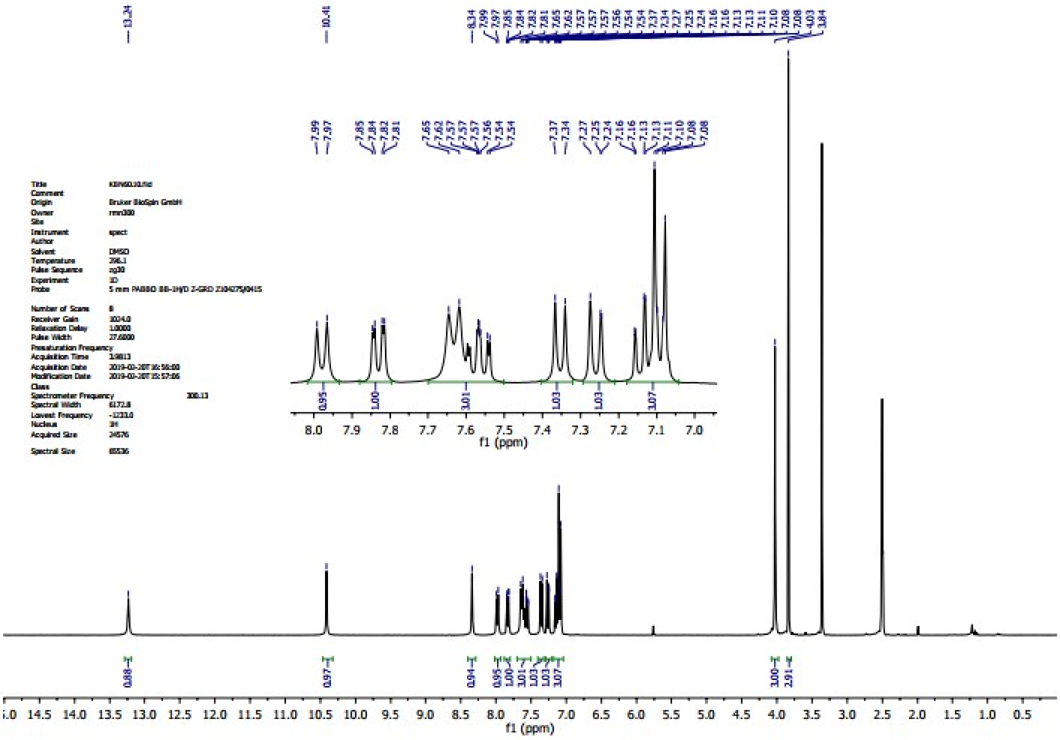

^{13}C NMR (101 MHz, DMSO-d_6)

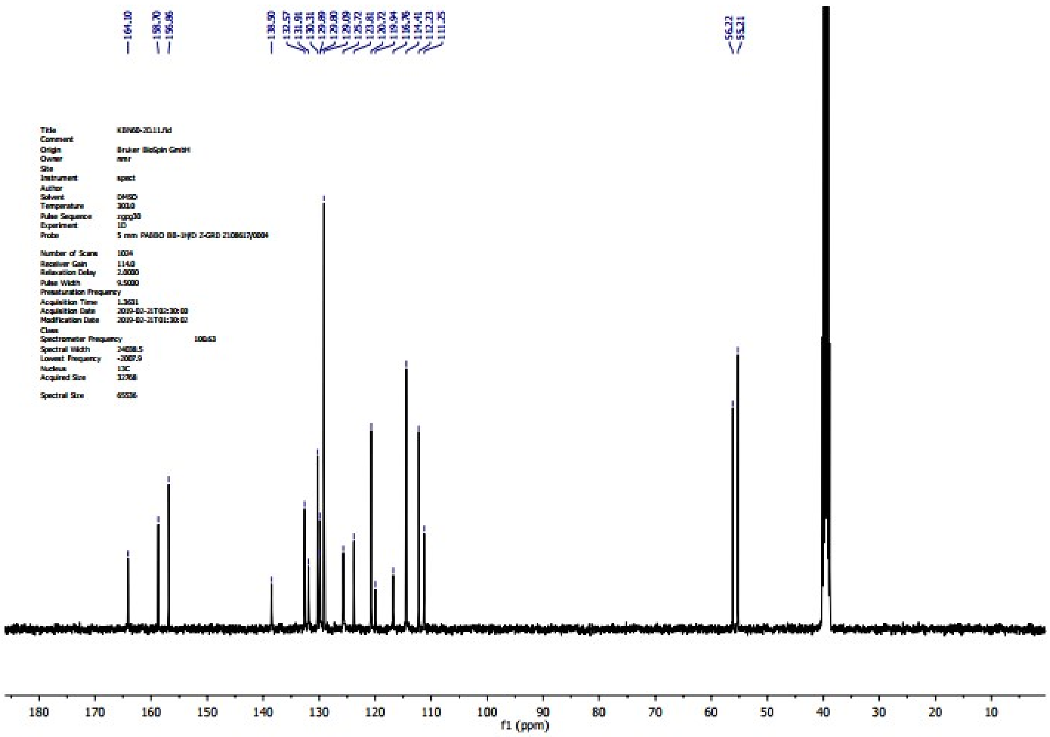

Compound 9ba:

^{1}H NMR (250 MHz, DMSO-d_6)

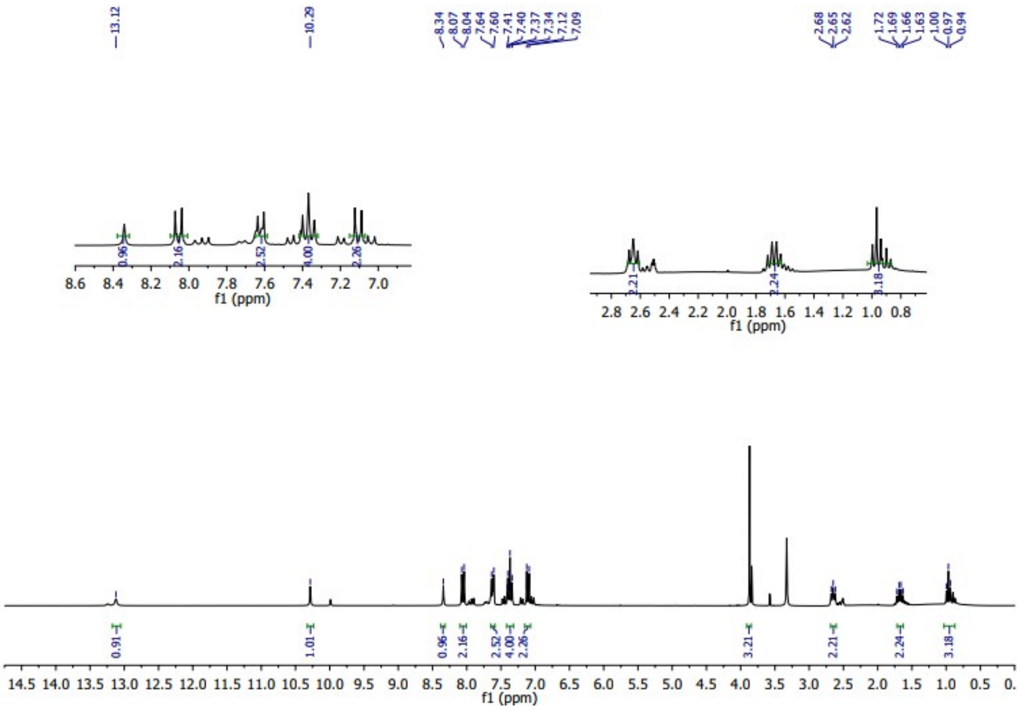

^{13}C NMR (65 MHz, DMSO-d_6)

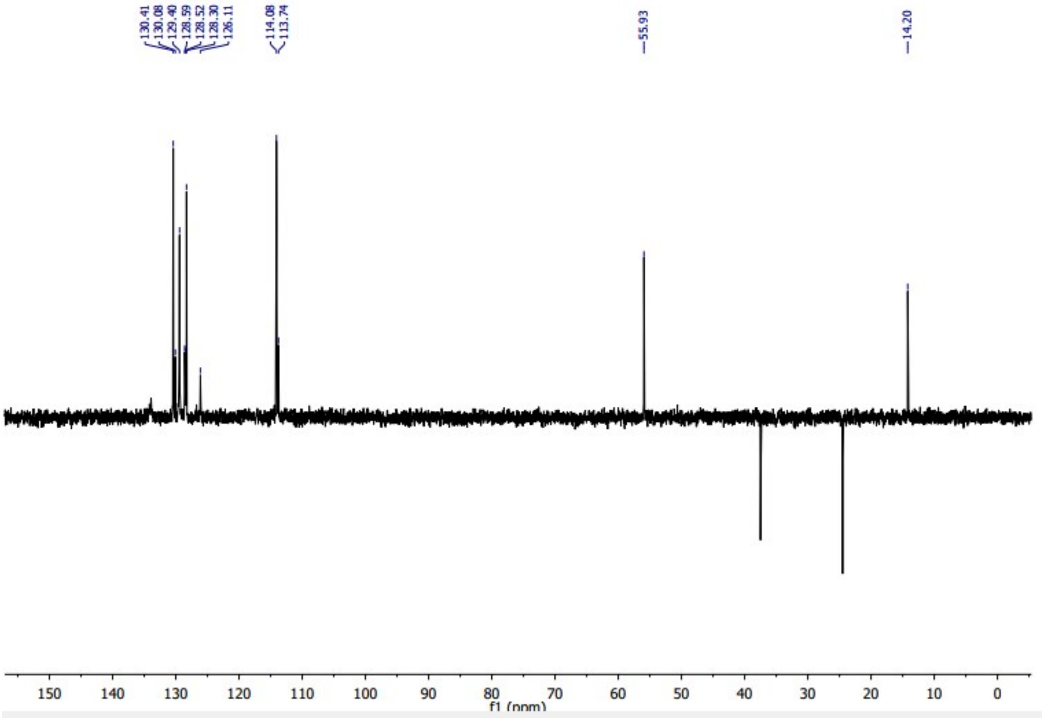

Compound 9bb

^{1}H NMR (300 MHz, DMSO-d_6)

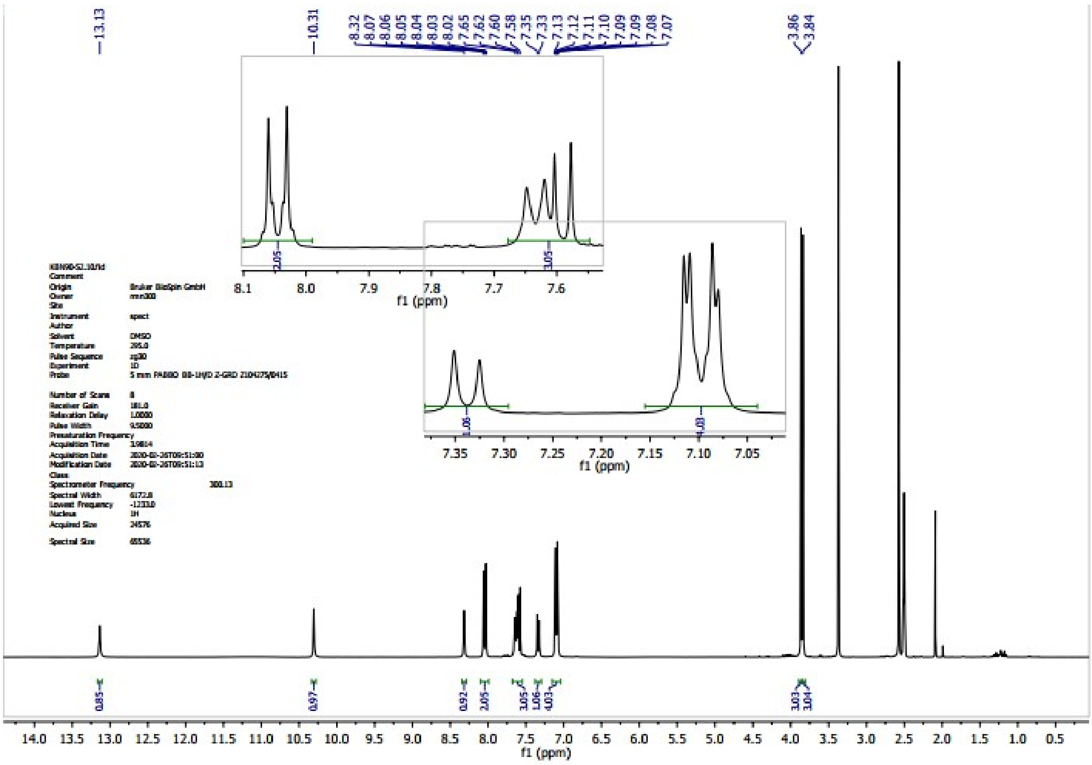

^{13}C NMR (101 MHz, DMSO-d_6)

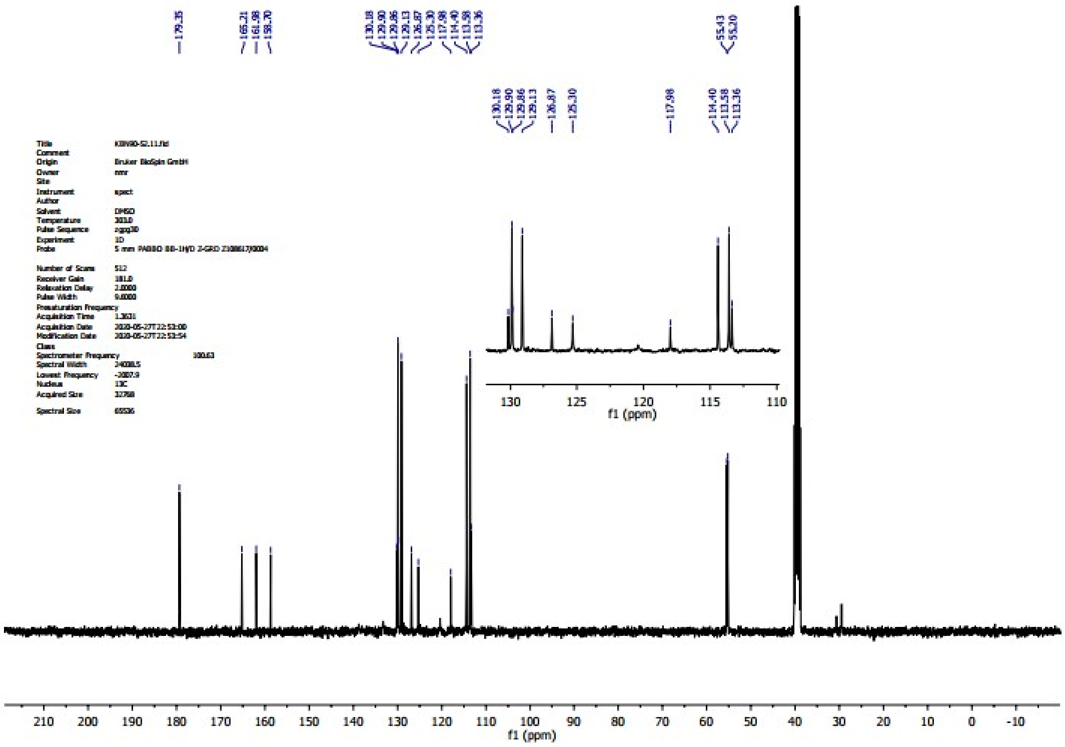

Compound 9bd

^{1}H NMR (300 MHz, DMSO-d_6)

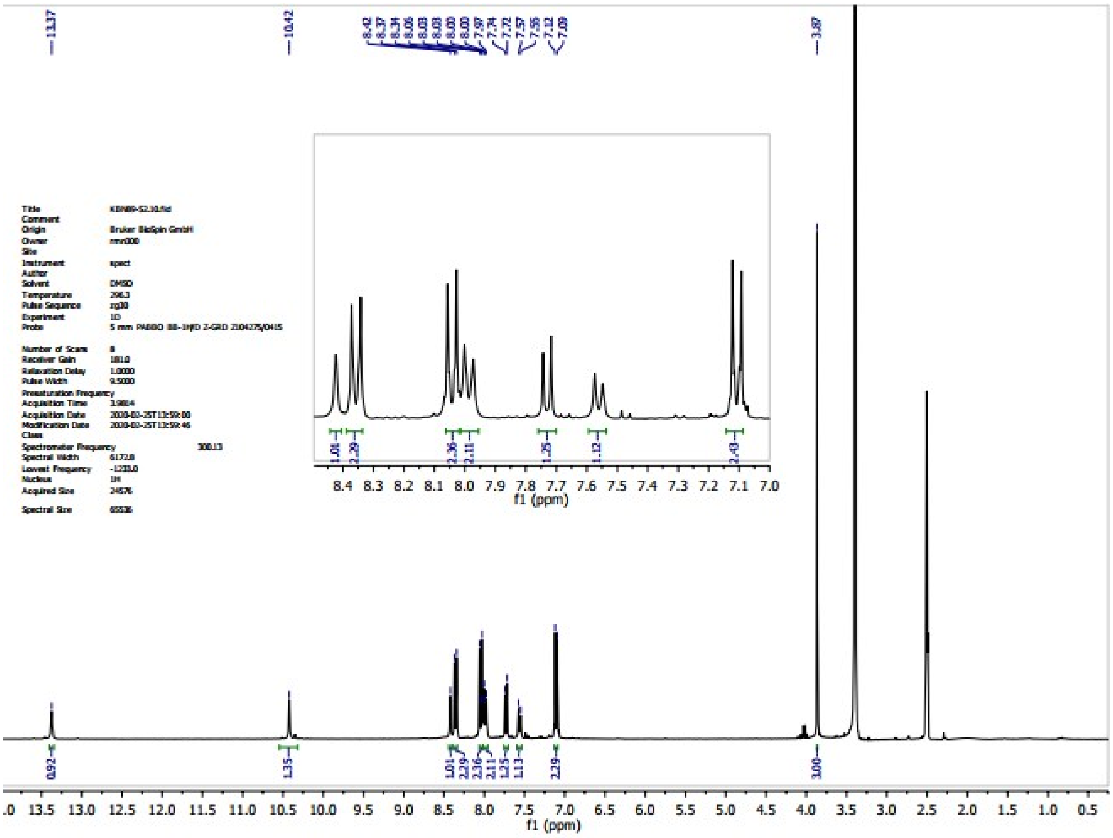

^{13}C NMR (101 MHz, DMSO-d_6)

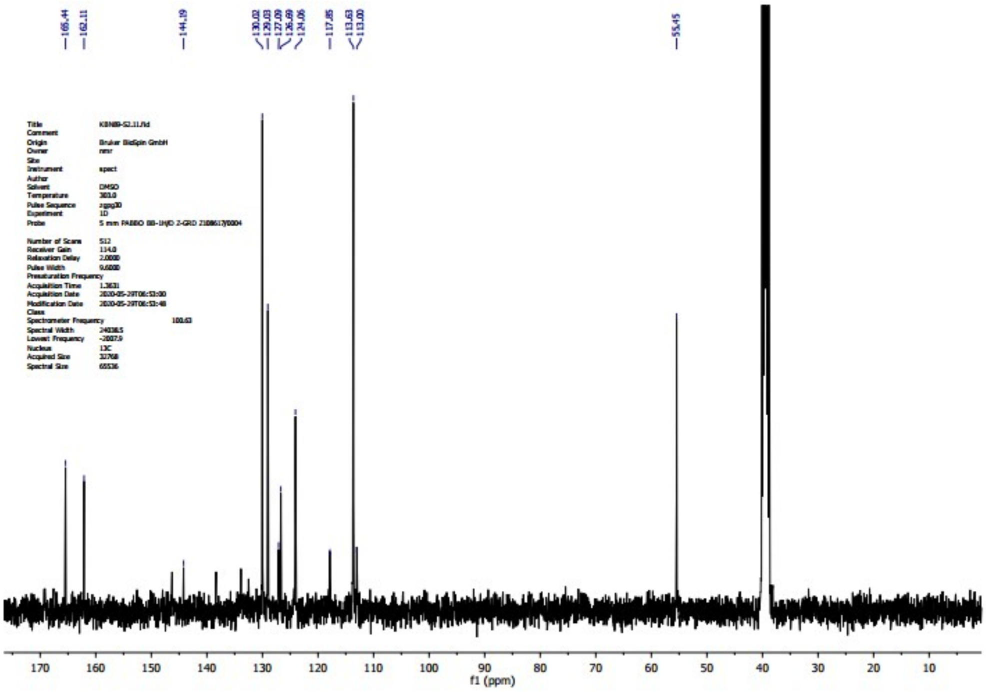

Compound 9be:

^{1}H NMR (300 MHz, DMSO-d_6)

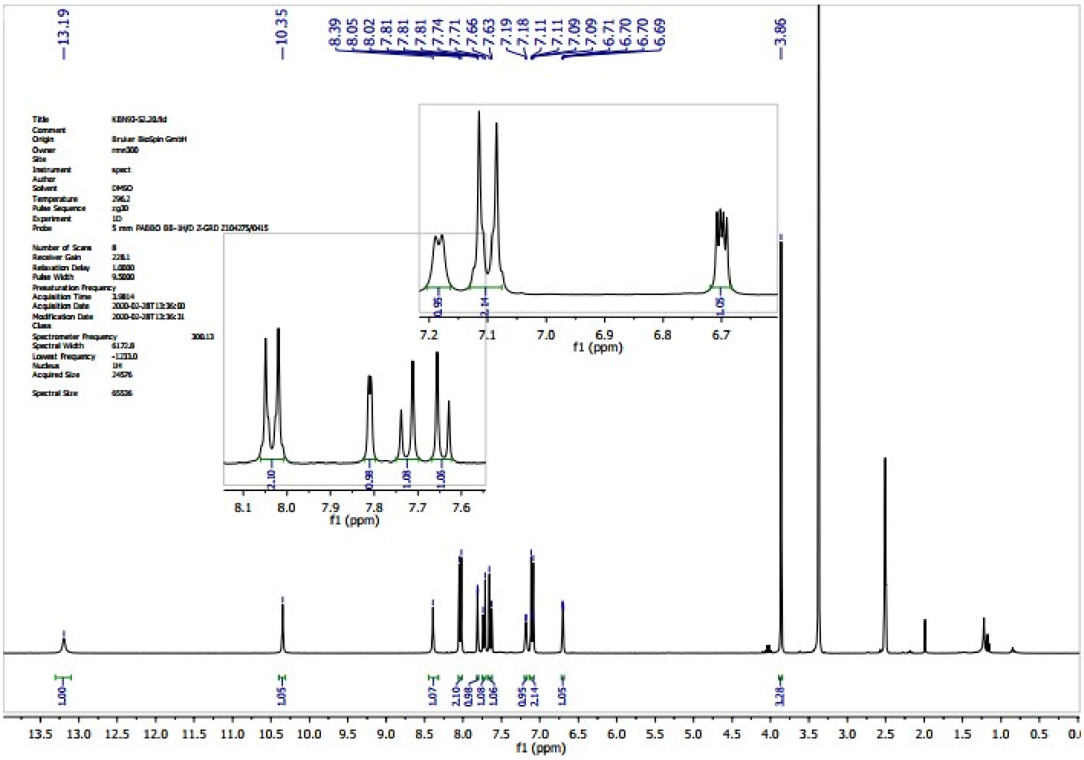

^{13}C NMR (101 MHz, DMSO-d_6)

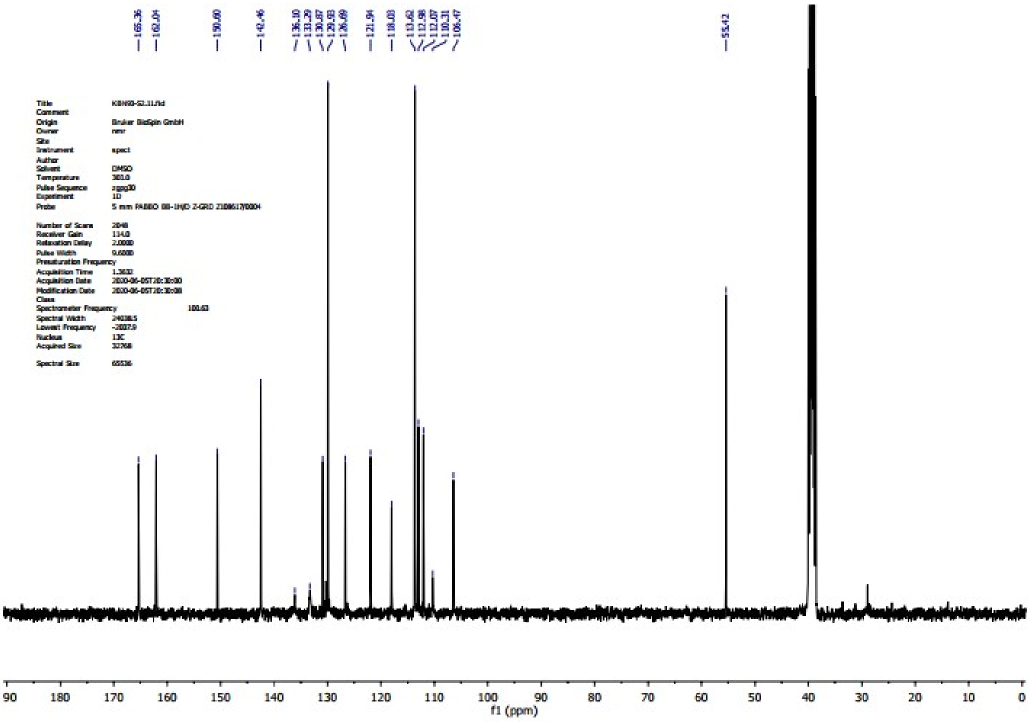

Compound 9bf:

^{1}H NMR (250 MHz, DMSO-d_6)

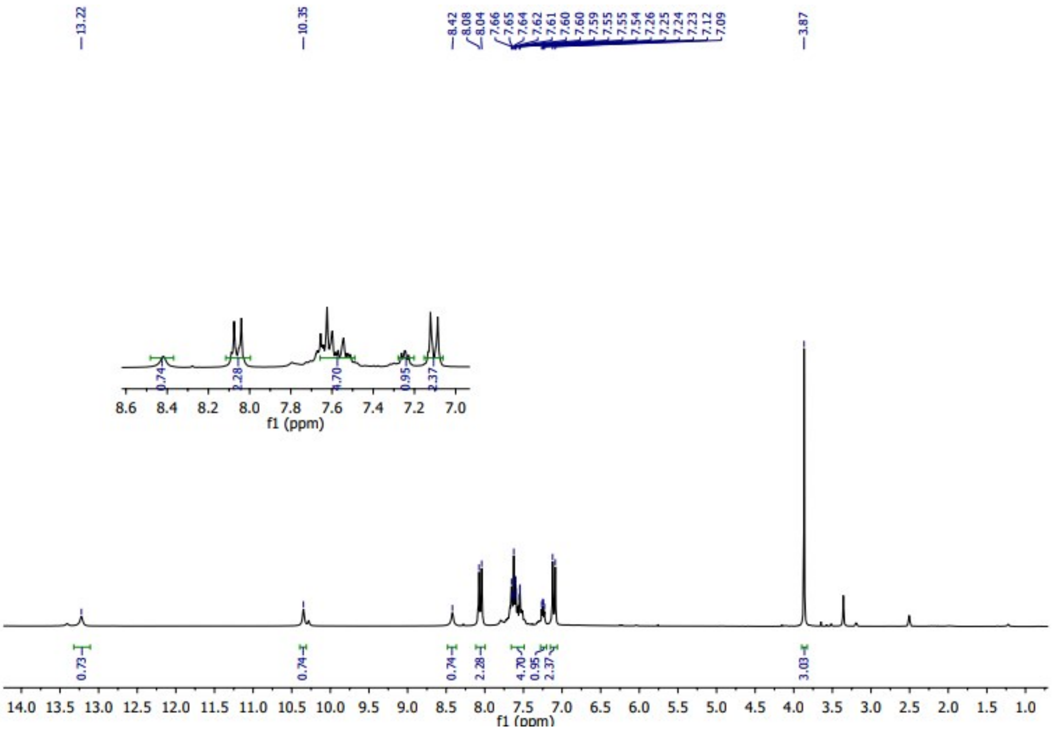

^{13}C NMR (65 MHz, DMSO-d_6)

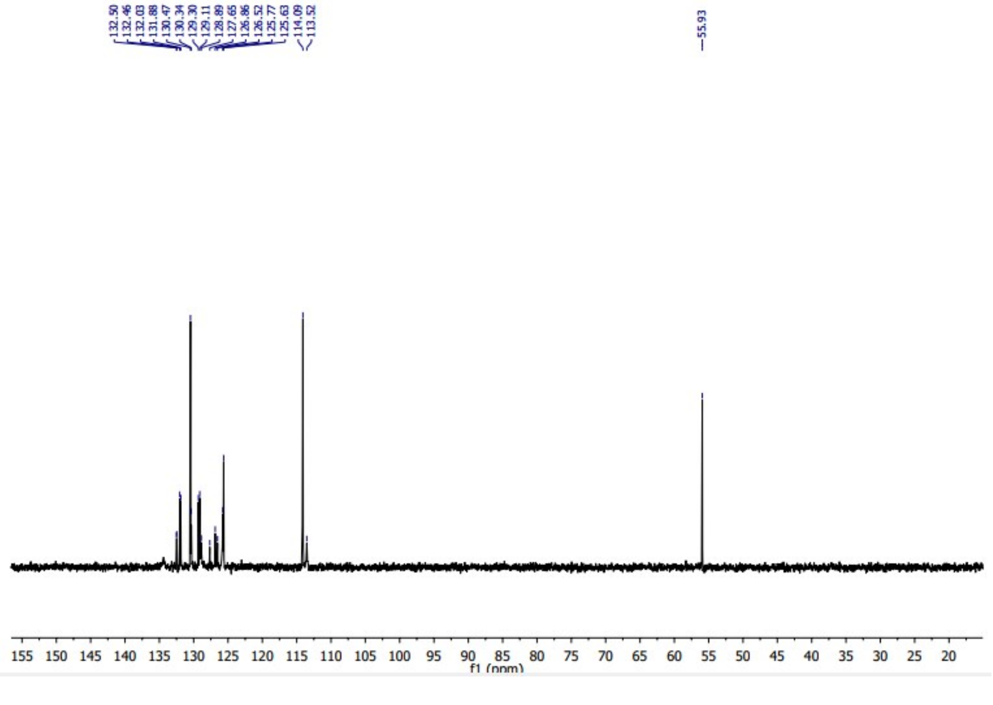

***Compound 9ca*:**

^{1}H NMR (400 MHz, DMSO-d_6)

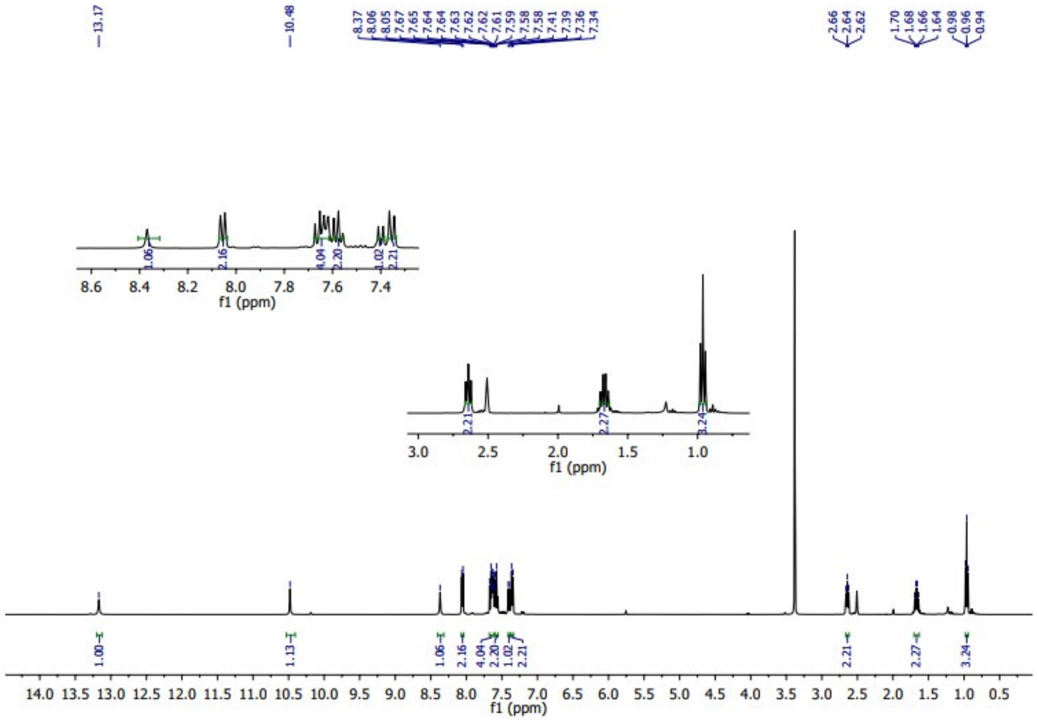

^{13}C NMR (101 MHz, DMSO-d_6)

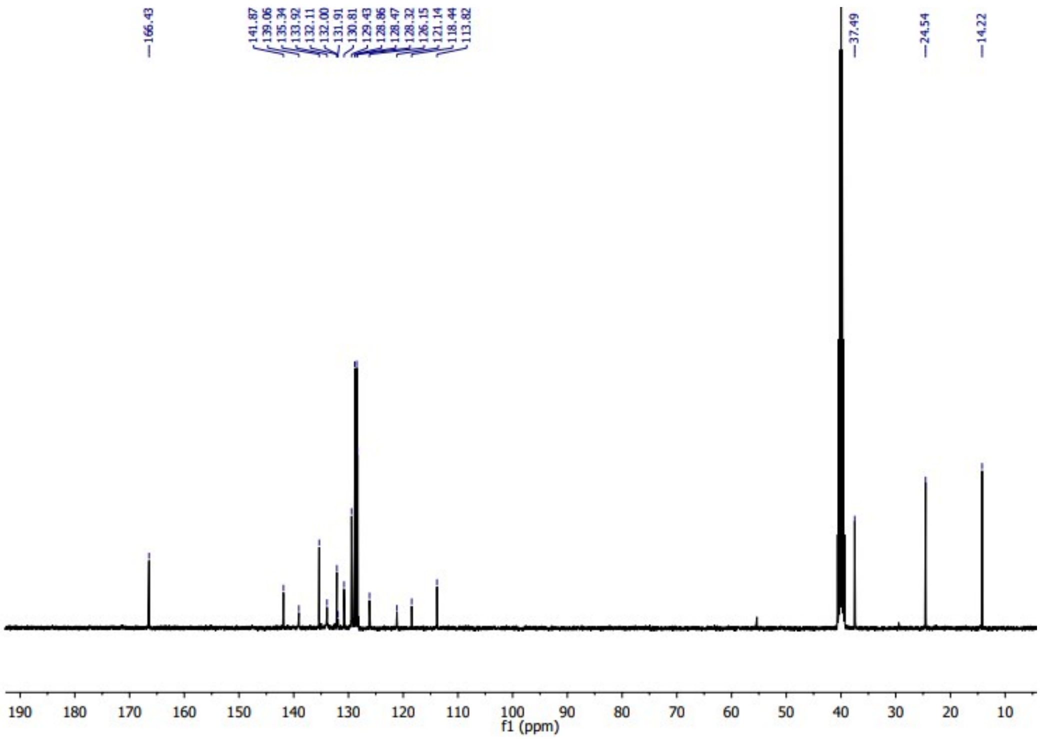

***Compound 9cg*:**

^1H NMR (400 MHz, DMSO-d_6)

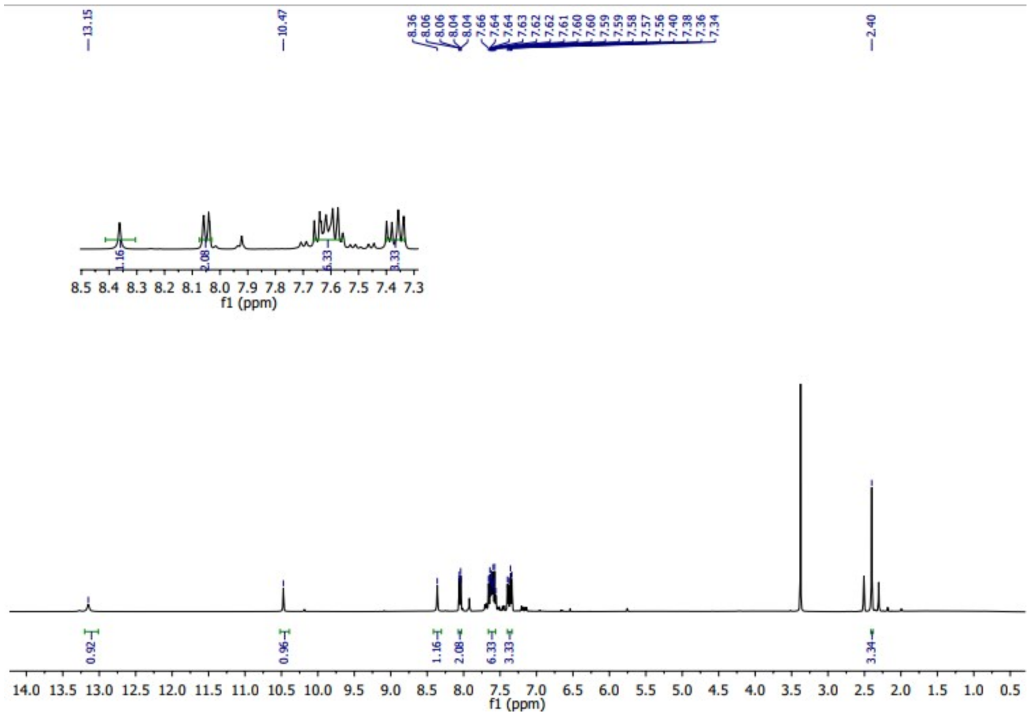

^{13}C NMR (101 MHz, DMSO-d_6)

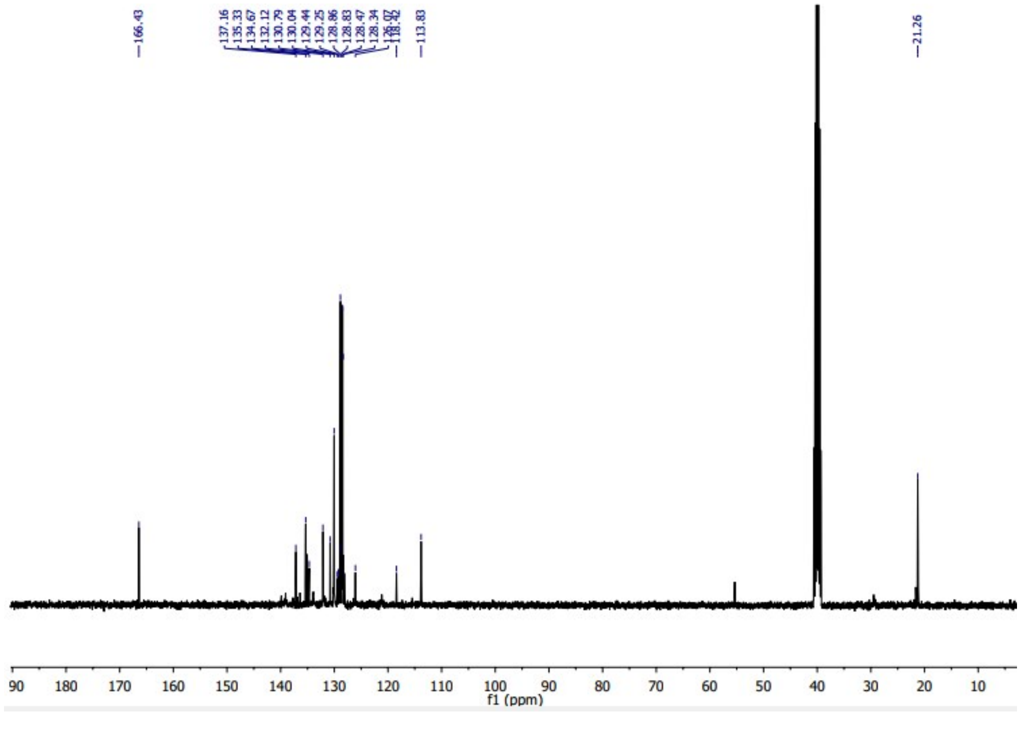

Supplement: RA-011-D0RA08598G-s001 [file RA-011-D0RA08598G-s001.pdf]
